# Cold activates a BH2 metabolic network promoting longevity and proteostasis

**DOI:** 10.1038/s41586-026-11152-9

**Published:** 2026-10-21

**Authors:** Hyun Ju Lee, Cansu Doğan, Seda Koyuncu, Markus Wehrmann, Murat Artan, Dunja Petrovic, Rute Loureiro, Amit Kumar Sahu, Joana Schnubel, Pavel Grobushkin, Dimitrios Prymidis, Theodoros Georgomanolis, Agnieszka Sokol, Ming Yang, Thorsten Hoppe, Christian Frezza, David Vilchez

**Affiliations:** 1 Institute for Integrated Stress Response Signaling, Faculty of Medicine, University Hospital Cologne, Cologne, Germany; 2 Cologne Excellence Cluster for Cellular Stress Responses in Aging-Associated Diseases (CECAD), University of Cologne, Cologne, Germany; 3 Institute for Genetics, Faculty of Mathematics and Natural Sciences, University of Cologne, Cologne, Germany; 4 Institute for Metabolomics in Ageing, Faculty of Medicine, University Hospital Cologne, Cologne, Germany; 5 Center for Molecular Medicine Cologne (CMMC), University of Cologne, Cologne, Germany

**Keywords:** Mechanisms of disease, Protein aggregation

## Abstract

Moderately cold temperatures extend lifespan and promote health across species^1–3^, yet the molecular mechanisms underlying these benefits remain unclear. Here we define a metabolite-driven, multi-tissue signalling system that links cold temperature to enhanced longevity and proteostasis in *Caenorhabditis elegans*. Cold temperature upregulates the biopterin-synthesis enzyme CAT-4 (GCH1 in humans), increasing production of dihydrobiopterin (BH2), a transportable reservoir for the bioactive cofactor tetrahydrobiopterin (BH4). BH2 is produced in muscle and transported to distal tissues, including the intestine and neurons, where it is converted into BH4 by the salvage enzyme DHFR-1. BH4 functions as an essential cofactor for phenylalanine hydroxylase PAH-1, promoting tyrosine synthesis and extending lifespan. This BH2–BH4 axis also prevents disease-related protein aggregation and subsequent neuronal dysfunction during ageing. Importantly, increasing BH2 synthesis specifically in muscle is sufficient to promote longevity and suppress pathological protein aggregation in distal tissues, even at normal or elevated temperatures. In human cells, BH2 or tyrosine supplementation reduces aggregation of polyQ-expanded proteins associated with Huntington’s disease, as well as mutant variants of TDP-43 and FUS that cause amyotrophic lateral sclerosis. Together, these findings identify BH2 as a cold-induced metabolite of which the synthesis, intertissue transport and conversion into bioactive BH4 preserve organism-wide proteostasis and extend lifespan.

## Abstract (web summary)

A multi-tissue signalling system links cold temperature to enhanced longevity and proteostasis in *Caenorhabditis elegans.*

## Main

Emerging evidence demonstrates that moderately cold temperatures delay ageing and promote health across species^1^. Lowering body temperature extends lifespan in both poikilotherms, including *C. elegans*, *Drosophila melanogaster* and various fish species, and homeotherms, including rodents^1–8^. In adult *C. elegans*, a shift from standard (20 °C) to moderately low temperature (15 °C) markedly extends lifespan^2,9–12^. In rodents, a mild decrease in body temperature by only around 0.5 °C prolongs lifespan, whereas a comparable increase shortens it^3,4^. Lower body temperature also correlates with longevity in humans^13,14^. Notably, average human body temperature has declined since the Industrial Revolution, raising the possibility that changes in body temperature may have contributed to increased longevity over recent decades^13^. Together, these findings suggest that moderate cold engages evolutionarily conserved programs that promote longevity.

Despite being recognized for more than a century^15^, the mechanisms by which moderate cold temperature promotes longevity remain largely unclear. Historically, cold-induced lifespan extension was interpreted as a consequence of passive thermodynamic effects, including reduced metabolic rate and slower biochemical reaction kinetics. However, studies in *C. elegans* revealed that cold-induced longevity is an actively regulated process^2,9–12^. For example, the temperature-sensitive TRPA-1 ion channel promotes longevity at 15–20 °C but not at warmer temperatures, indicating that low temperature is sensed and transduced into longevity mechanisms^11,12^. Moreover, cold temperature induces transcriptional programs that enhance proteostasis^2,9^.

Despite these advances, a fundamental question remains of what systemic signals coordinate cold-induced responses across tissues. Small metabolites are ideally suited for this role because they can act as rapidly diffusible regulators integrating metabolism, enzymatic activity and intertissue signalling. We therefore hypothesized that cold temperature induces metabolite-based signals that coordinate metabolic, proteostatic and longevity pathways across tissues. We further hypothesized that such metabolites could function as temperature-independent effectors capable of recapitulating the beneficial effects of cold temperature even at higher temperatures.

Here we identify BH2 as a cold-induced metabolite of which the synthesis, intertissue transport and conversion into the bioactive enzymatic cofactor BH4 constitute a multi-tissue regulatory system that promotes longevity and maintains proteostasis across tissues. Through metabolomic, genetic and physiological analyses, we find that elevated BH2 synthesis in muscle and its conversion to BH4 in distal tissues promotes tyrosine synthesis, thereby mediating key aspects of cold-induced longevity. This pathway can be leveraged to confer longevity and proteostasis benefits at normal and elevated temperatures in both *C. elegans* and human cells.

## Cold temperature increases BH2 levels

**Fig. 1:**
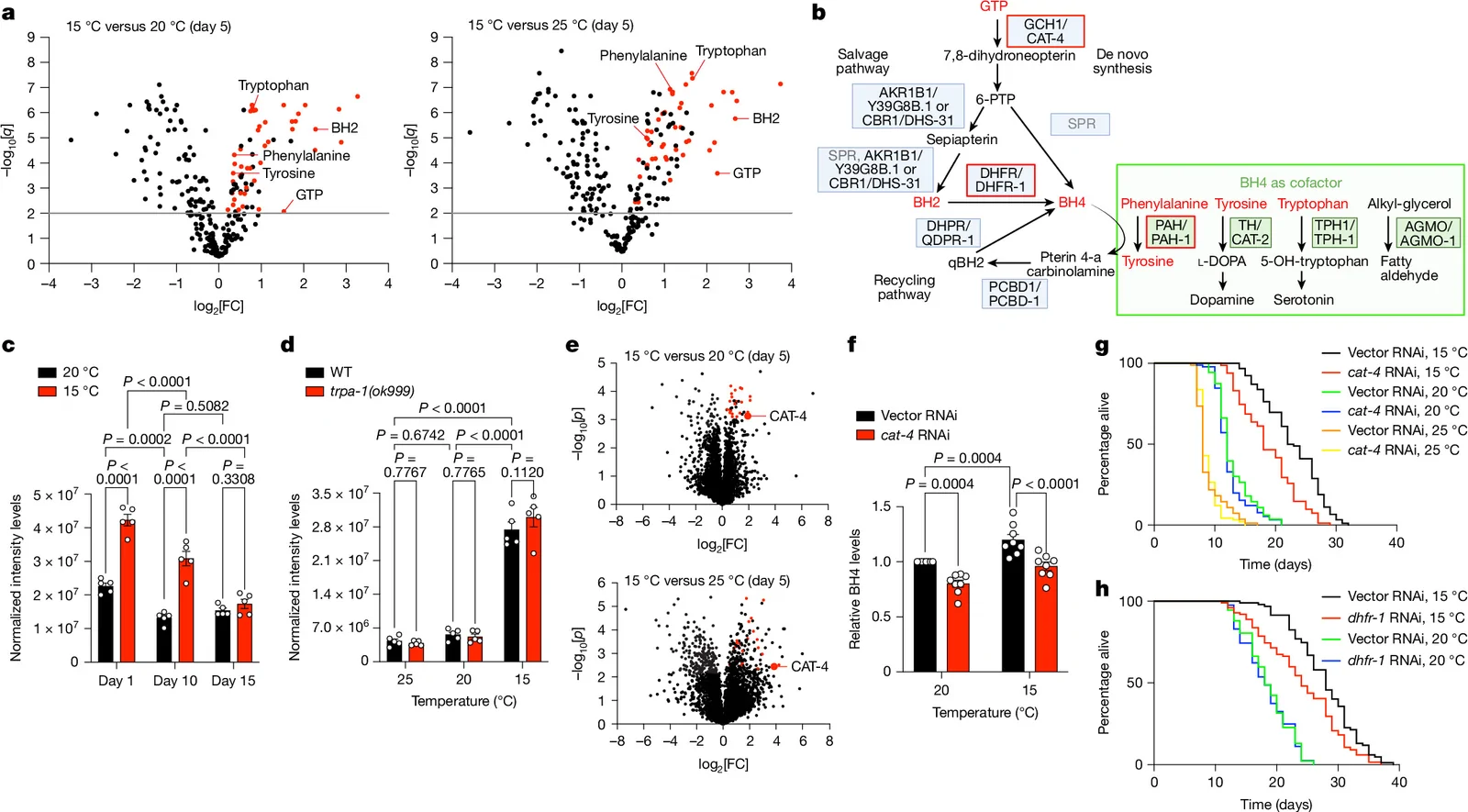
Elevated BH2 synthesis and conversion into BH4 are required for cold-induced longevity in *C. elegans*. **a**, Metabolomics of day 5 adult wild-type worms at 15 °C versus 20 °C (left) or 25 °C (right). *n* = 5 biological replicates. The volcano plots show log_2_-transformed fold change (log_2_[FC]) versus −log_10_-transformed FDR-adjusted *P* value (−log_10_[*q*]). The red dots indicate the 45 cold-induced metabolites increased at 15 °C versus both 20 °C and 25 °C in two independent experiments (FDR-adjusted *P* < 0.01; Supplementary Tables 1 and 2). **b**, Schematic of de novo, salvage and recycling BH4 synthesis pathways. Cold-induced metabolites are shown in red font; key enzymes required for cold-induced longevity are outlined by red boxes. *C. elegans* lacks sepiapterin reductase (SPR). The diagram was created using BioRender; Vilchez, D. https://BioRender.com/mno2d7j (2026). **c**, BH2 levels in adult wild-type worms across ages at 20 °C or 15 °C. Data are mean ± s.e.m. *n* = 5 biological replicates. **d**, BH2 levels in day 5 adult wild-type and *trpa-1(ok999)* mutant worms. Data are mean ± s.e.m. *n* = 5 biological replicates. **e**, Quantitative proteomics of day 5 adult wild-type worms at 15 °C versus 20 °C (above) or 25 °C (below). *n* = 3 biological replicates. The red dots indicate proteins commonly increased at 15 °C versus 20 °C and 25 °C (FDR < 0.05). **f**, BH4 levels in day 5 adult sterile *fer-15;fem-1* worms treated after development with vector or *cat-4* RNAi at 15 °C or 20 °C. Data are mean ± s.e.m., relative to 20 °C + vector RNAi. *n* = 8 independent experiments.** g**, *cat-4* RNAi after development shortens the lifespan of wild-type worms at 15 °C (*P* < 0.0001) but not at 20 °C (*P* = 0.2595) or 25 °C (*P* = 0.8993). **h**, *dhfr-1* RNAi after development shortens lifespan at 15 °C (*P* = 0.0005) but not at 20 °C (*P* = 0.7861). For **g**,**h**, *n* = 96 worms per condition; data are representative of two independent experiments. Data are mean ± s.e.m. and replicate data from independent lifespan experiments are provided in Supplementary Table 7. Statistical analysis was performed using two-sided *t*-tests with FDR correction (**a**,**e**), two-way analysis of variance (ANOVA) with Tukey’s multiple-comparison test (**c**,**d**) or Fisher’s least significant difference (LSD) test (**f**), and two-sided log-rank tests (**g**,**h**). Source data

**Extended Data Fig. 1:**
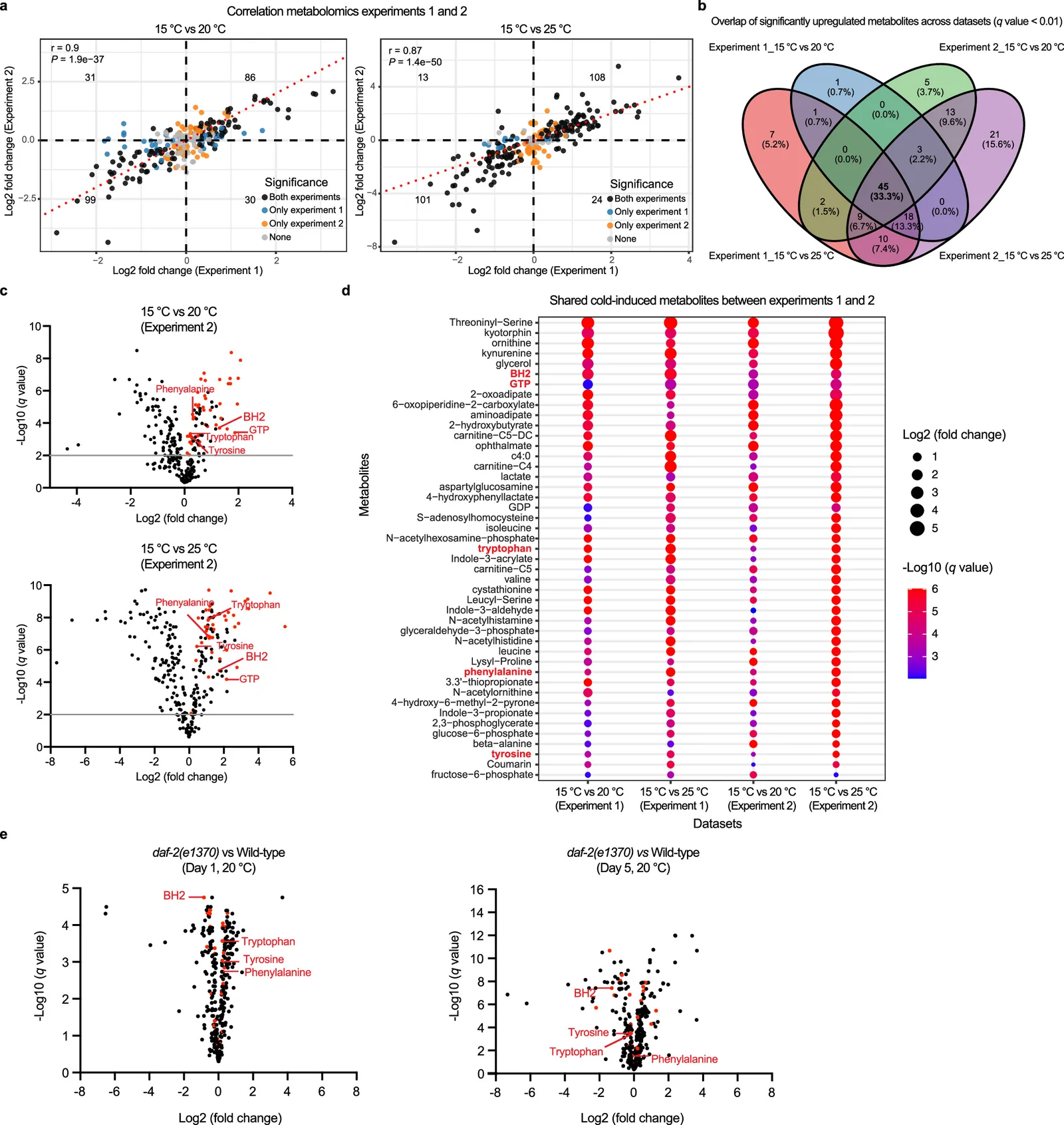
Cold temperature increases the levels of dihydrobiopterin (BH2) and related metabolites in *C. elegans*. **a**, Scatter plot comparing log2-transformed metabolite fold changes between day-5 adult wild-type worms at 15 °C and 20 °C or 25 °C in two independent experiments. Each point represents a metabolite identified in both experiments. Metabolites significantly changed in both datasets (FDR-adjusted *P* (*q* value) < 0.01) are shown in black; non-significant metabolites are shown in grey; metabolites significant only in experiment 1 (Supplementary Table 1) or 2 (Supplementary Table 2) are shown in blue or orange, respectively. Dashed lines indicate zero change in each dataset and the diagonal indicates perfect agreement. Quadrants indicate the direction of change across datasets. Two-sided Pearson correlation was calculated for metabolites significantly changed in both datasets. **b**, Venn diagram showing overlap of significantly upregulated metabolites (fold change > 1; FDR  <  0.01) across the indicated four comparisons. The core set of 45 cold-induced metabolites was consistently increased across all comparisons. **c**, Volcano plots of the second metabolomics experiment (*n* = 5 biological replicates). Red dots indicate the 45 metabolites commonly increased at 15 °C versus 20 °C and 25 °C (FDR < 0.01) and also meeting the same criteria in the first independent experiment. **d**, Dot plot of log2-transformed fold changes of the 45 metabolites significantly increased across all four comparisons: Experiment 1_15 °C vs 20 °C, Experiment 1_15 °C vs 25 °C, Experiment 2_15 °C vs 20 °C, and Experiment 2_15 °C vs 25 °C. Point size indicates fold change magnitude and colour indicates statistical significance (−log10(*q* value)). **e**, Volcano plots comparing age-matched wild-type and *daf-2(e1370)* worms at day 1 (wild-type, *n* = 6; *daf-2(e1370)*, *n* = 5 biological replicates) and day 5 (*n* = 6 biological replicates) at 20 °C (Supplementary Table 4). Red dots indicate cold-induced metabolites identified in **a**–**d**. In **c**,**e**, FDR-adjusted *P* (*q* value) were calculated using two-stage step-up Benjamini–Krieger–Yekutieli multiple t-tests.

Exposure of *C. elegans* to moderately low temperature (15 °C) after development induces a considerable extension of lifespan^2,10–12^. To assess metabolic changes associated with this effect, we raised wild-type worms at the standard temperature (20 °C) until adulthood and subsequently transferred them to 15 °C, 20 °C or 25 °C. Untargeted metabolomics analysis on day 5 of adulthood identified 64 metabolites that were significantly increased at 15 °C relative to both 20 °C and 25 °C (Fig. 1a and Supplementary Table 1). An independent experiment confirmed 45 of these metabolites, with strong concordance between datasets (Extended Data Fig. 1a–d and Supplementary Table 2).

Among the 45 reproducibly cold-induced metabolites, several were linked to BH4 synthesis and its function as an enzymatic cofactor, including GTP, BH2, tryptophan, phenylalanine and tyrosine (Fig. 1a,b). As BH2 was among the most strongly induced metabolites at cold temperature (Fig. 1a), we focused subsequent analyses on this metabolite. Cold exposure after development increased BH2 levels from day 1 of adulthood, and BH2 remained elevated at 15 °C relative to age-matched animals at 20 °C until at least day 10 (Fig. 1c and Supplementary Table 3). BH2 induction was not a general consequence of longevity, as reduced insulin signalling, a distinct longevity pathway, did not increase BH2 levels at 20 °C (Extended Data Fig. 1e and Supplementary Table 4).

Loss of the temperature-sensitive *trpa-1* channel shortens lifespan under both standard and cold temperatures^11^. However, worms still exhibit substantially extended lifespan at 15 °C compared with at 20 °C, indicating that cold-induced longevity is not solely mediated by TRPA-1-dependent pathways. Loss of *trpa-1* did not reduce BH2 levels at cold temperature (Fig. 1d and Supplementary Table 1), demonstrating that elevated BH2 synthesis is induced independently of canonical TRPA-1 temperature sensing.

**Extended Data Fig. 2:**
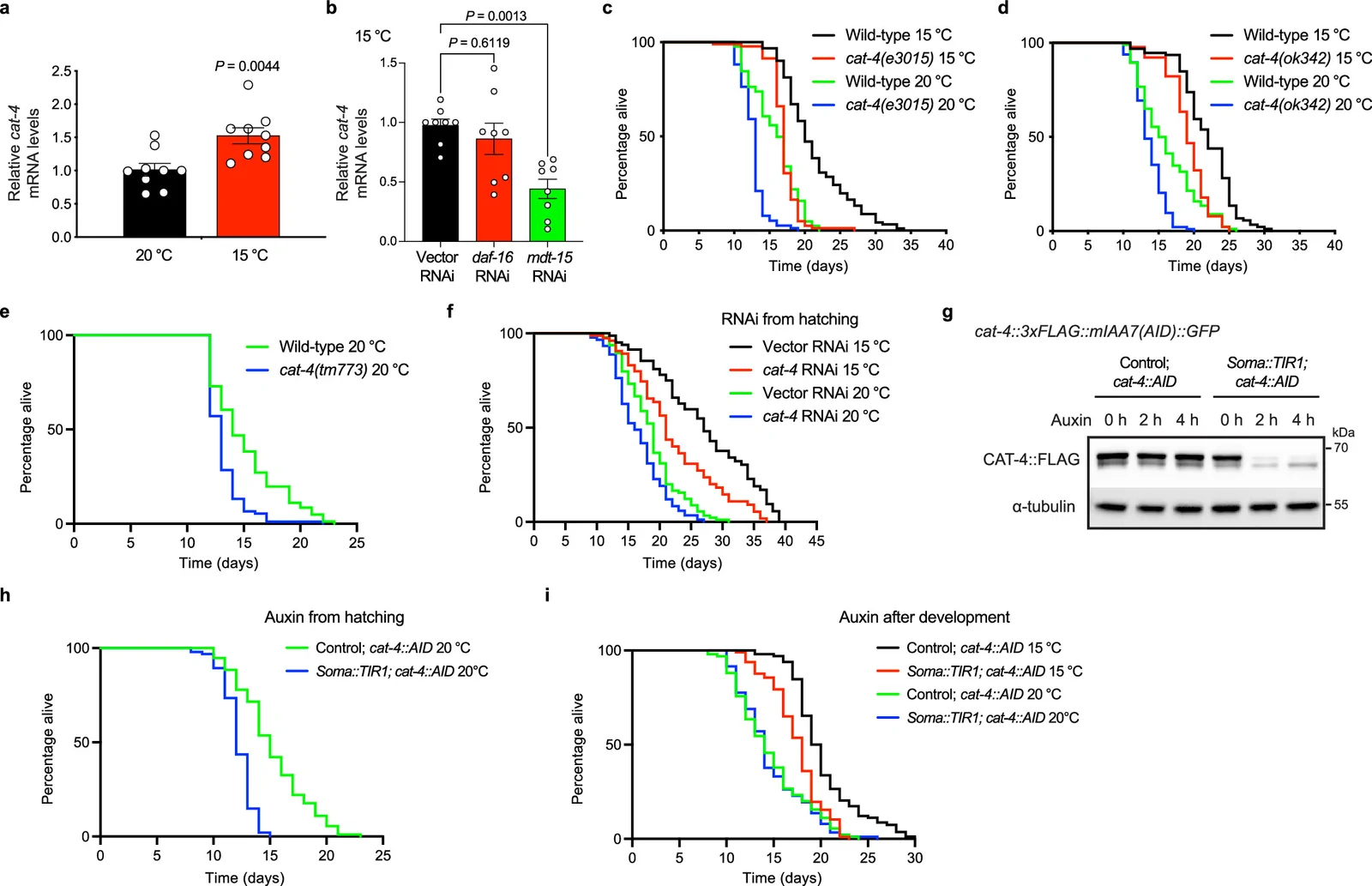
Degradation of CAT-4 after development specifically shortens longevity at 15 °C. **a**, *cat-4* mRNA levels comparing day-5 adult wild-type worms at 20 °C and 15 °C (relative to 20 °C; mean ± s.e.m.; *n* = 9 biological replicates from two independent experiments; *P* = 0.0044; two-sided t-test for unpaired samples). **b**, Knockdown of *mdt-15* (*P* = 0.0013), but not *daf-16* (*P* = 0.6119), reduces *cat-4* expression in day-5 adult worms at 15 °C (relative to vector RNAi; mean ± s.e.m.; *n* = 8 biological replicates from four independent experiments; one-way ANOVA with Dunnett’s multiple comparison test). **c**, *cat-4(e3015)* mutant worms are short-lived at 15 °C (*P* < 0.0001) and 20 °C (*P* < 0.0001). **d**, *cat-4(ok342)* mutant worms are short-lived at 15 °C (*P* < 0.0001) and 20 °C (*P* < 0.0001). **e**, *cat-4(tm773)* mutant worms are short-lived at 20 °C (*P* < 0.0001). **f**, Knockdown of *cat-4* from egg hatching shortens the lifespan of wild-type worms at 15 °C (*P* = 0.0001), and to a lesser extent also at 20 °C (*P* = 0.0027). **g**, Western blot analysis of auxin-inducible degron (AID)-mediated degradation of CAT-4 protein at 20 °C. Ubiquitous somatic degradation of CAT-4 was induced by expression of TIR1 under the *eft-3* promoter. Worms lacking TIR1 served as controls. Animals were transferred to auxin-containing plates after development and maintained on auxin for the indicated hours before analysis. CAT-4 was detected using an anti-FLAG antibody. α-tubulin is the loading control. **h**, AID-mediated degradation of CAT-4 from egg hatching shortens lifespan at 20 °C (*P* < 0.0001). **i**, AID-mediated degradation of CAT-4 after development specifically shortens longevity at 15 °C (*P* < 0.0001), but not 20 °C (*P* = 0.8656). In all the lifespan experiments (**c**–**f**,**h**,**i**), statistical comparisons were made by two-sided log-rank test, *n* = 96 worms per condition. Lifespan data are representative of two independent experiments. Mean ± s.e.m. and replicate data of lifespan experiments are provided in Supplementary Table 7. For gel source data, see Supplementary Fig. 1. Source data

We next examined whether BH2 reflects a regulated pathway rather than a passive metabolic consequence of reduced temperature. Quantitative proteomics identified GTP cyclohydrolase I (GCH1 in humans, CAT-4 in *C. elegans*), the first and rate-limiting enzyme in BH2 synthesis^16,17^, as one of the most strongly induced proteins at 15 °C (Fig. 1e and Supplementary Table 5). Cold temperature also increased *cat-4* mRNA amounts, indicating that CAT-4 induction occurs, at least in part, at the transcriptional level (Extended Data Fig. 2a). MED15 (human)/MDT-15 (*C. elegans*), a key transcriptional regulator of metabolic responses, is required for cold-induced longevity^9^. Importantly, knockdown of *mdt-15* reduced *cat-4* transcript levels at 15 °C (Extended Data Fig. 2b), indicating that cold-induced CAT-4 expression is part of a regulated transcriptional program.

## BH2 is required for cold-induced longevity

Although BH2 is an inactive metabolite, it functions as a key intermediate in the synthesis and intertissue transport of its reduced, bioactive form, BH4^16,17^. As BH4 is chemically unstable and readily oxidized, the more-stable BH2 form serves as a major organismal reservoir of this bioactive metabolite^16,17^. Consequently, *cat-4-*mutant worms are severely depleted of BH4^18^. Consistent with the essential role of BH4 as a cofactor for multiple enzymes, these worms exhibit increased fragility compared with wild-type worms^18^. Accordingly, *cat-4*-mutant worms were short lived under both cold and standard temperatures (Extended Data Fig. 2c–e). Knockdown of *cat-4* from early development also shortened lifespan at both 15 °C and 20 °C, although the effect was more pronounced under cold temperatures (Extended Data Fig. 2f).

As cold temperature during adulthood was sufficient to induce CAT-4 expression, BH2 synthesis and lifespan extension, we next examined the effects of *cat-4* knockdown after development. As BH4 could not be detected by metabolomics because of its chemical instability, we quantified BH4 using an enzyme-linked immunosorbent assay (ELISA). Cold temperature induced a significant but modest increase in BH4 levels (Fig. 1f), consistent with BH2 serving as the major intermediate reservoir for this metabolite^16,17^. Knockdown of *cat-4* after development decreased BH4 levels under both standard and cold temperatures, although this reduction was moderate (Fig. 1f). Importantly, *cat-4* RNA interference (RNAi) decreased BH4 at cold temperature only to levels comparable to those observed at standard temperature without knockdown (Fig. 1f). Thus, BH4 levels remained within a physiological range, enabling us to determine whether elevated CAT-4 activity during adulthood is required for cold-induced longevity. Notably, knockdown of *cat-4* after development diminished the lifespan extension induced by cold temperature, but had no significant effect on lifespan at 20 °C or 25 °C (Fig. 1g).

To further dissect the adult-specific requirement for elevated CAT-4 in cold-induced longevity, we generated an auxin-inducible degron (AID) strain enabling conditional degradation of CAT-4 protein in somatic tissues (Extended Data Fig. 2g). CAT-4 degradation from early development substantially reduced lifespan at 20 °C (Extended Data Fig. 2h). By contrast, degradation of CAT-4 after development did not affect lifespan at 20 °C, but specifically shortened longevity at 15 °C (Extended Data Fig. 2i). These results further indicate that CAT-4 activity is essential during development for adult viability, whereas increased CAT-4 activity during adulthood specifically contributes to cold-induced longevity.

**Extended Data Fig. 3:**
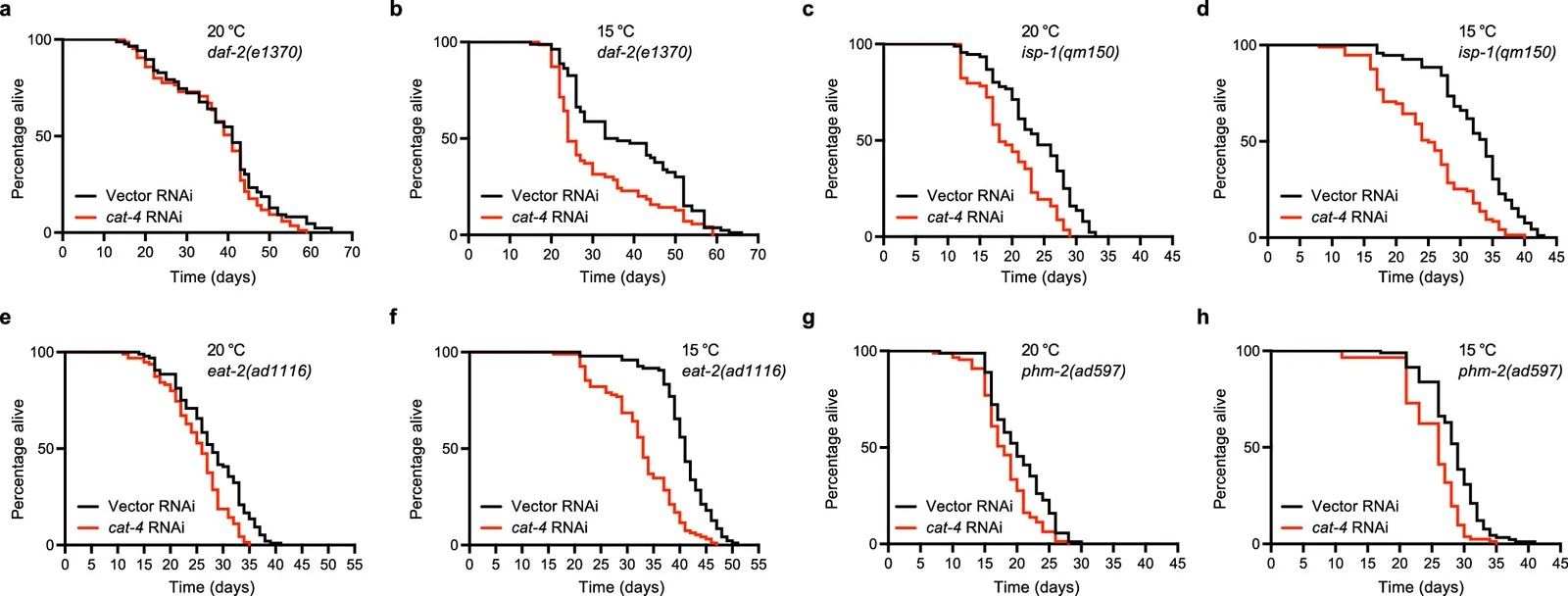
Knockdown of *cat-4* shortens longevity in mutant worms with impaired mitochondrial electron transport or reduced food intake at standard temperature. **a**, Knockdown of *cat-4* after development does not decrease the lifespan of *daf-2(e1370)* mutant worms at 20 °C (*P* = 0.1715). **b**, Knockdown of *cat-4* after development shortens the lifespan of *daf-2(e1370)* mutant worms at 15 °C (*P* = 0.0005). **c**, Knockdown of *cat-4* after development decreases lifespan of *isp-1(qm150)* mutant worms at 20 °C (*P* < 0.0001). **d**, Knockdown of *cat-4* after development decreases lifespan of *isp-1(qm150)* mutant worms at 15 °C (*P* < 0.0001). **e**, Knockdown of *cat-4* after development decreases lifespan of *eat-2(ad1116)* mutant worms at 20 °C (*P* < 0.0001). **f**, Knockdown of *cat-4* after development decreases lifespan of *eat-2(ad1116)* mutant worms at 15 °C (*P* < 0.0001). **g**, Knockdown of *cat-4* after development decreases lifespan of *phm-2(ad597)* mutant worms at 20 °C (*P* = 0.0011). **h**, Knockdown of *cat-4* after development decreases lifespan of *phm-2(ad597)* mutant worms at 15 °C (*P* < 0.0001). Statistical comparisons were made by two-sided log-rank test, *n* = 96 worms per condition. All the lifespan data are representative of two independent experiments. Mean ± s.e.m. and replicate data of lifespan experiments are provided in Supplementary Table 7.

Prompted by these findings, we next examined whether CAT-4 activity also contributes to lifespan extension in other longevity paradigms. Knockdown of *cat-4* in mutant worms with reduced insulin signalling did not affect their extended lifespan at standard temperature, but only shortened lifespan at 15 °C (Extended Data Fig. 3a,b). By contrast, knockdown of *cat-4* shortened lifespan in long-lived worms with reduced mitochondrial electron-transport chain activity or reduced food intake at both cold and standard temperatures (Extended Data Fig. 3c–h), suggesting that CAT-4 activity contributes to other longevity paradigms beyond cold temperature.

**Extended Data Fig. 4:**
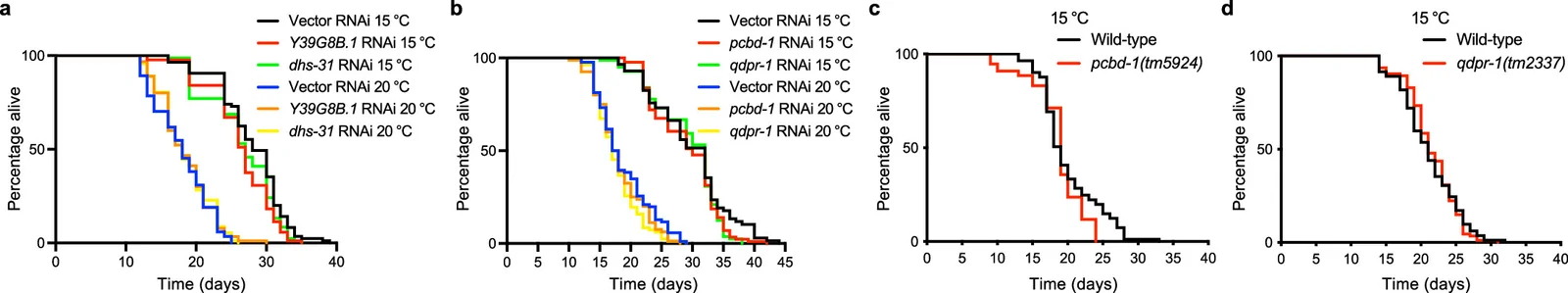
The BH4 recycling pathway is dispensable for cold-induced longevity. **a**, RNAi against aldose reductase *Y39G8B.1* during adulthood shortens the lifespan of wild-type worms at 15 °C (*P* = 0.0069), but not at 20 °C (*P* = 0.5490). Although knockdown of *dhs-31* also slightly decreases cold-induced longevity, this effect was not statistically significant (*P* = 0.0634). **b**, Knockdown of enzymes required for BH4 recycling does not shorten lifespan at 15 °C (*pcbd-1* RNAi: *P* = 0.1569; *qdpr-1* RNAi: *P* = 0.1675). Similarly, their knockdown does not affect lifespan at 20 °C (*pcbd-1* RNAi: *P* = 0.2446; *qdpr-1* RNAi: *P* = 0.0564). RNAi treatment was initiated after development. **c**, *pcbd-1(tm5924)* mutant worms are not short-lived at 15 °C (*P* = 0.1661). **d**, *qdpr-1(tm2337)* mutant worms are not short-lived at 15 °C (*P* = 0.9642). Statistical comparisons were made by two-sided log-rank test, *n* = 96 worms per condition. Mean ± s.e.m., statistical analyses and replicate data from independent lifespan experiments are provided in Supplementary Table 7.

Downstream of activity of GCH1/CAT-4, bioactive BH4 can be generated through three pathways: the de novo, salvage and recycling pathways^16,17^ (Fig. 1b). In the de novo pathway, a GCH1/CAT-4-dependent intermediate is converted to BH4 by sepiapterin reductase. In the salvage pathway, this intermediate is instead converted to BH2 by either aldose reductase (AKR1B1 (human), Y39G8B.1 (*C. elegans*)) or carbonyl reductase 1 (CBR1 (human), DHS-31 (*C. elegans*))^16,19^. This pathway operates alongside de novo BH4 synthesis, but is particularly important in mammalian tissues with low sepiapterin reductase activity and in organisms such as *C. elegans*, which lack a sepiapterin reductase orthologue^16,18^. Knockdown of the BH2-producing enzyme *Y39G8B.1* reduced lifespan specifically at 15 °C, whereas loss of *dhs-31* produced a smaller, non-significant effect (Extended Data Fig. 4a). In the salvage pathway, BH2 is further converted into bioactive BH4 by dihydrofolate reductase (DHFR (human), DHFR-1 (*C. elegans*)). Importantly, knockdown of *dhfr-1* after development decreased longevity at cold temperature but did not shorten lifespan at standard temperature (Fig. 1h). These results indicate that, downstream of CAT-4 activity, the conversion of BH2 into bioactive BH4 by DHFR-1 contributes to cold-induced longevity.

A recycling mechanism regenerates BH4 after its use as an enzymatic cofactor^16,18^ (Fig. 1b). In *C. elegans*, the recycling pathway is not required to maintain steady-state BH4 levels and becomes rate-limiting only under conditions of low BH4 synthesis, such as in reduction-of-function *cat-4* mutants^18^. Subsequently, knockdown of recycling enzymes did not shorten the lifespan of wild-type worms at cold temperature (Extended Data Fig. 4b). Likewise, mutant worms defective in BH4 recycling were not short lived at 15 °C compared with wild-type worms (Extended Data Fig. 4c,d), indicating that this pathway is dispensable for cold-induced longevity. Together, our findings support a central role of CAT-4 activity and the salvage pathway in elevated BH4 synthesis for cold-induced longevity.

## Muscle BH2 promotes systemic longevity

**Fig. 2:**
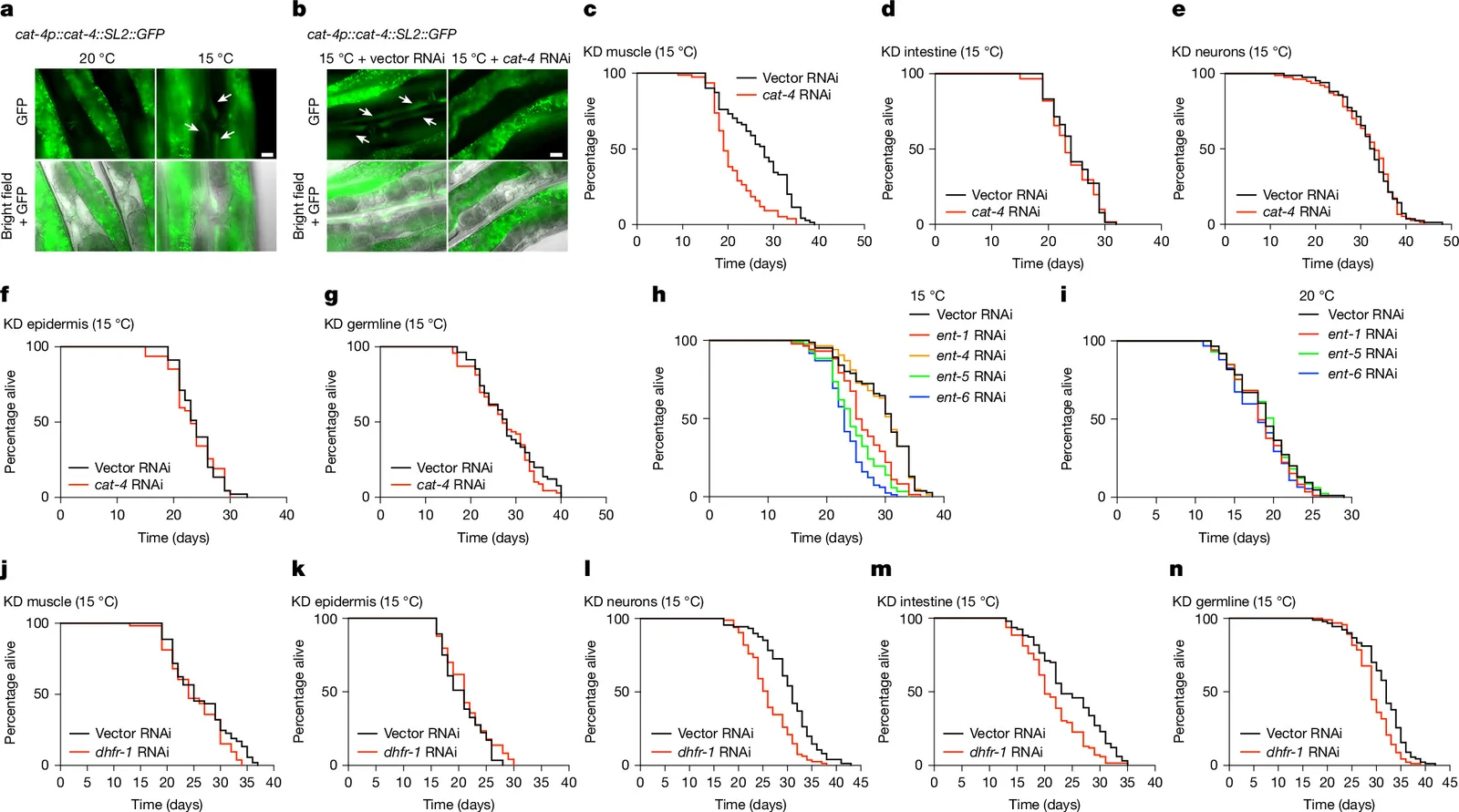
Elevated BH2 production in muscle cells at cold temperature extends longevity in a cell non-autonomous manner. **a**, Representative images of day 5 adult worms expressing *cat-4p::cat-4::SL2::GFP* at the indicated temperatures. Cold temperature induces *cat-4* expression in muscle cells. **b**, *cat-4* RNAi after development abolishes cold-induced CAT-4 upregulation in muscle. In **a**,**b**, the arrows indicate CAT-4 in muscle. Scale bars, 20 µm (**a**,**b**). Images are representative of three independent experiments. **c**–**g**, Tissue-specific *cat-4* knockdown (KD) after development. Muscle-specific knockdown shortens cold-induced longevity (**c**; *P* < 0.0001), whereas intestinal (**d**; *P* = 0.5374), neuronal (**e**; *P* = 0.8708), epidermal (**f**; *P* = 0.8339) or germline (**g**; *P* = 0.2604) knockdown does not. **h**, Single knockdown of *ent-1* (*P* < 0.0001), *ent-5* (*P* < 0.0001) or *ent-6* (*P* < 0.0001), but not *ent-4* (*P* = 0.9091), shortens lifespan at 15 °C. **i**, Knockdown of *ent-1* (*P* = 0.2209), *ent-5* (*P* = 0.9840) or *ent-6* (*P* = 0.1278) does not shorten lifespan at 20 °C. **j**–**n**, Tissue-specific *dhfr-1* RNAi after development. Muscle (**j**; *P* = 0.1076) or epidermal (**k**; *P* = 0.1830) knockdown does not affect cold-induced longevity, whereas neuronal (**l**; *P* < 0.0001), intestinal (**m**; *P* = 0.0001) or germline (**n**; *P* = 0.0001) knockdown reduces lifespan at 15 °C. In **c**–**n**, *n* = 96 worms per condition; data are representative of at least two independent experiments. Statistical comparisons were performed using two-sided log-rank tests. Data are mean ± s.e.m. Details of statistical analyses, and replicate data of lifespan experiments are provided in Supplementary Table 7.

**Extended Data Fig. 5:**
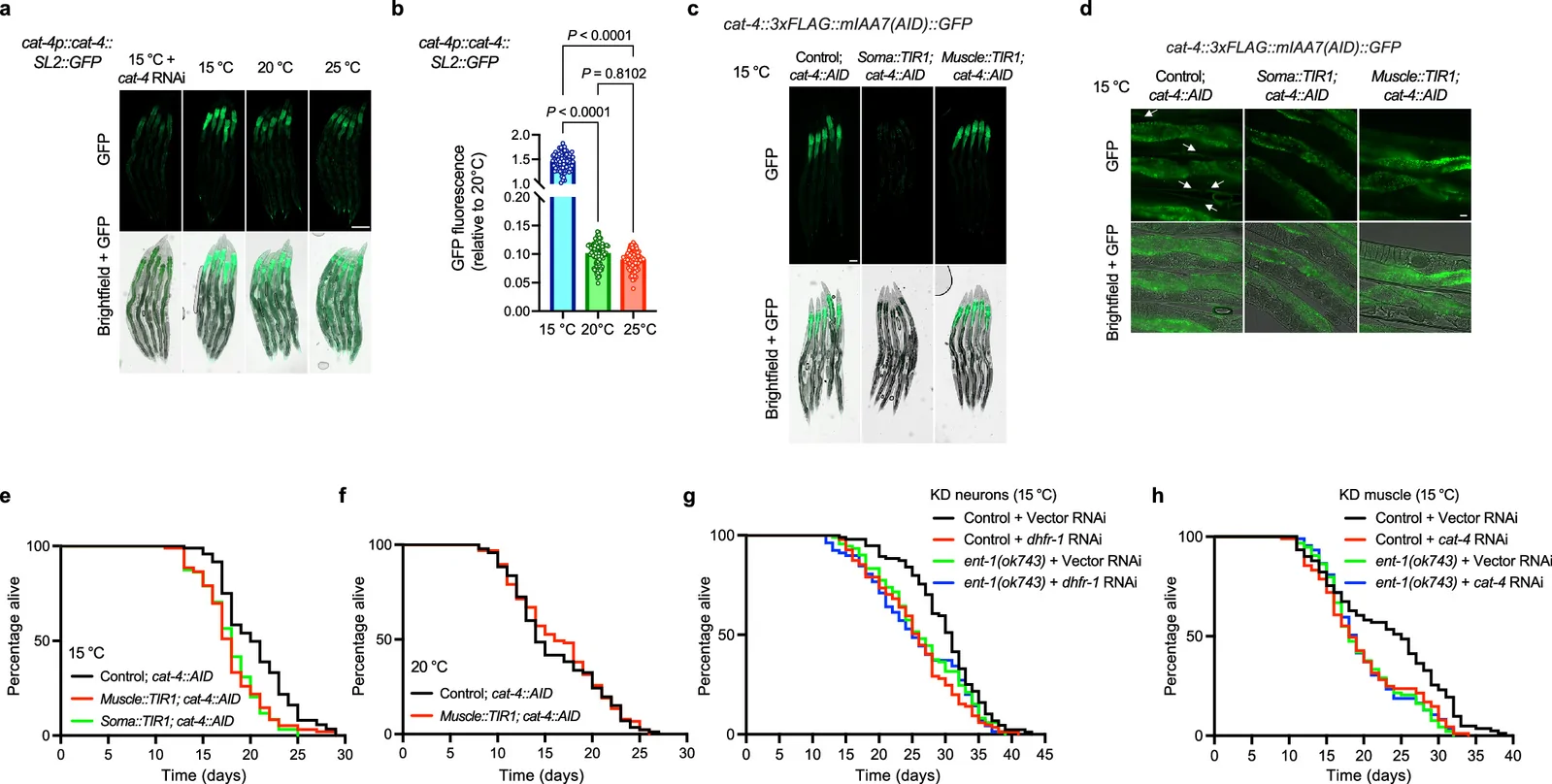
Muscle-specific AID-mediated depletion of CAT-4 after development is sufficient to reduce cold-induced longevity. **a**, Representative images of *cat-4p::cat-4::SL2::GFP* worms at the indicated temperatures. Scale bar, 200 µm. Images are representative of three independent experiments. **b**, Quantification of GFP fluorescence in the anterior intestine of day-5 adult *cat-4p::cat-4::SL2::GFP* worms (mean ± s.e.m. relative to 20 °C; 15 °C, *n* = 83; 20 °C, *n* = 85; 25 °C, *n* = 83 worms from three independent experiments). Statistical comparisons: one-way ANOVA with Tukey’s multiple-comparison test. **c**,**d**, Representative whole-worm (**c**) and muscle-region (**d**) images of CAT-4::AID worms lacking TIR1 expression or expressing TIR1 ubiquitously in somatic cells or specifically in muscle at 15 °C. Animals were transferred to auxin-containing plates after development and maintained on auxin until day 5 of adulthood. Arrows indicate CAT-4 in muscle tissue. Scale bars, 100 µm (**c**) and 20 µm (**d**). Images are representative of three independent experiments. **e**, Muscle-specific degradation of CAT-4 after development is sufficient to fully phenocopy the longevity-shortening effects of ubiquitous somatic CAT-4 degradation at 15 °C. Lifespan was significantly reduced in both *Soma::TIR1; cat-4::AID* (*P* < 0.0001) and *Muscle::TIR1; cat-4::AID* worms (*P* < 0.0001) compared with control animals, whereas no difference was observed between ubiquitous somatic and muscle-specific CAT-4 degradation (*P* = 0.9508). **f**, Muscle-specific CAT-4 degradation after development does not shorten lifespan at 20 °C (*P* = 0.7330). **g**, *ent-1(ok743)* mutant worms are short-lived at 15 °C (*P* = 0.0098). Neuron-specific knockdown (KD) of *dhfr-1* decreases cold-induced longevity in control worms (*P* < 0.0001), but does not further shorten the lifespan of *ent-1* mutant animals (*P* = 0.5491). **h**, *ent-1(ok743)* worms are short-lived at 15 °C (*P* < 0.0001). Muscle-specific knockdown of *cat-4* decreases cold-induced longevity in control worms (*P* = 0.0007), but does not further shorten the lifespan of *ent-1* mutant animals (*P* = 0.6782). In lifespan assays, statistical comparisons were made by two-sided log-rank test, *n*  =  96 worms per condition. Mean ± s.e.m., statistical analyses and replicate data are provided in Supplementary Table 7. Source data

To determine the tissues in which CAT-4 is expressed, we generated a GFP-based fluorescent reporter driven by the endogenous *cat-4* promoter. Knockdown of *cat-4* reduced the GFP signal, validating the reporter (Extended Data Fig. 5a). At standard temperature, CAT-4 was mostly expressed in the anterior intestine, and cold exposure further increased CAT-4 levels in this region (Extended Data Fig. 5a,b). In contrast to the intestine, CAT-4 was barely detectable in muscle at 20 °C (Fig. 2a). However, cold temperature triggered robust expression of *cat-4* in the muscle, an effect that was abolished by *cat-4* RNAi treatment (Fig. 2a,b). To assess whether elevated BH2 synthesis in these tissues promotes organismal longevity at cold temperature, we performed tissue-specific knockdown experiments. Notably, knockdown of *cat-4* in the muscle alone reduced cold-induced longevity, whereas knockdown in the intestine had no effect on lifespan (Fig. 2c,d). Likewise, knockdown of *cat-4* in neurons, epidermis or germline did not shorten lifespan at 15 °C (Fig. 2e–g).

To further investigate the link between cold-induced CAT-4 expression in muscle and longevity, we generated worms in which auxin-inducible degradation of CAT-4 was restricted to muscle. We confirmed that auxin treatment specifically depleted CAT-4 in the muscle of these animals, whereas ubiquitous somatic degradation reduced CAT-4 levels in both muscle and intestine (Extended Data Fig. 5c,d). Notably, muscle-specific degradation of CAT-4 after development was sufficient to fully phenocopy the longevity-shortening effects of ubiquitous somatic CAT-4 degradation at 15 °C (Extended Data Fig. 5e). This effect was specific to cold temperature, as muscle-specific CAT-4 degradation did not affect lifespan at standard temperature (Extended Data Fig. 5f). Together, these results identify muscle as a critical tissue in which elevated BH2 synthesis promotes cold-induced longevity.

BH4 is essential for the function of amino acid hydroxylases (TPH1 (human), TPH-1 (*C. elegans*); TH (human), CAT-2 (*C. elegans*); PAH (human), PAH-1 (*C. elegans*) and alkylglycerol monooxygenase (AGMO (human), AGMO-1 (*C. elegans*)). However, these enzymes are not expressed in muscle cells^18^, raising the possibility that BH4 acts in a cell non-autonomous manner. BH2 is the principal transport intermediate for BH4 distribution between tissues, enabling the rapid generation and utilization of bioactive BH4 in distal tissues^16,17^. We therefore hypothesized that cold temperature increases BH2 synthesis in muscle cells, which is then released and transported to distal tissues, where it is converted into BH4 to activate its target enzymes. In humans, the uptake of BH2 is mediated by the transmembrane nucleoside transporters ENT1 (SLC29A1) and ENT2 (SLC29A2)^16^. *C. elegans* expresses seven orthologues of human *ENT1* and *ENT2* (*ent-1* to *ent-7*). We found that single knockdown of different orthologues, such as *ent-1*, *ent-5* and *ent-6*, specifically shortens lifespan at 15 °C but not 20 °C (Fig. 2h,i).

Once BH2 is taken up by distal cells, it can be converted into bioactive BH4 through the salvage pathway by the DHFR-1 enzyme^16,20^. Consequently, BH4 redistribution efficiency depends on DHFR-1 activity, which regulates local BH4 availability. While BH2 synthesis through CAT-4 activity in muscle cells is required for cold-induced longevity (Fig. 2c), we found that muscle-specific knockdown of *dhfr-1* does not shorten lifespan at 15 °C (Fig. 2j). Similarly, *dhfr-1* knockdown in the epidermis had no effect on longevity at 15 °C (Fig. 2k). By contrast, tissue-specific knockdown of *dhfr-1* in neurons, intestine and germline reduced cold-induced longevity (Fig. 2l–n). Moreover, neither neuron-specific knockdown of *dhfr-1* nor muscle-specific knockdown of *cat-4* further reduced the shortened lifespan of *ent-1* mutant animals at cold temperature (Extended Data Fig. 5g,h). These results support the notion that cold temperature induces BH2 synthesis in muscle cells, after which, BH2 is released and converted to bioactive BH4 in distal tissues such as neurons, intestine and germline, where it promotes cold-induced longevity.

**Extended Data Fig. 6:**
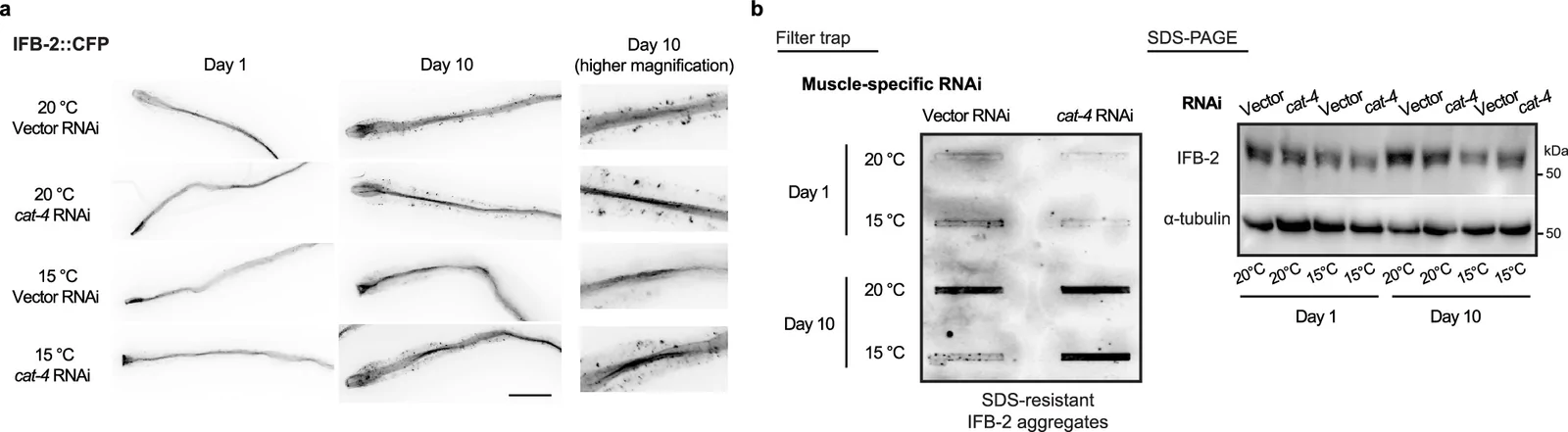
Cold temperature suppresses age-dependent IFB-2 aggregation in the intestine, an effect that requires CAT-4 activity in the muscle. **a**, Representative images of day-1 and day-10 adult worms expressing IFB-2::CFP at standard (20 °C) and cold (15 °C) temperatures. Where indicated, worms were treated with *cat-4* RNAi after development. Scale bar, 100 µm. Images are representative of three independent experiments. **b**, Filter-trap analysis of endogenous IFB-2 aggregation (detected by anti-IFB-2 antibody) in adult wild-type *C. elegans* at the indicated ages and temperatures. When indicated, *cat-4* was specifically knocked down in the muscle. Right: SDS–polyacrylamide gel electrophoresis (PAGE) with antibodies to IFB-2 and α-tubulin. Images are representative of three independent experiments. For gel source data, see Supplementary Fig. 1.

To further examine the cell non-autonomous effects of BH2, we focused on the intestine, a tissue that progressively loses structural integrity during ageing^21,22^. To this end, we assessed the intermediate filament IFB-2 because this protein is expressed exclusively in the intestine and undergoes age-related changes that can be readily monitored^22^. In young animals, IFB-2 is confined to the apical cytoplasm of intestinal cells. With age, IFB-2 levels increase and the protein becomes mislocalized throughout the cytoplasm, where it forms aggregates that contribute to intestinal decline^22^. We found that cold temperature prevents IFB-2 mislocalization and aggregation during ageing (Extended Data Fig. 6a). However, loss of *cat-4* abolished these protective effects (Extended Data Fig. 6a). Notably, muscle-specific knockdown of *cat-4* was sufficient to block the cold-induced suppression of intestinal IFB-2 aggregation (Extended Data Fig. 6b). Collectively, these findings indicate that cold-induced BH2 synthesis in muscle exerts cell non-autonomous effects, such as preserving intestinal integrity during ageing.

## CAT-4 overexpression extends lifespan

**Extended Data Fig. 7:**
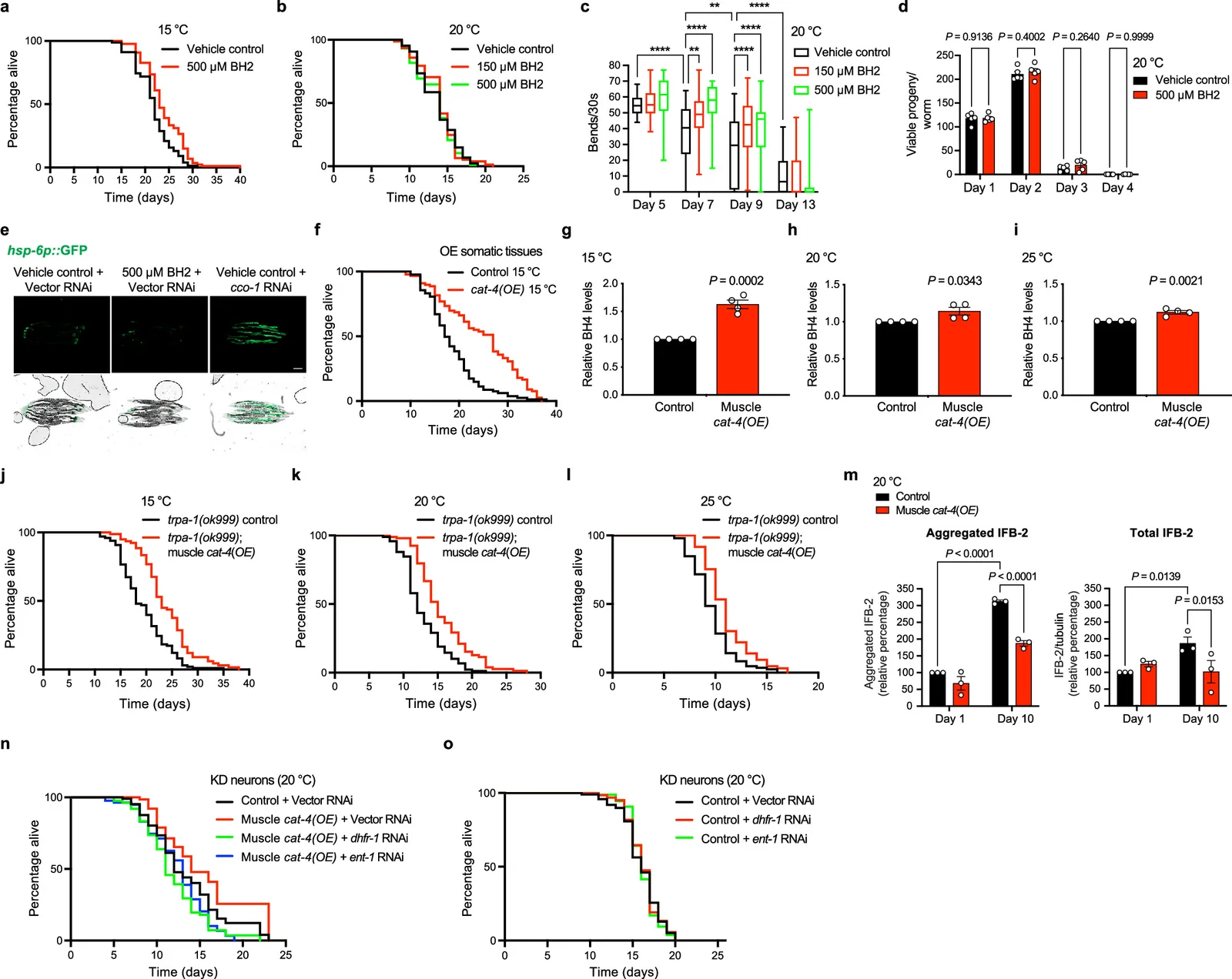
Muscle-specific *cat-4* overexpression extends lifespan independently of temperature and the TRPA-1 channel. **a**, BH2 supplementation after development extends longevity at 15 °C (*P* = 0.0006). Vehicle control: DMSO. **b**, Lifespan with BH2 supplementation at 20 °C (150 µM: *P* = 0.7502; 500 µM: *P* = 0.6048). **c**, Thrashing movements of wild-type worms at the indicated ages at 20 °C (*n* = 50 worms per condition from two independent experiments). Box plots show 25th–75th percentiles, median and minimum–maximum values. Statistics: two-way ANOVA with Šidák’s multiple-comparison test, **P < 0.01, ****P < 0.0001. **d**, Number of viable progeny per worm on BH2 supplementation (mean ± s.e.m.; *n* = 5 biological replicates; two-way ANOVA with Tukey’s multiple-comparison test). **e**, *hsp-6p*::*GFP* expression in worms treated with 500 µM BH2 or *cco-1* RNAi. RNAi treatment was initiated from hatching. Scale bar, 200 μm. Representative of two independent experiments. **f**, Ubiquitous somatic *cat-4* overexpression (OE) extends lifespan at 15 °C (*P* < 0.0001). **g–i**, BH4 levels in muscle-specific *cat-4*-overexpressing worms at 15 °C (**g**), 20 °C (**h**) and 25 °C (**i**). Mean ± s.e.m. relative to control worms; *n* = 4 independent experiments; two-sided *t*-test for unpaired samples. **j**–**l**, Muscle-specific *cat-4* overexpression extends lifespan in *trpa-1(ok999)* mutant worms at 15 °C (**j**, *P* < 0.0001), 20 °C (**k**, *P* < 0.0001), and 25 °C (**l**, *P* < 0.0001). **m**, Quantification of aggregated and total IFB-2 from Fig. 3c (mean ± s.e.m. relative to day-1 control; *n* = 3 independent experiments; two-way ANOVA with Fisher’s LSD test). **n**, Muscle-specific *cat-4* overexpression extends lifespan at 20 °C (*P* = 0.0237), an effect reduced by neuronal-specific knockdown (KD) of *dhfr-1* (*P* < 0.0001) or *ent-1* (*P* = 0.0004) after development. **o**, Neuronal-specific knockdown of *dhfr-1* (*P* = 0.7964) or *ent-1* (*P* = 0.9892) does not shorten lifespan in control worms at 20 °C. For lifespan experiments: *n* = 96 worms per condition; two-sided log-rank test; representative of at least two independent experiments. Mean ± s.e.m. and replicate data of lifespans are provided in Supplementary Table 7. Source data

BH2 supplementation is often more effective at increasing BH4 levels than direct BH4 administration^16,20^. However, *C. elegans* exhibits a limited capacity for biopterin uptake, including BH2, and exogenous supplementation results in only a modest increase in biopterin levels^18^. Despite this limitation, BH2 supplementation in adult worms led to a slight but significant extension of cold-induced longevity (Extended Data Fig. 7a). Although BH2 supplementation did not extend lifespan at standard temperature, it delayed the age-related decline in motility, a key indicator of healthspan (Extended Data Fig. 7b,c). Although BH2-treated worms did not display overt signs of sickness, we further assessed the possibility that these beneficial effects arise from a hormetic stress response, such as that induced by mitochondrial dysfunction^23,24^. However, BH2 supplementation did not reduce brood size and did not induce the mitochondrial unfolded protein response (Extended Data Fig. 7d,e), two commonly used indicators of physiological stress.

**Fig. 3:**
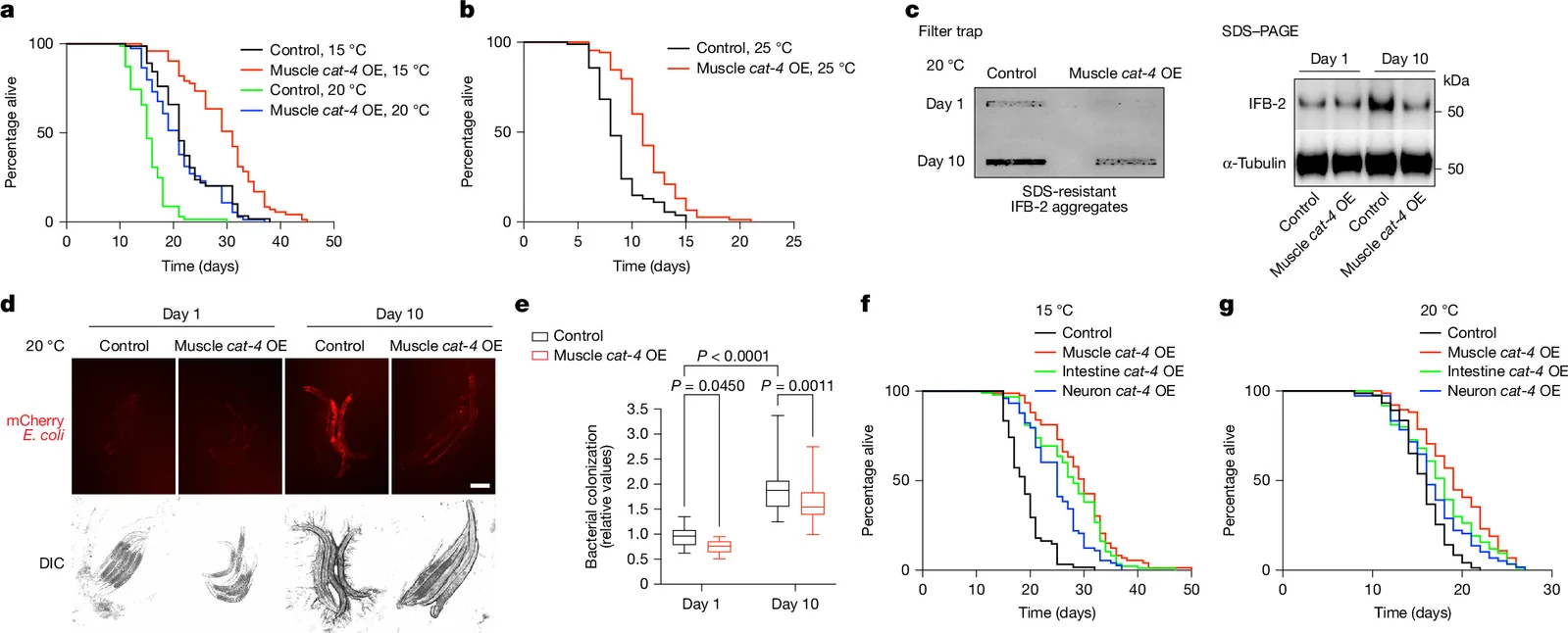
Overexpression of CAT-4 in muscle cells extends longevity at cold and higher temperatures. **a**,**b**, Muscle-specific *cat-4* overexpression (OE) extends lifespan at 15 °C and 20 °C (**a**; *P* < 0.0001) and at 25 °C (**b**; *P* < 0.0001). **c**, Filter-trap analysis of intestinal IFB-2 aggregation (detected by anti-IFB-2 antibody) in control and muscle-specific *cat-4*-overexpressing worms at 20 °C on days 1 and 10 of adulthood. Right, SDS–PAGE and immunoblotting of IFB-2 and the α-tubulin loading control. Images are representative of three independent experiments; quantification is shown in Extended Data Fig. 7m. **d**, Representative images of intestinal colonization by mCherry-expressing *Escherichia* *coli* in control and muscle-specific *cat-4*-overexpressing worms on days 1 and 10. Scale bar, 200 µm. Representative of three independent experiments. DIC, differential interference contrast. **e**, Quantification of intestinal bacterial colonization at 20 °C. The box plots represent the 25th–75th percentiles, the lines depict the median and the whiskers show the minimum–maximum values. Day 1: control, *n* = 33 worms; muscle *cat-4(OE)*, *n* = 24; day 10: control, *n* = 23; muscle *cat-4(OE)*, *n* = 27, from three independent experiments. Statistical analysis was performed using two-way ANOVA with Fisher’s LSD test. **f**,**g**, In addition to muscle-specific overexpression, intestinal and neuronal *cat-4* overexpression also extends longevity at cold (15 °C; **f**) and standard (20 °C; **g**) temperature. *P* < 0.0001 for muscle, intestinal and neuronal overexpression at 15 °C; *P* < 0.0001 for muscle and intestinal overexpression at 20 °C; and *P* = 0.0121 for neuronal overexpression at 20 °C. In **a**,**b**,**f**,**g**, *n* = 96 worms per condition; data are representative of at least two independent experiments. Statistical analysis was performed using two-sided log-rank tests. Data are mean ± s.e.m. Details of the statistical analyses and replicate data of lifespan experiments are provided in Supplementary Table 7. Gel source data are provided in Supplementary Fig. 1. Source data

Given the limited uptake of exogenous biopterins in worms, we used a genetic approach to assess the full potential of these metabolites to extend longevity. Ubiquitous overexpression of *cat-4* in somatic tissues resulted in a substantial increase in cold-induced longevity (Extended Data Fig. 7f). Notably, overexpression of *cat-4* in the muscle alone was sufficient to increase organismal levels of bioactive BH4 and extend lifespan not only at 15 °C, but also at 20 °C and 25 °C (Fig. 3a,b and Extended Data Fig. 7g–i). Moreover, muscle-specific *cat-4* overexpression also extended lifespan in loss-of-function *trpa-1* mutant worms across different temperatures (Extended Data Fig. 7j–l). These findings indicate that increasing CAT-4 activity in muscle promotes longevity independently of both temperature and the cold-sensitive channel TRPA-1.

Consistent with a systemic protective effect, muscle-specific *cat-4* overexpression prevented age-related accumulation and aggregation of intestinal IFB-2 protein at standard temperature (Fig. 3c and Extended Data Fig. 7m). As IFB-2 aggregation contributes to loss of intestinal integrity and subsequent bacterial colonization during ageing^22^, we examined whether CAT-4 prevents this deleterious process. Indeed, muscle-specific *cat-4* overexpression reduced bacterial invasion of the intestine in aged worms at standard temperature (Fig. 3d,e). We then tested whether muscle-derived BH2 also acts in neurons. Neuronal-specific downregulation of either BH2 uptake (using *ent-1* RNAi) or its conversion to BH4 through the salvage pathway (through *dhfr-1* RNAi) significantly reduced the lifespan extension conferred by muscle-specific *cat-4* overexpression at standard temperature, but had no effect in control worms (Extended Data Fig. 7n,o). These results further demonstrate that elevated BH2 production in muscle extends lifespan in a cell non-autonomous manner and requires both BH2 uptake and its conversion into bioactive BH4 in distal tissues, including the intestine and neurons.

We next examined whether increasing CAT-4 levels directly in the tissues that use BH2 is sufficient to promote longevity. While muscle-specific *cat-4* overexpression had the strongest longevity effect, intestinal overexpression also markedly extended lifespan at both 15 °C and 20 °C (Fig. 3f,g). Although to a lesser extent, *cat-4* overexpression in neurons also increased lifespan under both cold and standard temperatures (Fig. 3f,g). Together, these data suggest that, while elevated BH2 synthesis in muscle has a central role in longevity, increasing *cat-4* expression in other tissues partially mimics its beneficial effects.

## Tyrosine synthesis extends lifespan

**Extended Data Fig. 8:**
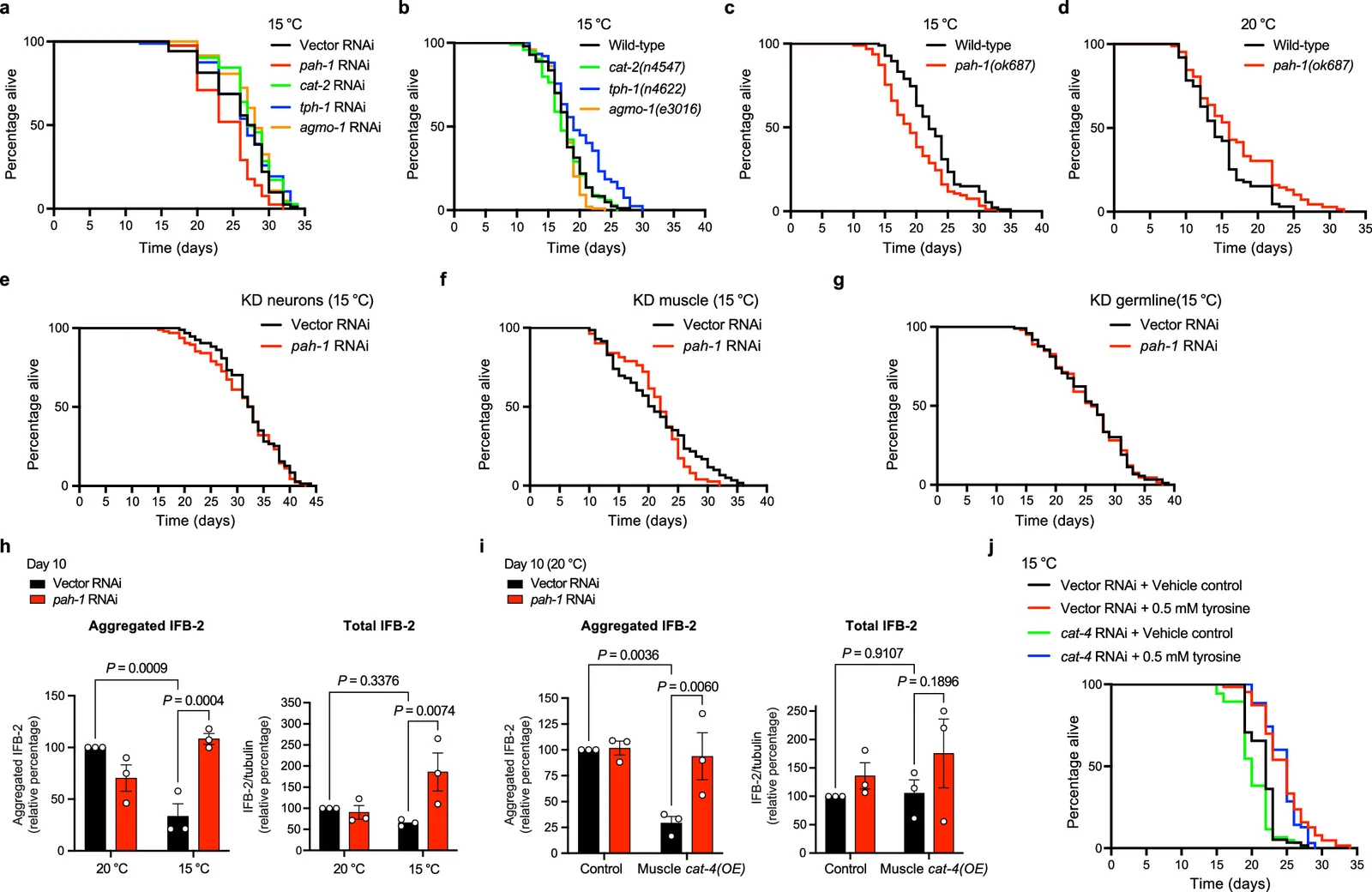
*pah-1* mutant worms are short-lived at cold but not at standard temperature. **a**, Knockdown of *pah-1* after development shortens cold-induced longevity in wild-type worms (*P* = 0.0002). In contrast, knockdown of *cat-2* (*P* = 0.1304), *tph-1* (*P* = 0.2975), or *agmo-1* (*P* = 0.0918) does not shorten longevity. **b**, Lifespan of *cat-2(n4547)*, *tph-1(n4622)* and *agmo-1(e3016)* mutants at 15 °C. *cat-2* (*P* = 0.5675) and *agmo-1* (*P* = 0.0657) mutants do not have reduced lifespan, whereas *tph-1* mutants have increased lifespan (*P* = 0.0007). **c**, *pah-1(ok687)* mutant worms are short-lived at 15 °C (*P* = 0.0003). **d**, *pah-1(ok687)* mutant worms live longer at 20 °C (*P* = 0.0051). **e**, Knockdown (KD) of *pah-1* in neurons does not shorten cold-induced longevity (*P* = 0.4936). **f**, Knockdown of *pah-1* in the muscle alone does not shorten cold-induced longevity (*P* = 0.2377). **g**, Germline-specific knockdown of *pah-1* does not reduce cold-induced longevity (*P* = 0.9385). **h**,**i**, Quantification of aggregated and total IFB-2 from Fig. 4b (**h**) and Fig. 4c (**i**), relative to the corresponding control groups (mean ± s.e.m.; *n* = 3 independent experiments). Statistical comparisons were made by two-way ANOVA with Fisher’s LSD test. **j**, Tyrosine supplementation rescues the reduction in cold-induced longevity caused by *cat-4* knockdown (Vector RNAi + vehicle control vs Vector RNAi + 0.5 mM tyrosine: *P* < 0.0001; Vector RNAi + vehicle control vs *cat-4* RNAi + vehicle control: *P* = 0.0032; Vector RNAi + vehicle control vs *cat-4* RNAi + 0.5 mM tyrosine: *P* < 0.0001). For lifespan experiments: *n* = 96 worms per condition; two-sided log-rank test; representative of at least two independent experiments. Mean ± s.e.m. and replicate data of lifespan experiments are provided in Supplementary Table 7. Source data

To determine how utilization of BH4 as an enzymatic cofactor contributes to cold-induced longevity, we performed a targeted RNAi screen against the four BH4-dependent enzymes expressed in *C. elegans*: phenylalanine hydroxylase (PAH-1), tryptophan hydroxylase (TPH-1), tyrosine hydroxylase (CAT-2) and alkylglycerol monooxygenase (AGMO-1)^18^. Among them, knockdown of *pah-1* after development shortened lifespan, whereas knockdown of the other BH4-dependent enzymes did not affect cold-induced longevity (Extended Data Fig. 8a). Consistently, loss-of-function *tph-1*, *cat-2* and *agmo-1* mutants were not short-lived, whereas *pah-1* mutants exhibited reduced longevity specifically at cold temperature (Extended Data Fig. 8b–d).

**Fig. 4:**
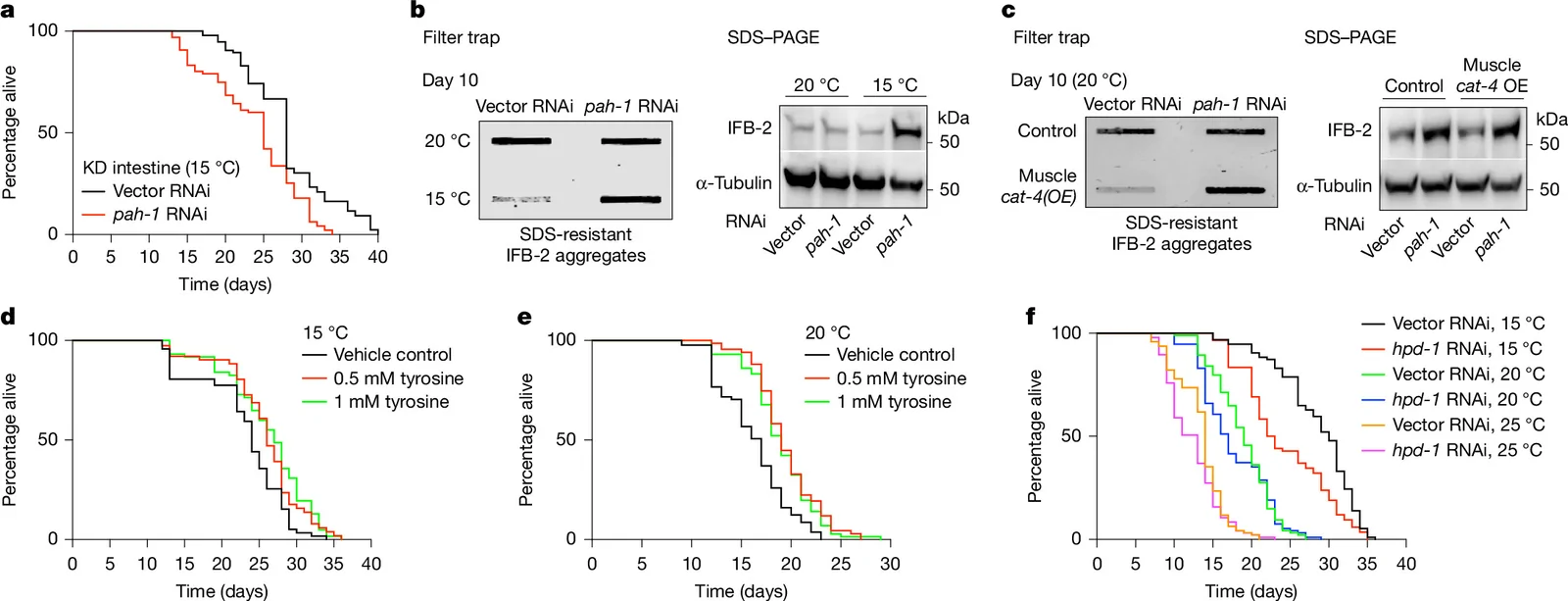
Elevated tyrosine synthesis through PAH-1 extends lifespan. **a**, Intestinal *pah-1* RNAi after development shortens cold-induced longevity (*P* < 0.0001). **b**, Filter-trap analysis of intestinal IFB-2 aggregation in day 10 adult wild-type worms at 20 °C and 15 °C after vector control or *pah-1* RNAi. Right, SDS–PAGE and immunoblotting of IFB-2 and the α-tubulin loading control. Images are representative of three independent experiments; quantification is shown in Extended Data Fig. 8h. **c**, Filter-trap analysis of IFB-2 aggregation in day 10 adult control and muscle-specific *cat-4*-overexpressing worms at 20 °C. Right, SDS–PAGE and immunoblotting of IFB-2 and α-tubulin. Images are representative of three independent experiments; quantification is shown in Extended Data Fig. 8i. **d**,**e**, Tyrosine supplementation after development extends lifespan at 15 °C (**d**; 0.5 mM, *P* = 0.0103; 1 mM, *P* = 0.0006) and 20 °C (**e**; 0.5 mM, *P* < 0.0001; 1 mM, *P* < 0.0001). The vehicle control was HCl. **f**, Knockdown of *hpd-1* after development shortens lifespan at 15 °C (*P* < 0.0001) but not at 20 °C (*P* = 0.4504) or 25 °C (*P* = 0.0826). In lifespan assays, *n* = 96 worms per condition; data are representative of at least two independent experiments. Statistical comparisons were performed using two-sided log-rank tests. Data are mean ± s.e.m. Replicate data and statistical analyses of lifespan experiments are provided in Supplementary Table 7. Gel source data are provided in Supplementary Fig. 1.

Among the tissues involved in cold-induced longevity, we found that PAH-1 activity in the intestine is particularly important for lifespan extension (Fig. 4a and Extended Data Fig. 8e–g). Consistent with this, knockdown of *pah-1* increased both the levels and aggregation of the intestinal intermediate filament IFB-2, thereby abolishing the protective effects of cold temperature on age-associated IFB-2 aggregation (Fig. 4b and Extended Data Fig. 8h). While muscle-specific overexpression of *cat-4* was sufficient to prevent IFB-2 aggregation during ageing at standard temperature, this protective effect was lost after *pah-1* knockdown (Fig. 4c and Extended Data Fig. 8i).

PAH-1 catalyses the synthesis of tyrosine, a metabolite that we found to be upregulated at cold temperature (Fig. 1a). Exogenous supplementation with tyrosine extended lifespan not only at cold temperature, but also at standard temperature (Fig. 4d,e). Importantly, tyrosine supplementation rescued the reduction in cold-induced longevity caused by *cat-4* knockdown (Extended Data Fig. 8j), further supporting a functional role of regulated tyrosine metabolism in mediating the longevity effects of elevated BH2 synthesis.

**Extended Data Fig. 9:**
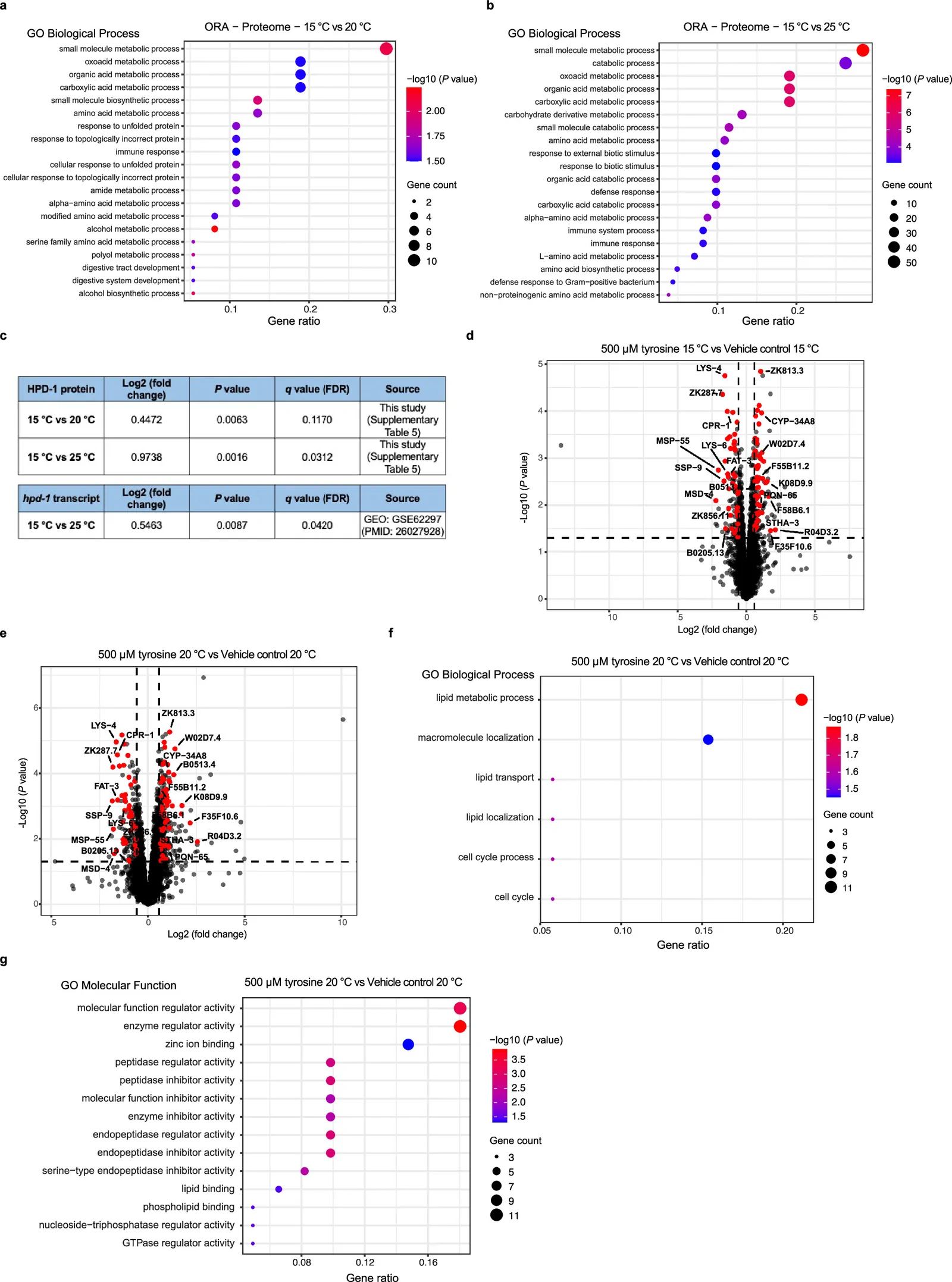
HPD-1, a key enzyme in tyrosine catabolism, is upregulated at cold temperature. **a**,**b**, Over-representation analysis (ORA) of differentially abundant proteins in day-5 adult wild-type *C. elegans* comparing 15 °C with 20 °C (**a**) or 25 °C (**b**). The most significantly enriched GO Biological Process terms are shown. *P* values were calculated using a one-sided (right-sided) hypergeometric test. **c**, Cold temperature increases *hpd-1* expression at both the protein level (Supplementary Table 5) and transcript level (GEO: GSE62297; PMID: 26027928). Statistical analysis of protein levels was performed using a two-sided *t*-test, and statistical analysis of transcript levels was performed using a two-sided empirical Bayes-moderated *t*-test. *P* values were adjusted for multiple testing using the false discovery rate (FDR) method. **d**,**e**, Quantitative proteomic analysis of day-3 adult worms treated with 500 µM tyrosine or vehicle control (HCl) at 15 °C (**d**) or 20 °C (**e**). Treatment was initiated after development. Each point represents a quantified protein. The x axis indicates log2 fold change in protein abundance, and the y axis shows -log10 (*P* value) from limma-based differential protein abundance analysis using two-sided empirical Bayes-moderated *t*-tests. Dashed vertical lines indicate the fold-change threshold (log2 fold change > 0.58 or < −0.58; corresponding to a 1.5-fold change), and the horizontal dashed line indicates the significance threshold (*P* < 0.05). Proteins significantly altered at both temperatures are highlighted in red. The ten most strongly upregulated and downregulated shared proteins are labelled. See Supplementary Table 6 for the complete dataset. **f**,**g**, ORA of GO Biological Process (**f**) and GO Molecular Function (**g**) terms among proteins differentially abundant in day-3 adult worms treated with 500 µM tyrosine at 20 °C. The most significantly enriched GO terms are shown. *P* values were calculated using a one-sided (right-sided) hypergeometric test.

To gain insights into the downstream mechanisms by which tyrosine promotes longevity, we performed over-representation analysis (ORA) of the differentially abundant proteins in worms at 15 °C relative to either 20 °C or 25 °C (Supplementary Table 5). Notably, we found strong enrichment for proteins involved in small-molecule and amino acid metabolic processes (Extended Data Fig. 9a,b). Within these pathways, we identified increased levels of 4-hydroxyphenylpyruvate dioxygenase (HPD (human), HPD-1 (*C. elegans*); Extended Data Fig. 9c)—a key enzyme in the canonical tyrosine catabolic pathway that ultimately generates acetoacetate and fumarate, thereby linking tyrosine metabolism to central carbon metabolism^25^. Moreover, analysis of published transcriptomic datasets also revealed increased *hpd-1* mRNA levels at cold temperature^12^ (Extended Data Fig. 9c). We found that loss of *hpd-1* significantly reduced cold-induced longevity (Fig. 4f), supporting a functional contribution of tyrosine catabolism to lifespan extension at low temperature. Although *hpd-1* knockdown also caused a slight decrease in lifespan at 20 °C and 25 °C, these effects did not reach statistical significance (Fig. 4f).

To identify molecular pathways that are responsive to elevated tyrosine levels, we performed quantitative proteomic analyses after tyrosine supplementation at both 15 °C and 20 °C (Supplementary Table 6). These analyses revealed 54 commonly upregulated proteins and 42 commonly downregulated proteins after tyrosine supplementation across both temperatures (Extended Data Fig. 9d,e). ORA of the differentially abundant proteins identified significant enrichment for lipid metabolic processes and enzyme regulator activity (Extended Data Fig. 9f,g). Together, these data suggest that tyrosine promotes longevity through specific downstream regulatory and metabolic pathways, including HPD-1-dependent tyrosine catabolism.

## Muscle BH2 enhances systemic proteostasis

**Extended Data Fig. 10:**
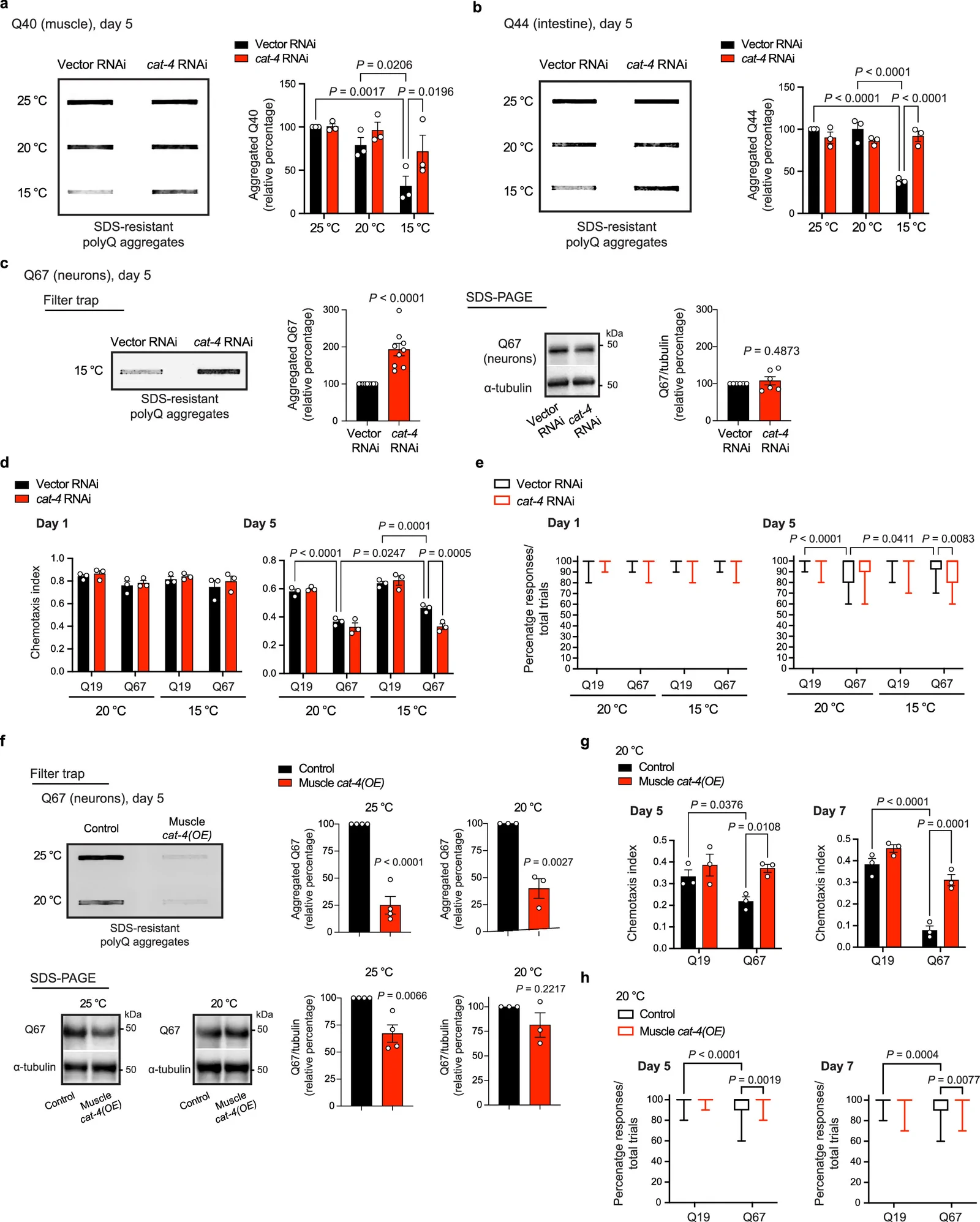
Muscle CAT-4 overexpression reduces pathological protein aggregation in distal tissues at standard and warmer temperatures. **a**,**b**, Filter-trap analysis of aggregated polyQ40 in muscle (**a**) and polyQ44 in intestine (**b**) following *cat-4* RNAi in day-5 adult *C. elegans* at the indicated temperatures (mean ± s.e.m. relative to 25 °C + Vector RNAi, *n* = 3 independent experiments). **c**, Filter-trap analysis of day-5 adult worms expressing polyQ67 in neurons at 15 °C. Right: SDS–PAGE with antibodies to GFP and α-tubulin. Graphs: aggregated polyQ67 and total polyQ67 levels (corrected for α-tubulin loading control). Mean ± s.e.m. relative to vector RNAi; filter trap: *n* = 9 independent experiments; SDS-PAGE: *n* = 6 independent experiments. **d**, Chemotaxis index towards 0.5% benzaldehyde of control Q19 and Q67 worms at days 1 and 5 of adulthood (mean ± s.e.m., *n* = 3 independent experiments). **e**, Percentage of nose touch responses/total trials per worm at days 1 and 5 of adulthood (*n* = 40 worms per condition). The box plots represent the 25th–75th percentiles, the lines depict the median and the whiskers show the minimum–maximum values. In **a–e**, RNAi was initiated after development. **f**, Filter-trap analysis of neuronal Q67 worms overexpressing *cat-4* in muscle at 25 °C and 20 °C. Below: SDS–PAGE with antibodies to GFP and α-tubulin. Graphs: aggregated polyQ67 and total polyQ67 levels (corrected for α-tubulin). Mean ± s.e.m. relative to control Q67 worms at the same temperature; 25 °C: *n* = 4, 20 °C: *n* = 3 independent experiments. **g**, Chemotaxis index of Q19 and Q67 worms with muscle-specific *cat-4* overexpression at days 5 and 7 (mean ± s.e.m., *n* = 3 independent experiments). **h**, Percentage of nose-touch responses at days 5 and 7 (*n* = 40 worms per condition). Box plots show 25th–75th percentiles, median and minimum–maximum values. Statistics: two-way ANOVA with Tukey’s multiple-comparison test (**a**,**b**,**d**,**e**), two-sided t-test for unpaired samples (**c**,**f**), and two-way ANOVA with Fisher’s LSD test (**g**,**h**). For gel source data, see Supplementary Fig. 1. Source data

Loss of proteostasis during ageing is a major risk factor for diseases characterized by pathological protein aggregation^26^. Moderately cold temperatures suppress disease-related protein aggregation across tissues, including polyQ-expanded proteins in *C. elegans* models of Huntington’s disease^2,9^. Knockdown of *cat-4* abolished this protective effect and increased aggregation without altering total polyQ levels (Extended Data Fig. 10a–c), indicating that CAT-4 activity modulates aggregation rather than overall protein amounts.

Consistent with the cell non-autonomous effects of muscle CAT-4 activity, loss of *cat-4* increased aggregation at cold temperature not only in muscle, but also in worms expressing polyQ-expanded proteins exclusively in the intestine or neurons (Extended Data Fig. 10a–c). In neurons, polyQ67 aggregation causes behavioural deficits during ageing, including impaired chemotaxis and nose-touch avoidance compared with wild-type or polyQ19 controls^27,28^. Cold temperature partially rescued these neuronal deficits, whereas loss of *cat-4* abolished this protection (Extended Data Fig. 10d,e). Conversely, muscle-specific overexpression of *cat-4* reduced neuronal polyQ67 aggregation under both standard (20 °C) and warmer (25 °C) temperatures, preventing deficits in chemotaxis and nose-touch avoidance (Extended Data Fig. 10f–h). These results support a cell non-autonomous role for muscle CAT-4 activity in maintaining proteostasis in distal tissues.

**Extended Data Fig. 11:**
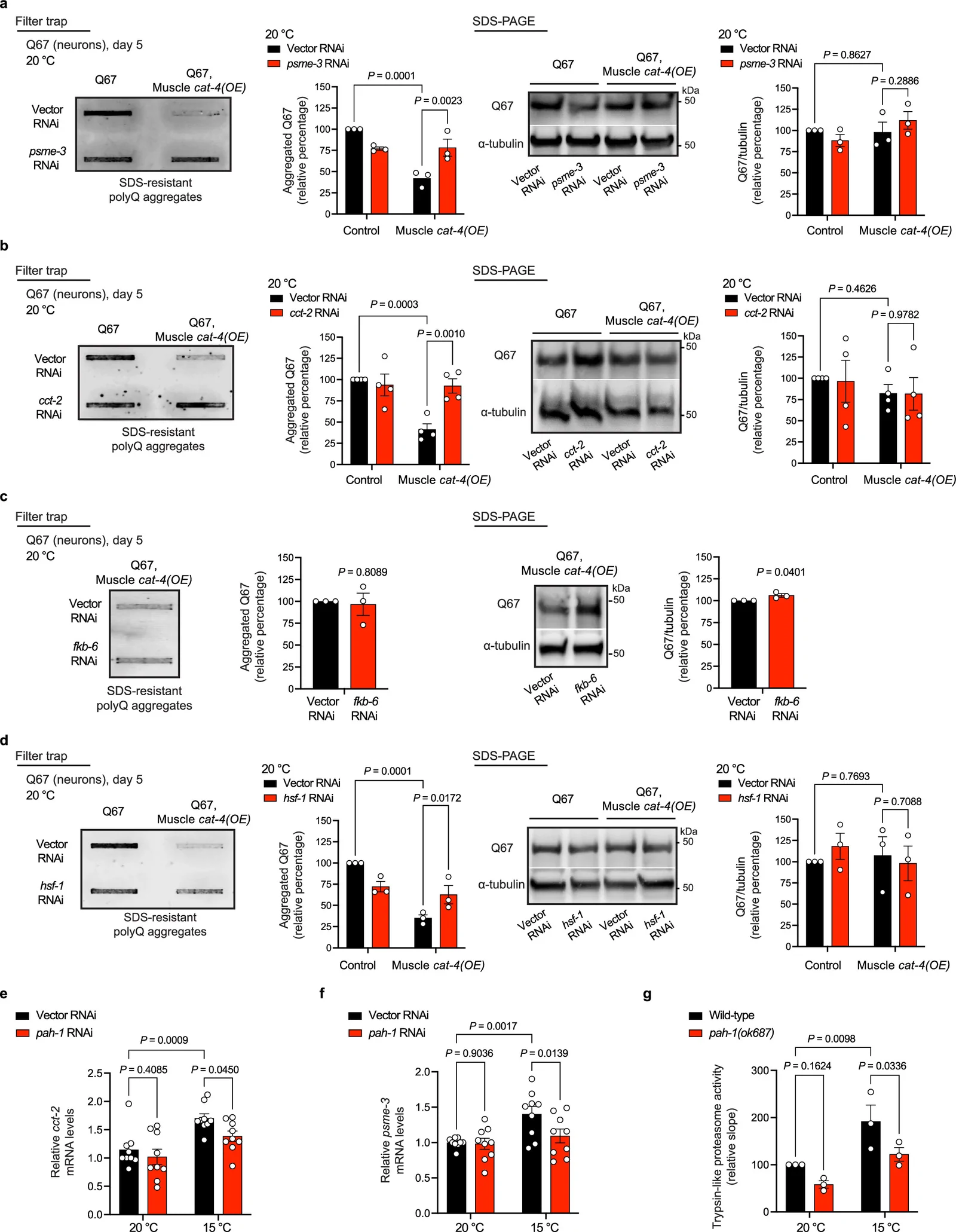
Proteasome and TRiC/CCT activity contribute to CAT-4-mediated proteostasis. **a–d**, Filter-trap analysis of aggregated polyQ67 in neurons (detected by anti-GFP antibody) in control and muscle-specific *cat-4* overexpressing worms following RNAi treatment against *psme-3* (**a**), *cct-2* (**b**), *fkb-6* (**c**), or *hsf-1* (**d**) at 20 °C. The corresponding SDS–PAGE immunoblots show total polyQ67 levels (anti-GFP) and α-tubulin loading controls. Graphs represent aggregated polyQ67 and total polyQ67 (corrected for α-tubulin) levels relative to Q67 control worms + Vector RNAi (mean ± s.e.m.; *n* = 3 independent experiments for **a**, **c**, and **d**; *n* = 4 independent experiments for **b**). **e**,**f**, *cct-2* (e) and *psme-3* (f) mRNA levels following *pah-1* knockdown at 15 °C and 20 °C (mean ± s.e.m. relative to 20 °C + Vector RNAi, *n* = 9 biological replicates from two independent experiments). **g**, Trypsin-like proteasome activity in wild-type and *pah-1(ok687)* worms at 15 °C and 20 °C (mean ± s.e.m. relative to wild-type 20 °C, *n* = 3 independent experiments). In all the experiments, RNAi treatment was initiated after development and worms were analysed at day 5 of adulthood. Statistical comparisons were made by two-way ANOVA with Fisher’s LSD test (**a**,**b**,**d**–**g**) and two-sided t-test for unpaired samples (**c**). For gel source data, see Supplementary Fig. 1. Source data

We next investigated how elevated CAT-4 activity enhances proteostasis capacity. Cold temperature induces the proteasome activator PA28γ (human, encoded by *PSME3*)/PSME-3 (*C. elegans*), increasing trypsin-like proteasome activity^2^. Knockdown of *psme-3* attenuated the ability of muscle-specific *cat-4* overexpression to suppress neuronal polyQ67 aggregation, without altering total polyQ levels (Extended Data Fig. 11a), suggesting a broader defect in proteostasis rather than impaired degradation of polyQ proteins per se. Thus, CAT-4 activity appears to influence proteostasis through additional mechanisms. Besides proteasome activity, cold temperature also induces expression of different subunits of the TRiC/CCT complex^10^, a chaperonin that prevents polyQ-expanded aggregation^29–31^. Knockdown of *cct-2* subunit completely abolished the protective effects of muscle-specific *cat-4* overexpression at standard temperature (Extended Data Fig. 11b). By contrast, knockdown of *fkb-6*, a cold-induced co-chaperone of HSP90^10^, had no effect on polyQ67 aggregation (Extended Data Fig. 11c). We also tested *hsf-1*, a key transcription factor that protects against protein misfolding and aggregation^32^. Loss of *hsf-1* partially attenuated the protective effects of muscle-specific *cat-4* overexpression (Extended Data Fig. 11d), although less strongly than *cct-2* or *psme-3* knockdown.

Finally, we assessed whether PAH-1 activity contributes to these proteostasis mechanisms. Indeed, loss of *pah-1* reduced the cold-induced upregulation of *cct-2* and *psme-3* expression, as well as the increase in trypsin-like proteasome activity (Extended Data Fig. 11e–g). Together, these results indicate that elevated CAT-4 activity and subsequent BH4-dependent tyrosine synthesis enhance proteostasis through different downstream mechanisms, including proteasome activity and the TRiC/CCT chaperonin complex.

## BH2 enhances proteostasis in human cells

**Fig. 5:**
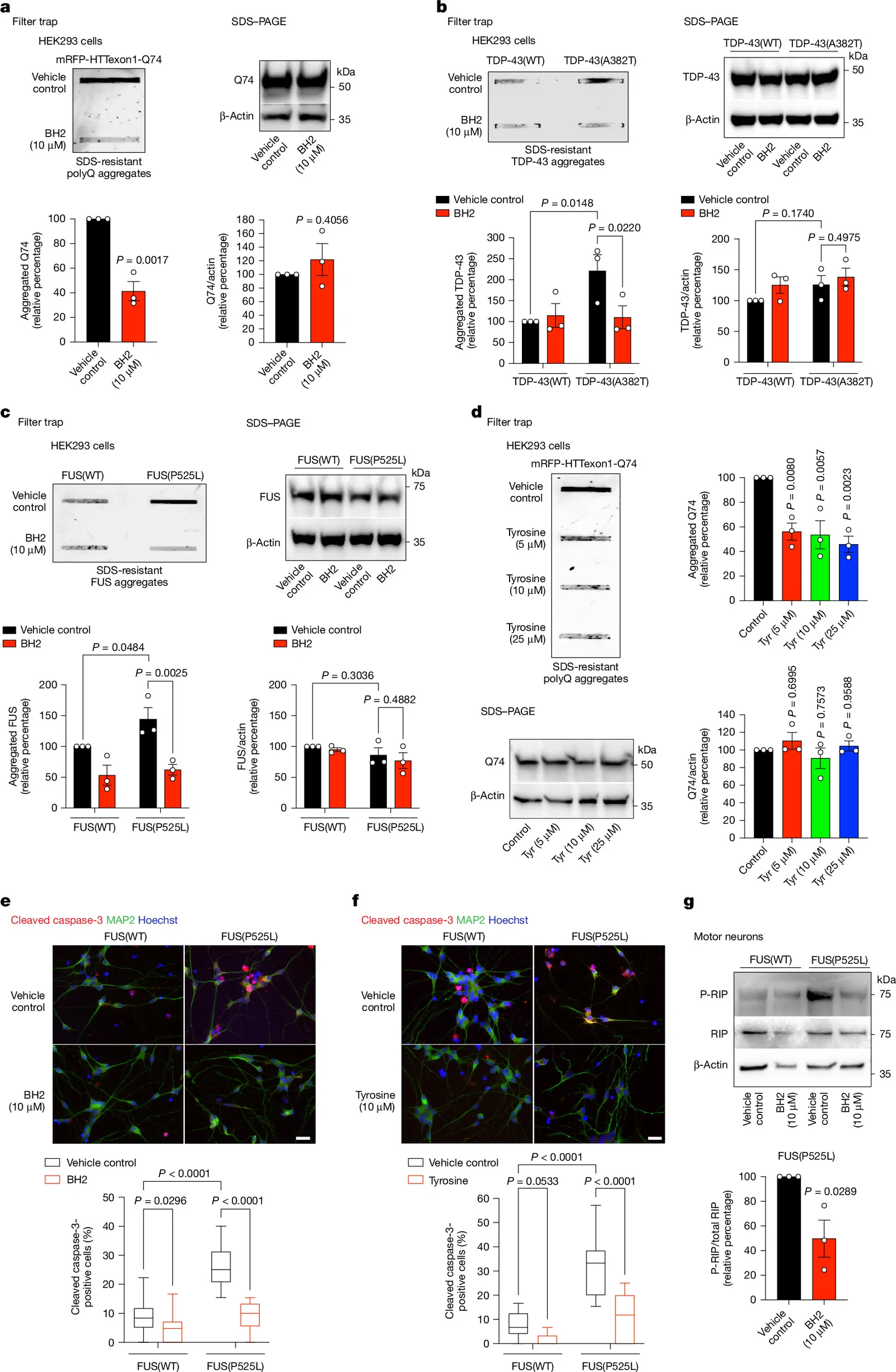
BH2 supplementation prevents aggregation of distinct disease-related proteins in human cells. **a**, Filter-trap and SDS–PAGE analysis of mRFP-HTTexon1-Q74 in HEK293 cells at standard (37 °C) temperature. Cells were treated with 10 µM BH2 or vehicle control (DMSO) for 3 days. The graphs show aggregated Q74 and total Q74 levels (normalized to the β-actin loading control) relative to the vehicle control. Data are mean ± s.e.m. *n* = 3 independent experiments. **b**,**c**, Filter-trap and SDS–PAGE analysis of aggregated and total TDP-43(A382T) (**b**) or FUS(P525L) (**c**) in HEK293 cells treated with 10 µM BH2 or vehicle control for 3 days. Quantifications are relative to the corresponding wild-type (WT) + vehicle control. Data are mean ± s.e.m. *n* = 3 independent experiments. **d**, Filter-trap and SDS–PAGE analysis of aggregated and total mRFP-HTTexon1-Q74 in HEK293 cells treated with the indicated concentrations of tyrosine or vehicle control (HCl) for 3 days. Data are mean ± s.e.m. relative to the vehicle control. *n* = 3 independent experiments. **e**,**f**, Immunocytochemistry analysis of mutant *FUS*^*P525L/P525L*^ and isogenic control *FUS*^*WT/WT*^ iPS-cell-derived motor neurons treated with 10 µM BH2 (**e**) or tyrosine (**f**) for 3 days. Cleaved caspase-3 (red), MAP2 (green) and Hoechst (blue) staining mark apoptosis, neurons and nuclei, respectively. Scale bars, 20 µm (**e**,**f**). The box plots show the 25th–75th percentiles (box limits), median (centre line) and minimum–maximum values (whiskers) of the percentage of cleaved-caspase-3-positive cells among MAP2-positive cells; *n* = 15 biological replicates from three independent experiments. **g**, Western blot analysis of phosphorylated RIP (P-RIP) at Ser166, total RIP and β-actin in wild-type and *FUS*^*P525L/P525L*^ iPS-cell-derived motor neurons treated with 10 µM BH2. Data are mean ± s.e.m. relative to *FUS*^*P525L/P525L*^ + vehicle control. *n* = 3 independent experiments. In **a**–**g**, images are representative of three independent experiments. Statistical comparisons were performed using two-sided unpaired *t*-tests (**a**,**g**), two-way ANOVA with Fisher’s LSD test (**b**,**c**,**e**,**f**) and one-way ANOVA with Dunnett’s multiple-comparison test (**d**). Gel source data are provided in Supplementary Fig. 1. Source data

We next examined whether BH2 supplementation prevents disease-related protein aggregation in human cells at standard temperature (37 °C). To this end, we generated HEK293 cells expressing mutant huntingtin exon 1 containing polyQ74 repeats, which is prone to aggregation^33,34^ (Fig. 5a). We found that supplementation with 10 µM BH2 markedly reduces polyQ aggregation without affecting overall protein levels (Fig. 5a). To determine whether BH2 also protects against aggregation of other disease-associated proteins, we assessed mutant variants of TDP-43 and FUS associated with amyotrophic lateral sclerosis (ALS). In contrast to their wild-type counterparts, the ALS-causing mutant TDP-43(A382T) and FUS(P525L) variants aggregated in HEK293 human cells (Fig. 5b,c). However, BH2 supplementation prevented aggregation of both mutant proteins (Fig. 5b,c). Consistent with this mechanism, exogenous tyrosine supplementation also reduced pathological protein aggregation (Fig. 5d).

Prompted by these findings, we examined whether BH2 attenuates disease-related neurodegeneration in human cells. Motor neurons differentiated from induced pluripotent stem (iPS) cells expressing the severe ALS-linked FUS(P525L) mutation exhibit elevated apoptosis compared with isogenic control cells^35^. Notably, supplementation with either BH2 or tyrosine reduced apoptosis in ALS neurons (Fig. 5e,f). ALS motor neurons also showed elevated phosphorylation of receptor-interacting protein (RIP) kinase, a marker of necroptotic cell death, which was reduced by BH2 supplementation (Fig. 5g), further supporting a protective effect against disease-related neurodegeneration.

**Extended Data Fig. 12:**
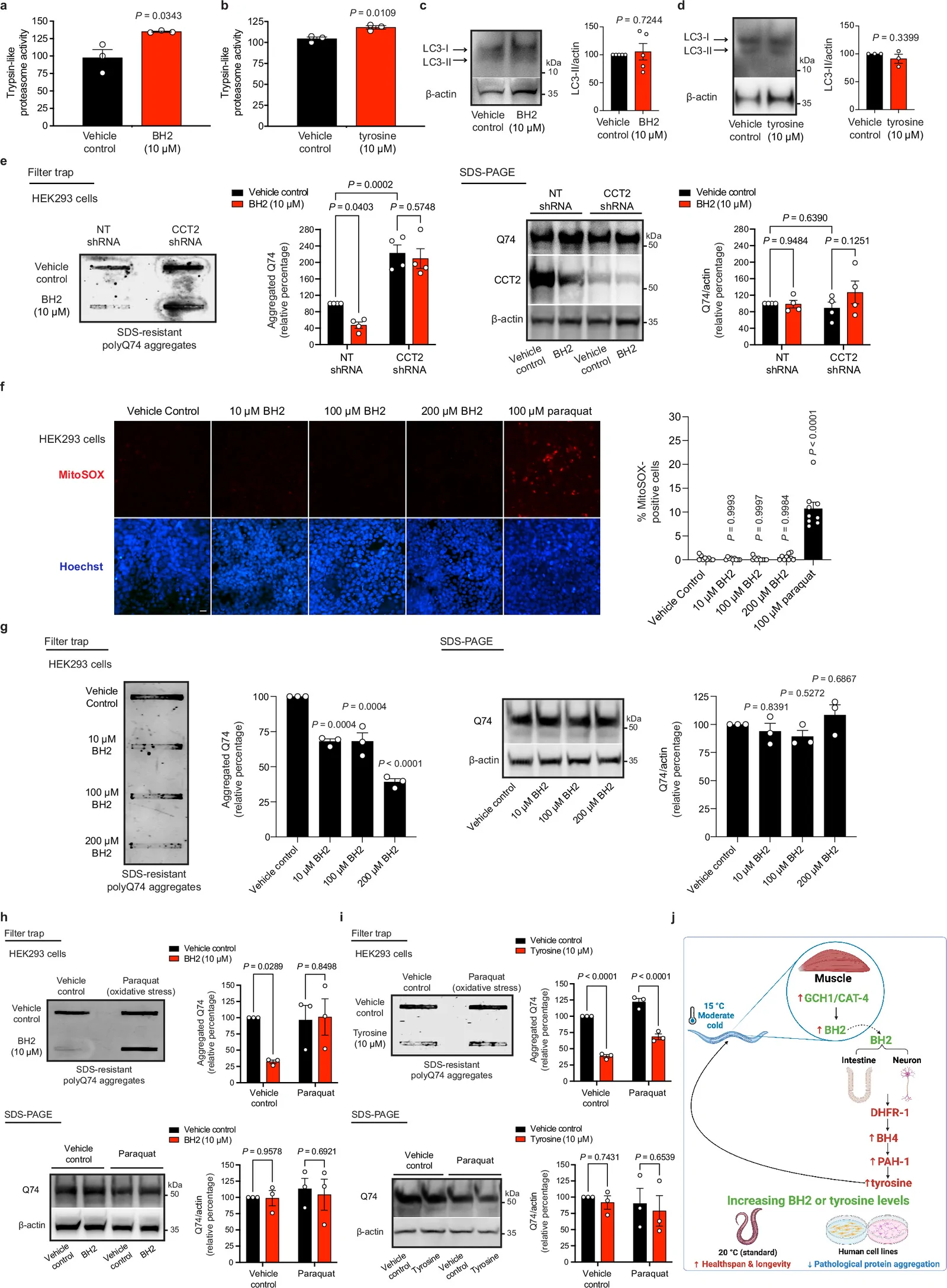
Tyrosine bypasses oxidative stress conditions that limit BH2-to-BH4 conversion. **a**,**b**, Trypsin-like proteasome activity in HEK293 cells treated with 10 µM BH2 (**a**, Vehicle control: DMSO) or tyrosine (**b**, Vehicle control: HCl) for 3 days at 37 °C (mean ± s.e.m. relative to vehicle control, *n* = 3 independent experiments). **c**,**d**, Western blot analysis using antibodies against LC3 and β-actin in HEK293 cells treated with 10 µM BH2 (**c**) or tyrosine (**d**). Graphs: relative levels of LC3-II (corrected for β-actin) to vehicle control (mean ± s.e.m.; BH2: *n* = 5, tyrosine: *n* = 3 independent experiments). **e**, Filter-trap analysis using anti-mCherry antibody to detect aggregated mRFP-HTTexon1-Q74 (Q74) in HEK293 cells at 37 °C. Non-targeting (NT) shRNA or CCT2 shRNA-expressing cells were treated with 10 µM BH2 or vehicle control (DMSO) for 3 days. Right: SDS–PAGE with antibodies to mCherry, CCT2 and β-actin. Graphs: aggregated Q74 and total Q74 levels (corrected for β-actin). Mean ± s.e.m. relative to NT shRNA + vehicle control, *n* = 4 independent experiments. **f**, Mitochondrial superoxide detected with MitoSOX following treatment with BH2 or paraquat as a positive control. Graph: Percentage of MitoSOX-positive cells (mean ± s.e.m.; *n* = 9 biological replicates from two independent experiments; Vehicle: 1,894 total nuclei analysed, 10 µM BH2: 2,864, 100 µM BH2: 2,354, 200 µM BH2: 2,277, 100 µM paraquat: 1,561). **g**, Aggregated and total Q74 following treatment with the indicated BH2 concentrations (mean ± s.e.m. relative to vehicle control, *n* = 3 independent experiments). **h**,**i**, Aggregated and total Q74 following BH2 (**h**) or tyrosine (**i**) treatment under paraquat-induced oxidative stress (mean ± s.e.m., *n* = 3 independent experiments). Statistics: two-sided t-test for unpaired samples (**a–d**), one-way ANOVA with Dunnett’s multiple comparison test (**f**,**g**), and two-way ANOVA with Fisher’s LSD test (**e**,**h**,**i**). For gel source data, see Supplementary Fig. 1. **j**, Schematic of the proposed mechanism underlying cold-induced longevity and proteostasis. Created in BioRender. Vilchez, D. (2026) https://BioRender.com/crckozz. Source data

We then examined whether BH2 and tyrosine enhance proteostasis capacity through mechanisms similar to those activated by cold temperature in *C. elegans*. Indeed, both treatments caused a slight but significant increase in trypsin-like proteasome activity in HEK293 cells at standard temperature (Extended Data Fig. 12a,b). By contrast, neither treatment increased LC3 lipidation, a marker of autophagosomes (Extended Data Fig. 12c,d). As BH2 suppressed aggregation without significantly altering total levels of the pathological proteins (Fig. 5a–c), we examined additional proteostasis mechanisms. Consistent with our findings in worms, knockdown of the TRiC/CCT chaperonin subunit *CCT2* reduced the ability of BH2 to suppress pathological protein aggregation, identifying TRiC/CCT-complex-dependent folding capacity as a key contributor to BH2-mediated proteostasis enhancement in human cells (Extended Data Fig. 12e).

In contrast to in *C. elegans*, mammalian cells express nitric oxide synthases (NOSs), which require BH4 as a cofactor^16,18^. Under conditions that decrease intracellular BH4 availability, such as oxidative stress—which promotes excessive oxidation of BH4 to BH2—or impaired DHFR activity, BH2 can compete with BH4 for binding to endothelial NOS (eNOS) in the vascular system^36–38^. This shifts eNOS activity from nitric oxide production toward superoxide generation, a process known as eNOS uncoupling^38^. Importantly, eNOS uncoupling is associated primarily with reduced BH4 availability and a consequent decrease in the BH4/BH2 ratio, rather than with elevated BH2 levels per se or BH2 supplementation. In fact, when cells retain an intact DHFR-dependent salvage pathway, BH2 supplementation increases intracellular BH4 levels and alleviates eNOS uncoupling under oxidative stress^36,37^. In contrast to paraquat-induced oxidative stress, neither 10 µM BH2 nor a 20-fold higher concentration increased the percentage of cells with detectable superoxide levels (Extended Data Fig. 12f). Moreover, even high doses of BH2 reduced pathological protein aggregation (Extended Data Fig. 12g), supporting the proteostasis benefits of BH2 supplementation without detectable superoxide production indicative of BH4 imbalance.

Given that oxidative stress promotes BH4 oxidation to BH2, we hypothesized that such conditions might limit the therapeutic potential of BH2 supplementation. Indeed, paraquat-induced oxidative stress abolished the protective effect of BH2 on protein aggregation (Extended Data Fig. 12h). By contrast, tyrosine supplementation retained its protective effect under oxidative stress (Extended Data Fig. 12i), suggesting that this downstream effector of the pathway can bypass conditions in which BH2 cannot be efficiently converted into BH4.

## Discussion

Here we identify BH2 as a cold-induced metabolite that links low temperature to systemic regulation of longevity and proteostasis (Extended Data Fig. 12j). Moderate cold increases CAT-4-dependent BH2 synthesis in muscle, from which BH2 is transported to distal tissues and converted into bioactive BH4 by DHFR-1. This establishes muscle as a metabolic hub that produces a pro-longevity signal of which the activity can be regulated locally in recipient tissues. BH4-dependent PAH-1 activity promotes tyrosine synthesis, and tyrosine supplementation recapitulates the longevity benefits of this pathway even at normal temperature, identifying the PAH-1–tyrosine axis as an important downstream mediator of the cold-induced BH2 pathway. The pathway also enhances organismal proteostasis: increasing BH2 synthesis in muscle suppresses pathological protein aggregation and preserves neuronal and intestinal function in distal tissues. Together, these findings expand the concept of cold-induced longevity beyond a predominantly cell-autonomous response to a systemic metabolic communication mechanism that coordinates longevity and proteostasis across tissues.

The ability of BH2 and tyrosine to suppress disease-associated protein aggregation and neurodegeneration-related phenotypes in human cells further supports functional conservation of this pathway. However, its translational potential may depend on the cellular context in which BH2 is converted to BH4. In mammals, BH4 is also required by NOS, and oxidative stress or impaired DHFR activity can disrupt the BH4/BH2 balance and promote eNOS uncoupling in endothelial cells^36–38^. BH2 supplementation did not increase detectable superoxide production in our cellular models, even at high concentrations, although these experiments do not directly address potential effects on NOS uncoupling in vivo. Consistent with the importance of BH2-to-BH4 conversion, we found that oxidative stress limits the protective effect of BH2, whereas tyrosine retains its protective effect. Thus, tyrosine may provide an alternative means of engaging this pathway when BH2-to-BH4 conversion is impaired. More broadly, our metabolomic analyses identified numerous additional metabolites induced by cold, raising the possibility that BH2 is one component of a larger network of systemic metabolic signals that coordinates the beneficial effects of low temperature. Defining these pathways may reveal additional strategies to reproduce the health-promoting effects of cold without altering body temperature.

## Methods

### *C. elegans* strains and maintenance

*C. elegans* were maintained at 20 °C on standard nematode growth medium (NGM) seeded with OP50 *E. coli*^39^. In all the experiments, worms were raised at the standard temperature (20 °C) until adulthood and then transferred to the temperatures indicated in the corresponding figures (15 °C, 20 °C and 25 °C). All of the experiments were performed using hermaphrodite adult worms at the ages indicated in the corresponding figures and figure legends.

Wild-type (N2), TQ233 (*trpa-1(ok999)* IV, outcrossed 6× to wild-type N2 worms), LC141 (*him-8(e1489)* IV; *cat-4(e3015)* V, outcrossed 6× to N2), LC35 (*cat-4(ok342)* V, outcrossed 3× to N2), LC81 (*cat-4(tm773)* V, outcrossed 5× to N2), LC143 (*pcbd-1(tm5924)* I, outcrossed 4× to N2), LC87 (*qdpr-1(tm2337)* III, outcrossed 2× to N2), MT15620 (*cat-2(n4547)* II, outcrossed 6× to N2), MT14984 (*tph-1(n4622)* II, outcrossed 6× to N2), LC144 (*agmo-1(e3016)* III, outcrossed 6× to N2), LC74 (*pah-1(ok687)* II, outcrossed 5× to N2), MQ887 (*isp-1(qm150)* IV, outcrossed 3× to N2), DA1116 (*eat-2(ad1116)* II, outcrossed 2× to N2), DA597 (*phm-2(ad597)* I, outcrossed 2× to N2), SJ4100 (*zcIs13[hsp-6p::GFP]*) V, outcrossed 3× to N2) and AM141 (*rmIs133[unc-54p::Q40::YFP]* X, outcrossed 5× to N2) strains were obtained from the *Caenorhabditis* Genetics Center at the University of Minnesota (CGC), which is supported by the NIH Office of Research Infrastructure Programs (P40 OD010440). CF1041 (*daf-2(e1370)* III) and CF512 (*fer-15(b26)* II*; fem-1(hc17)* IV) were provided by C. Kenyon. BJ49 (*kcIs6[ifb-2p::ifb-2a::CFP]* IV)^40^ was a gift from R. E. Leube. The AM23 (*rmIs298[F25B3.3p::Q19::CFP]*) and AM716 (*rmIs284[F25B3.3p::Q67::YFP]*) strains were provided by R. I. Morimoto^27^. MAH602 (*sqIs61[vha-6p::Q44::YFP + rol-6(su1006)]*) was gifted by M. Hansen^41^.

For tissue-specific RNAi experiments, we used either *rde-1*- or *sid-1*-mutant worms in which wild-type *rde-1* or *sid-1* genes were rescued using tissue-specific promoters, respectively. The strains WM118 (muscle RNAi, *rde-1(ne300)* V; *neIs9[myo-3p::HA::RDE-1* + *rol-6(su1006)]*)^42^, IG1846 (epidermis RNAi 1, *rde-1(ne300)* V; *frSi21[col-62p::rde-1 3*′*UTR]* II; *frIs7[nlp-29p::GFP *+ IV)^43^, NR222 (epidermis RNAi 2, *rde-1(ne219)* V; *kzIs9[lin-26p::NLS::GFP* + +* rol-6(su1006)]*)^44^, MAH677 (neuronal RNAi, *sid-1(qt9)*; *sqIs71 [rgef-1p::GFP *+* rgef-1p::sid-1]*^45^, IG1839 (intestine RNAi 1, *rde-1(ne300)* V; *frSi17[mtl-2p::rde-1 3UTR]* II; *frIs7 [nlp-29p::GFP *+* col-12p::DsRed]* IV)^43^, VP303 (intestine RNAi 2, *rde-1(ne219)* V; *kbIs7[nhx-2p::rde-1 *+* rol-6(su1006)]*)^46^, and DCL569 (germline RNAi, *mkcSi13[sun-1p::rde-1::sun-1 3*′*UTR *+* unc-119(*+*)]* II; *rde-1(mkc36)* V) were provided by the CGC. The RB891 strain (*ent-1(ok743)* IV) was also obtained from the CGC and outcrossed four times to N2 wild-type worms to generate DVG397. Then, DVG397 was crossed with MAH677 and WM118 to generate DVG399 and DVG400, respectively.

The control DVG9 strain (N2, *ocbEx9[myo3p::GFP]*) was generated by microinjecting 20 ng μl^−1^ pPD93_97 (*myo-3p::GFP*) into the gonads of adult N2 hermaphrodite animals^47^. To generate DVG348 (N2, *ocbEx280[sur-5p::cat-4, myo-3p::GFP]*), we injected a DNA mixture containing 70 ng μl^−1^ of the sur5-p::cat-4 plasmid and 5 ng μl^−1^ pPD93_97 (myo3-p::GFP). Following this method, we generated the strains DVG349 (intestine-specific overexpression: N2, *ocbEx281[gly-19p::cat-4, myo-3p::GFP]*), DVG351 (neuron-specific overexpression: N2, *ocbEx283[rgef-1p::cat-4, myo-3p::GFP]*), and DVG352 (muscle-specific overexpression: N2, *ocbEx284[myo-3p::cat-4, myo-3p::GFP]*). Likewise, we used the neuronal-specific RNAi strain (MAH677) to generate DVG377 (*sid-1(qt9)*;* sqIs71[rgef-1p::GFP *+ *rgef-1p::sid-1]*;* ocbEx301[myo-3p::cat-4, myo-3p::mCherry]*) and the corresponding control DVG378 (*sid-1(qt9)*; *sqIs71[rgef-1p::GFP *+* rgef-1p::sid-1]*;* ocbEx302[myo3p::mCherry]*). To generate DVG389 (*trpa-1(ok999)* IV; *ocbEx309[myo-3p::GFP]*) and DVG390 (*trpa-1(ok999)* IV;* ocbEx310[myo-3p::cat-4, myo-3p::GFP]*), we injected the gonads of TQ233 worms as indicated above.

To generate DVG373 (*rmIs298[F25B3.3p::Q19::CFP]*;* ocbEx297[myo-3p::cat-4, myo-3p::mCherry]*) and DVG375 (*rmIs284[F25B3.3p::Q67::YFP]*;* ocbEx299[myo-3p::cat-4, myo-3p::mCherry]*), we injected the gonads of AM23 and AM716 worms with a DNA mixture containing 100 ng μl^−1^ of the myo3-p::cat-4 plasmid and 5 ng μl^−1^ of pCFJ104 (myo-3p::mCherry). The corresponding control strains, DVG376 (*rmIs298[F25B3.3p::Q19::CFP]*; *ocbEx300[myo-3p::mCherry]*) and DVG380 (*rmIs284[F25B3.3p::Q67::YFP]*;* ocbEx304[myo-3p::mCherry]*), were generated by injecting 5 ng μl^−1^ of the pCFJ104 plasmid (myo-3p::mCherry) alone.

The VDL20/PHX10108 strain expressing endogenous CAT-4 tagged with GFP (*cat-4(syb10108)*) was generated by SunyBiotech (http://www.sunybiotech.com/) using the following sgRNAs: sg1, CACCACAACATCCTGCATGTTGG; and sg2, CCGTGACGATCCAAAGACTCGCG. Genome editing was confirmed by sequencing the *cat-4* gene fused to GFP (primers: 5′-CATTTAGTTCCATTTATGGG-3′ and 5′-TTTGGAGCTCGAGTCGCTTA-3′). From the VDL20 strain, we generated DVG387 (*cat-4::SL2::GFP*) by CRISPR–Cas9-mediated insertion^48^ of an SL2 sequence using the crRNA AAAGGAGAAGAACTTTTCAC, enabling independent translation of CAT-4 and GFP from a single bicistronic pre-mRNA transcribed under the endogenous *cat-4* promoter. The editing was confirmed using the following primers: 5′-CCGGAGTTGCAGTTGTTATTG-3′ and 5′-TCTCGAGAAGCATTGAACACC-3′.

For all *C. elegans* experiments, exact sample sizes are indicated in the corresponding figure legends. No statistical methods were used to predetermine sample size. Sample sizes were selected based on standards established in the field and were similar to or greater than those reported to be sufficient in previous studies using the same procedures (for example, lifespan, nose-touch, chemotaxis and motility assays)^2,10,28,49–52^. Worm populations were synchronized either by allowing young hermaphrodites to lay eggs for 6 h or by bleaching^53^. For synchronization by egg laying, young hermaphrodites were randomly selected from maintenance plates. For synchronization by bleaching, agar chunks containing mixed-stage animals were randomly selected from maintenance plates, and larvae were allowed to develop until sufficient young hermaphrodites were available for bleaching. After the 6 h egg-laying period or bleaching, larvae were raised to adulthood. For lifespan assays, adult worms were randomly selected from the synchronized population and allocated to the different experimental conditions. For all other experiments, adult worms were randomly distributed from single synchronized populations into the different experimental groups. Thus, allocation of worms to experimental conditions was randomized throughout the study. Samples from different experimental conditions were collected and lysed in a randomized order to minimize potential processing-order effects. Samples and experimental conditions were not processed in a blinded manner. To minimize potential bias and assess reproducibility, key experiments, including phenotypic assays, were independently repeated by at least two researchers involved in the study.

### Metabolomics

Adult worms were collected at the ages indicated in the corresponding figures. Worms were lysed in an extraction solution consisting of 50% LC–MS–grade methanol (Thermo Fisher Scientific, 10284580), 30% LC–MS–grade acetonitrile (Thermo Fisher Scientific, 10001334), 20% ultrapure water and 5 μM valine-d8 (CK Isotopes, DLM-488), using a volume equivalent to 10× the worm pellet weight. The worm pellet was transferred to a Precellys tube and homogenized using a Precellys Evolution Touch Homogenizer (Bertin Technologies). The lysates were then transferred to Eppendorf tubes and incubated for 15 min at 4 °C on a shaker at maximum speed. After incubation, the samples were centrifuged at the maximum speed for 25–40 min and the supernatant was collected.

The worm extracts were normalized by weight for metabolomics analysis of all possible metabolites. Metabolites were separated chromatographically using a Millipore SeQuant ZIC-pHILIC analytical column (5 µm, 2.1 × 150 mm) with a matching 2.1 × 20 mm guard column, both with 5 µm particle size. A binary solvent system was used, consisting of 20 mM ammonium carbonate with 0.05% ammonium hydroxide as solvent A and pure acetonitrile as solvent B. Chromatography was performed at 40 °C with a flow rate of 0.200 ml min^−1^, using the following gradient program: 80% solvent B from 0 to 2 min, a linear gradient to 20% solvent B from 2 to 17 min, a rapid return to 80% solvent B from 17 to 17.1 min, and a hold at 80% solvent B from 17.1 to 23 min. The autosampler tray was maintained at 4 °C, and 5 µl of each sample was injected. The samples were randomized to minimize analytical variability, and pooled quality-control samples, prepared by mixing equal volumes of all individual samples, were analysed regularly to ensure consistency.

Metabolite quantification was performed using a Vanquish Horizon UHPLC system coupled to the Orbitrap Exploris 240 mass spectrometer (Thermo Fisher Scientific) with a heated electrospray ionization (HESI) source. Ionization parameters were set to +3.5 kV (positive mode) and −2.8 kV (negative mode), with an RF lens value of 70, a heated capillary temperature of 320 °C and an auxiliary gas heater temperature of 280 °C. Sheath gas flow was set to 40, auxiliary gas to 15 and sweep gas was off. MS1 scans covered a mass range of *m*/*z* 70–900, with automatic gain control (AGC) targets and maximum injection times. Data were acquired in full-scan mode with polarity switching at a resolution of 120,000. The AcquireX Deep Scan workflow facilitated untargeted metabolite identification by iterative data-dependent acquisition. Fragmentation was conducted with resolutions of 60,000 for full scans and 30,000 for MS/MS, an intensity threshold of 5.0 × 10^3^, a 10 s dynamic exclusion, a 5 ppm mass tolerance, an isolation window of 1.2 *m*/*z* and stepped normalized collision energies of 30, 50 and 150. Mild trapping was enabled to improve signal quality.

Metabolite identification was performed using Compound Discoverer software (v.3.2, Thermo Fisher Scientific), with criteria including a precursor ion *m*/*z* within 5 ppm of the theoretical mass, fragment ions within 5 ppm of an internal spectral library, a minimum match score of 70 and retention times within 5% of purified standards analysed under identical conditions. For downstream analyses, we focused on annotated metabolites with spectra and retention times matching entries in our in-house library, which contains over 1,000 compound standards. Peak area integration and chromatogram review were carried out using TraceFinder software (v.5.0, Thermo Fisher Scientific). To correct any potential variations introduced from sample handling to instrument analysis, we normalized the detected metabolites in each sample using the probabilistic quotient normalization method^54^. The data were processed using the R package MetaProViz^55^ (https://github.com/saezlab/MetaProViz), with the only modification being that probabilistic quotient normalization was performed using 100% of the features from the multtest package (v.2.56.0)^56^, instead of total ion count normalization. Downstream statistical analyses were performed using GraphPad Prism (v.10.6.0). Data were analysed with two-sided unpaired *t*-tests, followed by the two-stage step-up method of Benjamini, Krieger and Yekutieli to correct for multiple comparisons.

For correlation analysis of metabolite changes between independent datasets (experiments 1 and 2), metabolites were filtered to retain metabolites, log2[fold change] and FDR-adjusted *P* (*q* value). The datasets were merged using an inner join on metabolite names. Significance was defined as an FDR-adjusted *P* < 0.01. Pearson correlation was calculated using only metabolites that were significant in both datasets. Scatter plots showing log_2_-transformed fold changes from experiments 1 and 2 were generated using the ggplot2 package (v.4.0.3) in R (v.4.5.2). Reference lines at zero (*x* and *y* axes) and a diagonal identity line (slope = 1) were included to aid interpretation. The Pearson correlation coefficient (*r*) and corresponding *P* value were annotated on the plot. Extreme outliers (for example, carnitine-C16) were excluded before visualization. For Venn diagram analysis, significantly upregulated metabolites were identified independently in each dataset using thresholds of fold change > 1 and FDR-adjusted *P* < 0.01. Venn diagrams were generated using the ggvenn package (v.0.1.19) in R (v.4.5.2). Dot plots comparing the 45 commonly and significantly upregulated metabolites (fold change > 1 and FDR-adjusted *P* < 0.01) across experiments 1 and 2 were generated using ggplot2 (v.4.0.3) in R (v.4.5.2).

### ELISA quantification of BH4 levels

We quantified BH4 levels using a BH4 ELISA Kit (Novus, NBP2-74983), according to the manufacturer’s instructions. Worms were lysed at day 5 of adulthood in protein lysis buffer (50 mM Tris-HCl, pH 7.8, 150 mM NaCl, 0.25% sodium deoxycholate, 1 mM EDTA and protease inhibitor (Roche)) using a Precellys 24 homogenizer. Worm lysates were centrifuged at 10,000 rpm for 10 min at 4 °C and the supernatant was collected. Protein concentrations were determined with standard BCA protein assay (Thermo Fisher Scientific). In total, 650 µg of total protein was used for measurement.

### Label-free quantitative proteomics

For comparison of protein abundance across temperatures (15 °C, 20 °C and 25 °C), synchronized day 5 adult worms were lysed in urea buffer (8 M urea, 50 mM triethylammonium bicarbonate (TEAB)) by glass-bead homogenization and cleared by centrifugation (13,000 rpm, 10 min). The supernatants were reduced with 5 mM dithiothreitol (DTT) for 1 h, alkylated with 40 mM chloroacetamide for 30 min and digested with trypsin (1:75 ratio, w/w) after dilution of urea to 2 M with 50 mM TEAB. For tyrosine-treatment experiments, day 3 adult worms were lysed in urea buffer as indicated above. The supernatants were reduced with 10 mM DTT for 30 min, alkylated with 40 mM chloroacetamide for 30 min and digested with Lysyl endopeptidase (Lys-C, 1:100 ratio) at 25 °C for 2 h. Proteins were then further digested with trypsin (1:50 ratio) after dilution of urea to 2 M with 50 mM TEAB. One day later, the samples were acidified with formic acid (1% final concentration), cleared by centrifugation (16,000 *g* for 5 min), purified using StageTips and subjected to mass spectrometry (MS) analysis.

MS was performed at the CECAD Proteomics Facility. Temperature-comparison samples were analysed on the Orbitrap Exploris 480 mass spectrometer equipped with a FAIMSpro differential ion mobility device coupled to an UltiMate 3000 (Thermo Fisher Scientific). Tyrosine-treatment samples were analysed on the Orbitrap Eclipse coupled to a Vanquish neo system (Thermo Fisher Scientific). The samples were loaded onto a precolumn (Acclaim 5 µm PepMap 300 µ Cartridge or Ionopticks NanoShield, respectively) before being reverse-flushed onto an in-house-packed analytical column (30 cm length, 75 µm inner diameter, filled with 2.7 µm Poroshell EC120 C18, Agilent) or an Aurora Ultimate 25 cm column (Ionopticks), respectively. Peptides were chromatographically separated at a constant flow rate of 300 nl min^−1^ on a 90 min linear gradient running 0.1% formic acid against increasing amounts of 80% acetonitrile with 0.1% formic acid. For measurements where FAIMS pro was used, it was operated at constant −50 V compensation voltage and electrode temperatures of 99.5 °C for the inner and 85 °C for the outer electrode.

For the gas-phase fractionated library^57^, a pool generated from all samples was analysed in six individual runs covering the range from 400 *m*/*z* to 1,000 *m*/*z* in 100 *m*/*z* increments. For each run, MS1 was acquired at 60,000 resolution with a maximum injection time of 98 ms and an AGC target of 100%. MS2 spectra were acquired at 30,000 resolution with a maximum injection time of 60 ms. Spectra were acquired in staggered 4 *m*/*z* windows, resulting in nominal 2 *m*/*z* windows after deconvolution using ProteoWizard^58^.

For each sample acquired on the Orbitrap Exploris 480, MS1 scans were obtained from 399 *m*/*z* to 1,001 *m*/*z* at 15,000 resolution. The maximum injection time was set to 22 ms and the AGC target was set to 100%. MS2 scans ranged from 400 *m*/*z* to 1,000 *m*/*z* and were acquired at 15,000 resolution with a maximum injection time of 22 ms and an AGC target of 100%. DIA scans covering the precursor range from 400 to 1,000 *m*/*z* and were acquired in 60 × 10 *m*/*z* windows with an overlap of 1 *m*/*z*. All of the scans were stored as a centroid. For samples acquired on Orbitrap Eclipse, the MS1 and MS2 resolution was set to 30,000 with a maximum injection time of 54 ms and identical AGC targets. The same range of 400 to 1,000 *m*/*z* was covered using 20 windows of 30 *m*/*z* each.

For samples acquired on the Orbitrap Exploris 480 (temperature-comparison datasets), the gas-phase fractionated library was build using DIA-NN (v.1.8.1)^59^ and the *C. elegans* proteomic database from WormBase (PRJNA13758.WBPS15, downloaded on 27 July 2021), with settings matching acquisition parameters. The samples were analysed in DIA-NN v.1.8.1 as well using the previously generated library and identical database. DIA-NN was run with the additional command line prompts ‘--report-lib-info’ and ‘--relaxed-prot-inf’. Further output settings were: filtered at 0.01 FDR, N-terminal methionine excision enabled, maximum number of missed cleavages set to 1, minimum peptide length set to 7, maximum peptide length set to 30, minimum precursor *m*/*z* set to 400, maximum precursor *m*/*z* set to 1,000 and cysteine carbamidomethylation enabled as a fixed modification. Next, the DIA-NN output was further filtered on a library *q* value and global *q* value of ≤ 0.01 and at least two unique peptides per protein using R (v.4.1.3). Then, label-free quantification values were calculated using the DIA-NN R package. The results were analysed using Perseus (v.1.6.15)^60^. Proteins were filtered for data completeness in at least one replicate group and missing values imputed using sigma downshift approach with standard settings. Next, FDR-controlled two-sided *t*-tests were performed between the experimental groups and WormBase IDs mapped to UniProt IDs.

For samples acquired on Orbitrap Eclipse (tyrosine-treatment datasets), a directDIA approach using Spectronaut 20.5 (Biognosys) was used with the GPF runs added as supporting runs and searched against the WormBase *C. elegans* proteomic database (PRJNA13758.WBPS19, downloaded 4 January 2025). The Spectronaut output was imported into R (v.4.5.2) for downstream analysis. Protein groups were expanded to individual UniProt identifiers, mapped to gene symbols using the *C. elegans* annotation database (org.Ce.eg.db, v.3.22.0), and protein abundances were summarized at the gene level using median values. Proteins with >50% missing values across samples were excluded. Protein intensities were log_2_-transformed after addition of a pseudocount of 1. Differential protein abundance analysis was conducted using the limma package (v.3.66.0) with two-sided empirical Bayes-moderated *t*-test. Proteins were considered differentially abundant when they exhibited an absolute log_2_-transformed fold change of >0.58 (corresponding to a 1.5-fold change) and a nominal *P* value < 0.05.

### ORA

ORA was performed using the enrichGO function from the clusterProfiler package in R (v.4.5.2) to identify Gene Ontology (GO) terms enriched among differentially abundant proteins. Analyses were conducted using the org.Ce.eg.db annotation database (v.3.22.0). GO Biological Process and Molecular Function ontologies were analysed using a one-sided hypergeometric test. Multiple-testing correction was performed using the Benjamini–Hochberg method, with significance thresholds of *P* < 0.05 and *q* < 0.2. For visualization, enrichment results were converted to gene ratios (GeneRatio), representing the proportion of input genes associated with each GO term. Dot plots were generated using ggplot2 (v.4.0.3), with the *x* axis representing the gene ratio, the *y* axis indicating GO terms, point size corresponding to the number of genes associated with each term (Count) and point colour representing statistical significance (−log_10_[*P*]).

### Analysis of transcriptomic data

The temperature-dependent gene expression data from day 3 adult *C. elegans* at 15 °C and 25 °C were obtained from the NCBI Gene Expression Omnibus (GEO) database under accession number GSE62297 (ref. ^12^). Raw data were reanalysed using the database’s in-built GEO2R web tool with default parameters, including multiple-testing correction using the Benjamini–Hochberg method to calculate FDR-adjusted *P* values.

### qPCR

Total RNA was isolated from synchronized day-5 adult worms using RNAbee (Tel-Test, CS501-B). We then generated cDNA from isolated RNA using the qScript Flex cDNA synthesis kit (Quantabio). Quantitative PCR (qPCR) assays were performed with SYBR Green and a 1:20 dilution of cDNA on the CFX384 Real-Time System (Bio-Rad). Gene expression levels were normalized to the geometric mean of *cdc-42* and *Y45F10D.4* (ref. ^61^). Data were analysed using the comparative ${2}^{\varDelta \varDelta {C}_{{\rm{t}}}}$ method, which provides relative changes in gene expression to the control condition after correction for housekeeping genes^62^. Details about the primers used for qPCR are provided in Supplementary Table 8.

### Imaging and quantification of the CAT-4 reporter

For imaging of the DVG387 (*cat-4p::cat-4::SL2::GFP*) reporter strain or AID-strains (*cat-4::3×Flag::mIAA7::GFP*), day 5 adult worms were immobilized with 0.1% sodium azide in M9 buffer and mounted on glass slides under coverslips. Fluorescence images of whole worms were acquired using the Zeiss Axio Zoom.V16 microscope. For quantification of GFP intensity, the anterior region of the intestine was outlined and quantified using ImageJ (v.1.54p). For muscle regions, higher-magnification images were acquired using the Zeiss ApoTome Axio Imager Z.1 fluorescence microscope.

### RNAi constructs

Hermaphrodite worms were fed HT115 *E. coli* carrying either an empty control vector (L4440) or vectors expressing double-stranded RNAi. The RNAi constructs targeting *cat-4*, *ent-1*, *ent-6* and *hsf-1* were obtained from the Ahringer library. The *mdt-15*, *Y39G8B.1*, *dhs-31*, *dhfr-1*, *qdpr-1*, *pcbd-1*, *ent-4*, *ent-5*, *cco-1*, *agmo-1*, *cat-2*, *pah-1*, *tph-1*, *hpd-1*, *cct-2* and *fkb-6* RNAi constructs were obtained from the Vidal library. The *daf-16* and *psme-3* RNAi constructs were generated in previous studies^2,63^. All RNAi constructs were sequence verified. A list of the RNAi sequences is provided in Supplementary Table 8.

### AID

Genome editing was performed using a CRISPR protocol as previously described^48^. The strain PP4642, *cat-4(hh131[cat-4::3×Flag::mIAA7::gfp]* V, was generated by inserting a *C. elegans* codon-optimized *3×Flag::mIAA7* sequence in-frame immediately upstream of the *GFP* sequence in the strain VDL20 *cat-4(syb10108[cat-4::GFP])* V. The strain PP4640, wrdSi58[eft-3p::TIR1(F79G)::F2A::mTagBFP2::AID*::NLS::tbb-2 3′*UTR]* II, was generated by introducing the F79G mutation into TIR1 in strain JDW313. Similarly, PP4641 (reSi11[unc-54p::TIR1(F79G)::F2A::mTagBFP2::AID*::NLS::tbb-2 3′*UTR]* II) was generated by mutating TIR1(F79G) using DV3825 strain. All genomic modifications were confirmed by Sanger sequencing. A list of the oligonucleotide sequences used for strain generation and sequencing is provided in Supplementary Table 8.

PP4640, PP4641 and PP4642 were each outcrossed five times to N2 wild-type worms. The strains PP4634 (*cat-4(hh131[cat-4::3×Flag::mIAA7::GFP]* V;wrdSi58[eft-3p::TIR1(F79G)::F2A::mTagBFP2::AID*::NLS::tbb-2 3′*UTR]* II) and PP4635 (*cat-4(hh131[cat-4::3×Flag::mIAA7::GFP]* V; reSi11[unc-54p::TIR1(F79G)::F2A::mTagBFP2::AID*::NLS::tbb-2 3′*UTR]* II) were generated by crossing PP4642 with PP4640 and PP4641, respectively.

AID experiments were performed as described previously^64^ with minor modifications. To induce CAT-4 degradation, age-synchronized animals were transferred to *E. coli* OP50-seeded NGM plates supplemented with 10 µM auxin (5-Ph-IAA, Biozol, T8885) at the ages indicated in the corresponding figures and maintained on auxin-containing plates until the end of the experiment.

### Construction of *cat-4 C. elegans* expression plasmids

To construct the *C. elegans* plasmid for ubiquitous somatic overexpression of *cat-4*, pPD95.77 from the Fire Lab kit (Addgene) was digested with SphI and XmaI to insert 3.6 kb of the *sur-5* promoter. The resulting vector was then digested with KpnI and EcoRI to excise GFP and insert a multicloning site. *F32G8.6* (*cat-4*) was PCR amplified from cDNA to include 5′ XbaI and 3′ KpnI restriction sites and subsequently cloned into the vector described above. To generate the neuron-specific overexpression *cat-4* plasmid, the pMS1 plasmid (rgef-1p::rpn-6.1::unc-54 3′UTR) containing an inserted multicloning site^10^ was digested with 5′ KpnI and 3′ApaI restriction sites. Then, *cat-4* was PCR-amplified from cDNA to include 5′ KpnI and 3′ ApaI restriction sites and cloned into pMS1. Using the same strategy, *cat-4* was cloned into pMS2 (*gly-19p::rpn-6.1::unc-54* 3′ UTR)^10^ to generate intestine-specific expression plasmid. For the muscle-specific expression plasmid, the pMS3 plasmid (myo-3p::rpn-6.1::unc-54 3′ UTR)^10^ was digested with 5′ XbaI and 3′ AscI restriction sites. All constructs were sequence-verified.

### Lifespan assay

Synchronized larvae obtained by an egg laying protocol were raised on OP50 *E. coli* at 20 °C until day 1 of adulthood. Once hermaphrodite worms reached adulthood, they were shifted to a given temperature (15 °C, 20 °C or 25 °C) on plates seeded with HT115 *E. coli* carrying either empty vector or RNAi constructs. When indicated in the corresponding figure legends, RNAi treatment was initiated from hatching. For lifespan experiments with mutant and AID worms, the animals were fed OP50 *E. coli* throughout adulthood. Adult worms (*n* = 96 per condition) were scored every day or every other day^49^. From the initial population, worms that were lost, burrowed into the medium, or exhibited a protruding vulva or bagging phenotype were censored. We used GraphPad Prism (v.10.6.0) and OASIS (v.2)^65^ to determine median and mean lifespan, respectively. The *P* values were calculated with GraphPad Prism (v.10.6.0) using the log-rank (Mantel–Cox) method and refer to comparisons between experimental and control animals within a single lifespan experiment. Each lifespan graph represents a single, representative experiment. Supplementary Table 7 contains the number of total/censored worms and detailed statistical analyses for each replicate lifespan experiment.

### Motility assay

*C. elegans* were synchronized on OP50 *E. coli* using the egg-laying method and grown to day 1 of adulthood, then randomly transferred to plates seeded with OP50 and vehicle control (DMSO) or BH2. At the ages indicated in the corresponding figures, worms were randomly picked and placed in 20 μl of M9 buffer on a microscope slide. After 30 s of adaptation, the number of body bends (that is, change in direction of the bend at the mid-body) was counted for 30 s^27,66^.

### Brood size

Wild-type worms were maintained on standard NGM plates seeded with *E. coli* OP50 until the late L4 stage. Animals were then transferred to plates supplemented with 500 µM BH2 or DMSO vehicle control. Three worms were placed onto each experimental plate and transferred daily to freshly prepared treatment plates until egg laying ceased. The numbers of viable progeny and unhatched eggs were scored every 24 h. Brood size was calculated as the total number of progeny produced per worm throughout the reproductive period.

### Bacterial colonization assay

Bacterial invasion assays were performed as previously described^21,22^. HT115 *E. coli* cultures were grown overnight at 37 °C, induced with 1 mM IPTG for 4 h, collected and concentrated tenfold by centrifugation. Concentrated HT115 bacteria carrying the empty vector plasmid were mixed at a 4:1 ratio with concentrated HT115 bacteria expressing mCherry from the pDP151 plasmid. NGM agar plates were seeded with the bacterial mixture and allowed to dry at room temperature for 24 h. L4 larvae synchronized by an egg-laying protocol were randomly transferred onto the seeded plates and maintained under the indicated experimental conditions. Worms were analysed by fluorescence microscopy at day 1 and day 10 of adulthood. Before imaging, worms were gently collected from plates, washed three times with M9 buffer and transferred to OP50 *E. coli* plates for 2 h to eliminate residual fluorescent bacteria from the intestinal lumen. Worms were then immobilized in 0.1% sodium azide in M9 buffer on 2% agarose pads. Fluorescence images were acquired using the Zeiss Axio Zoom.V16 fluorescence microscope. For quantification of fluorescence signal, worms were outlined and quantified using ImageJ software (v.1.51s).

### Chemotaxis assay

After development at 20 °C, synchronized adult worms were either kept at 20 °C or transferred to 15 °C until the ages specified in the corresponding figures. Chemotaxis tests were performed on freshly poured agar plates (2% agar, 5 mM KPO_4_ at pH 6.0, 1 mM CaCl_2_, 1 mM MgSO_4_). The plates were sectioned into four quadrants, along with an inner circle measuring approximately 1 cm across diagonally. A benzaldehyde attractant (0.5% benzaldehyde in ethanol supplemented with 0.25 M sodium azide) was applied to one pair of diagonal quadrants, while the opposite quadrants received a control mixture (ethanol + 0.25 M sodium azide). On the specified adult days (as indicated in figure legends), worms were collected in S-Basal medium, washed three times to remove residual bacteria and deposited at the centre of the plate. After sealing with Parafilm, the plates were incubated at the indicated temperatures for 90 min. Worms were then counted in each quadrant, excluding those remaining within the central circle. The chemotaxis index was calculated using the following formula: (number of animal in attractant quadrants − number of animals in control quadrants)/total number of animals (ref. ^67^).

### Nose-touch assay

Age-synchronized worms were assessed for nose-touch response according to established protocols^28,50,68,69^. In brief, the worms were placed onto a thin lawn of bacteria and allowed to move forward. A fine eyelash tool was gently positioned in the path of each moving worm. Animals that failed to reverse and instead continued crawling over or under the eyelash were scored as non-responsive. For each experimental group, 40 worms were tested, and their responses to 10 gentle eyelash touches were recorded.

### BH2 and tyrosine supplementation

For BH2 supplementation, synchronized day 1 adult worms were collected and randomly distributed onto OP50 *E. coli*-seeded plates containing either BH2 (MedChemExpress, HY-W008646) or vehicle control (DMSO). For tyrosine supplementation, synchronized animals were placed on plates seeded with OP50 (or HT115 for RNAi experiments) and supplied with either tyrosine (Sigma-Aldrich, T8566) or the corresponding vehicle control (HCl). BH2 or tyrosine treatments were applied starting on day 1 of adulthood and continued until sample collection or maintained throughout adulthood for lifespan assays. Human cells were treated for 3 days with BH2 or tyrosine at the concentrations specified in the corresponding figures.

### Human cell lines

HEK293T/17 cells (American Type Culture Collection (ATCC), CRL-11268) were plated onto 0.1% gelatin-coated plates and maintained in DMEM (Thermo Fisher Scientific, 11966025), supplemented with 10% FBS (Thermo Fisher Scientific, 10500064), 1% GlutaMAX (Life Technologies, 35050038) and 1% MEM non-essential amino acids (Thermo Fisher Scientific, 11140035) under standard conditions (37 °C, 5% CO_2_). Isogenic control iPS cells (*FUS*^*WT/WT*^) and ALS iPS cells (*FUS*^*P525L/P525L*^) were provided by I. Bozzoni and A. Rosa. Both iPS cell lines were established and characterized for pluripotency in ref. ^70^. In brief, control iPS cells were derived from a healthy donor and verified not to contain mutations in *FUS*. ALS iPS cells were generated from control iPS cells using transcription activator-like effector nuclease (TALEN)-directed mutagenesis and carry the homozygous *FUS*^*P525L*^ mutation associated with severe ALS^70^. The identity of both iPS cell lines was authenticated in our laboratory by short-tandem-repeat profiling in a previous publication^71^. The iPS cell lines used in the present study were thawed directly from frozen stocks of these authenticated lines. iPS cells were cultured on Geltrex (Thermo Fisher Scientific, A1413302) in mTeSR1 medium (StemCell Technologies, 85850) at 37 °C with 5% CO_2_. Undifferentiated iPS cell colonies were passaged using 2 mg ml^−1^ dispase (StemCell Technologies, 07913), followed by mechanical dissociation with a glass pipette. All of the cell lines were routinely tested for mycoplasma contamination, and no contamination was detected.

### Motor neuron differentiation

Motor neurons were derived from iPS cell lines according to a monolayer-based differentiation protocol^72^. iPS cells were seeded on Geltrex-coated plates and cultured in mTeSR1 medium until confluent. We then initiated the differentiation induction with neuron differentiation medium composed of DMEM/F12 and Neurobasal (1:1; Thermo Fisher Scientific, 11330057 and 21103049), supplemented with GlutaMAX (Thermo Fisher Scientific, 35050038), non-essential amino acids, B27 (Thermo Fisher Scientific, 12587010) and N2 (Thermo Fisher Scientific, 17502048). From day 0 to day 6, the medium was further supplemented with 1 μM retinoic acid (Sigma-Aldrich, R2625), 1 μM smoothened agonist (SAG; Sigma-Aldrich, 566661), 10 μM SB-431542 (Miltenyi Biotec, 130-105-336) and 0.1 μM LDN-193189 (Miltenyi Biotec, 130-103-925). From day 7 to day 14, neuron differentiation media were supplemented with 1 μM retinoic acid, 1 μM SAG, 4 μM SU-5402 (Sigma-Aldrich, SML0443) and 5 μM DAPT (Sigma-Aldrich, D5942). After day 14, differentiated motor neurons were dissociated and replated on poly-l-ornithine (Sigma-Aldrich, P3655) and laminin-coated (Thermo Fisher Scientific, 23017015) plates in Neurobasal medium, supplemented with non-essential amino acids, GlutaMAX, N2, B27 and neurotrophic factors (10 ng ml^−1^ BDNF (BIOZOL, 450-02) and 10 ng ml^−1^ GDNF (BIOZOL, 450-10)).

### Transfection of HEK293 human cells

HEK293T/17 cells (ATCC, CRL-11268) were seeded onto plates coated with 0.1% gelatin. Once cultures reached approximately 40% confluence, the cells were transfected with 1 μg of one of the following plasmids using FuGENE HD (Promega), according to the manufacturer’s protocol: CMV:mRFP-Q74^33,34^, pLVX-Puro-TDP-43-WT, pLVX-Puro-TDP-43-A382T, pcDNA3.1-FUS-HA-WT, or pcDNA3.1-FUS-HA-P525L. The cells were then treated with 10 µM BH2 or vehicle control (DMSO) and lysed after 72 h of incubation in standard growth medium. The FUS-HA plasmids were provided by D. Dormann^73^, and the pLVX-Puro-TDP-43 vectors were provided by S. Ferguson (Addgene, 133753 and 133756)^74^.

### Lentiviral infection of HEK293 human cells

Lentivirus (LV) non-targeting short hairpin RNA (shRNA) and LV *CCT2* shRNA (TRCN0000029499)^30^ in the pLKO.1-puro backbone were obtained from Mission shRNA (Sigma-Aldrich). The target sequence of the *CCT2* shRNA construct, previously validated in ref. ^30^, is provided in Supplementary Table 8. To generate stable shRNA-expressing HEK293 cell lines, cells were transduced with 5 µl of concentrated lentivirus and selected with 2 µg ml^−1^ puromycin (Thermo Fisher Scientific, A1113803).

### Immunocytochemistry

Cells were fixed with 4% paraformaldehyde in PBS for 20 min, followed by permeabilization with 0.2% Triton X-100 in PBS for 10 min and blocking with 3% BSA in 0.2% Triton X-100 in PBS for 10 min. Cells were then incubated with anti-MAP2 (Sigma-Aldrich, M1406, 1:300) and rabbit anti-cleaved caspase-3 (Cell Signaling Technology, 9661S, 1:300) for 2 h at room temperature. After washing with PBS, the cells were incubated with secondary antibodies (Alexa Fluor 488 goat anti-mouse (Thermo Fisher Scientific, A-11029, 1:500) and Alexa Fluor 568 F(ab′)2 fragment of goat anti-rabbit IgG (H+L) (Thermo Fisher Scientific, A-21069, 1:500)) together with Hoechst 33342 (Life Technologies, 1656104) for 1 h at room temperature. The coverslips were then rinsed in PBS, followed by a distilled water wash, and mounted onto microscope slides using FluorSave Reagent (Merck, 345789).

### Filter trap and western blotting

For filter trap and corresponding western blot assays, age-synchronized adult *C. elegans* were collected, washed with M9 buffer and snap-frozen in liquid nitrogen. The frozen pellets were thawed on ice and lysed in a non-denaturing buffer (50 mM HEPES (pH 7.4), 150 mM NaCl, 1 mM EDTA, 1% Triton X-100, 2 mM sodium orthovanadate, 1 mM PMSF, protease inhibitor cocktail (Roche)) using a Precellys 24 homogenizer. Lysates were cleared of worm debris by centrifugation (8,000 *g*, 5 min, 4 °C) and protein concentrations were measured using the BCA assay (Thermo Fisher Scientific). For western blot analysis, 30 μg of total protein was resolved by SDS–PAGE and transferred onto PVDF membranes (Millipore). For filter trap analysis, 100 μg of protein was supplemented with SDS to a final concentration of 0.5% SDS and loaded to a cellulose acetate membrane mounted in a slot-blot apparatus (Bio-Rad). After washing with 0.2% SDS, SDS-resistant aggregates were detected by immunoblotting. For immunoblotting analysis in *C. elegans*, including both filter trap and western blot assays, we used anti-IFB-2 (Developmental Studies Hybridoma Bank, MH33, 1:1,000) and anti-GFP (AMSBIO, TP401, 1:5,000) antibodies. Western blots were additionally probed using antibody against α-tubulin loading control (Sigma-Aldrich, T6199, 1:5,000).

For analysis of CAT-4 levels in AID strains, synchronized populations of *C. elegans* grown on *E. coli* OP50 were collected at late-L4/young adult stage after auxin treatment, washed three times with M9 buffer and snap-frozen in 1× SDS sample buffer supplemented with DTT. The samples were then thawed, heated at 90 °C for 5 min, dissociated using a Precellys homogenizer and centrifuged at 15,000 rpm for 15 min at 22 °C. The supernatants were collected and proteins were separated on 5–10% Bis-Tris gels before transfer to a nitrocellulose membrane (Amersham Protran, Cytiva). Membranes were blocked for 1 h at room temperature with 1× ROTI Block in TBS, and incubated for 1 h at room temperature with anti-Flag M2-HRP mouse (Sigma-Aldrich, A8592, 1:10,000) or anti-α-tubulin mouse (Sigma-Aldrich, T6074, 1:5,000). Membranes probed with anti-α-tubulin were incubated with goat anti-mouse IRDye 800CW secondary antibody (Li-Cor, 926-322101:10,000) for 1 h at room temperature. After three washes with TBS-T, membranes were imaged using a ChemiDoc Imaging System (Model MP, Bio-Rad).

For filter trap and western blot analyses of HEK293 cells, the cells were lysed in buffer containing 50 mM HEPES (pH 7.4), 150 mM NaCl, 1 mM EDTA, 1% Triton X-100, 2 mM sodium orthovanadate, 1 mM PMSF and protease inhibitors, followed by homogenization through a 27-gauge needle. Lysates from cells expressing mRFP-Q74 were cleared at 8,000*g* for 5 min at 4 °C. Lysates from cells expressing FUS-HA-WT, FUS-HA-P525L, pLVX-Puro-TDP-43-WT or pLVX-Puro-TDP-43-A382T were centrifuged at 1,000*g* for 5 min at 4 °C. Protein concentrations were determined by BCA assay. For western blotting, 30 μg of protein was analysed. For filter trap analyses, 100 μg of total protein was brought to 0.5% SDS and loaded onto cellulose acetate membranes as described above, followed by washing in 0.2% SDS and immunoblot detection of SDS-resistant aggregates. For immunoblotting in filter trap and western blot experiments, HEK293 lysates were probed using antibodies against mCherry (Abcam, ab167453, 1:5,000), FUS (Abcam, ab154141, CL0190, 1:1,000), TDP-43 (Abcam, ab225710, 1:1,000), anti-LC3B (Sigma-Aldrich, L7543, 1:1,000), anti-CCT2 (Abcam, ab92746, 1:1,000) and β-actin (Abcam, 8226, 1:5,000).

For necroptosis analysis, iPS-cell-derived motor neurons were lysed in RIPA buffer (50 mM Tris-HCl (pH 7.4), 150 mM NaCl, 1% Triton X-100, 0.1% SDS, 1% sodium deoxycholate, 1 mM PMSF, 1 mM EDTA and protease inhibitor cocktail). Immunoblotting was performed with anti-phospho-RIP (Ser166) (Cell Signaling Technology, 65746, D1L3S, 1:1,000), anti-RIP (Cell Signaling Technology, 3493, D94C12, 1:1,000) and β-actin (Abcam, 8226, 1:5,000) antibodies.

Densitometry of filter trap and western blot experiments was performed using ImageJ (v.1.51s). For quantification of filter trap and western blot data, we present the results as relative changes compared with the corresponding control conditions. To average and statistically analyse independent experiments for these assays, we normalized test conditions to their corresponding control groups measured concurrently in each replicate experiment. Statistical analysis was performed using GraphPad Prism (v.10.6.0). Gel source data are provided in Supplementary Fig. 1.

### MitoSOX staining

HEK293 cells were incubated with 1 μM MitoSOX Red superoxide indicator (Invitrogen, M36008) and Hoechst 33342 (Life Technologies, 1656104) in HBSS containing calcium and magnesium for 30 min at 37 °C in the dark. After incubation, cells were washed three times with HBSS containing calcium and magnesium, mounted onto microscope slides and imaged for subsequent analysis.

### Proteasome activity

*C. elegans* were lysed in proteasome activity assay buffer (50 mM Tris-HCl, pH 7.5, 10% glycerol, 0.5 mM EDTA, 5 mM MgCl_2_, 2 mM ATP and 1 mM dithiothreitol) using a Precellys 24 homogenizer (Bertin Technologies). Human cells were collected in the same buffer and lysed by passage through a 27-gauge needle attached to a 1 ml syringe 10 times. Lysates were centrifuged at 10,000*g* for 10 min at 4 °C. For each sample, 25 μg of total protein was transferred to a 96-well microplate (BD Falcon) and incubated with the fluorogenic trypsin-like proteasome substrate Ac-Arg-Leu-Arg-AMC (Enzo, BWL-AW9785-0005). Fluorescence generated by substrate cleavage (excitation, 380 nm; emission, 460 nm) was monitored using a microplate fluorometer (EnSpire, PerkinElmer) every 5 min for 2 h (worm lysates) or 1 h (human cell lysates). Proteasome activity was quantified as the slope of fluorescence accumulation over time. For comparisons across independent experiments, slope values were normalized to the corresponding control condition within each experiment before statistical analysis.

### Reporting summary

Further information on research design is available in the Nature Portfolio Reporting Summary linked to this article.

## Online content

Any methods, additional references, Nature Portfolio reporting summaries, source data, extended data, supplementary information, acknowledgements, peer review information; details of author contributions and competing interests; and statements of data and code availability are available at https://doi.org/10.1038/s41586-026-11152-9.

## Supplementary information

Supplementary Figure Supplementary Fig. 1: gel electrophoresis source data.

Reporting Summary

Supplementary Table 1 Metabolomics of *C. elegans* exposed at different temperatures during adulthood. Quantitative analysis of metabolite levels in day 5 adult wild-type N2 worms and *trpa-1(ok999)* worms exposed at different temperatures after development (*n* = 5 biological replicates). Statistical analysis: two-stage step-up method of Benjamini, Krieger and Yekutieli multiple *t*-test; FDR (*q* value) < 0.01 was considered significant.

Supplementary Table 2 Second independent metabolomics experiment of *C. elegans* exposed at different temperatures during adulthood. Quantitative analysis (second experiment) of metabolite levels in day 5 adult wild-type N2 worms and *trpa-1(ok999)* worms exposed at different temperatures after development (*n* = 5 biological replicates). Statistical analysis: two-stage step-up method of Benjamini, Krieger and Yekutieli multiple *t*-test; FDR (*q* value) < 0.01 was considered significant.

Supplementary Table 3 Metabolomics of *C. elegans* at different ages. Quantitative analysis of wild-type worms at day 1, 10 and 15 of adulthood, cultured at 15 °C or 20 °C after development (*n *= 5 biological replicates). Statistical analysis: two-stage step-up method of Benjamini, Krieger and Yekutieli multiple *t*-test; FDR (*q* value) < 0.01 was considered significant.

Supplementary Table 4 Metabolomics analysis of wild-type and *daf-2(e1370)* mutant *C. elegans* at different ages, maintained at 20 °C during the whole experiment. Quantitative analysis of wild-type and *daf-2(e1370)* mutant worms at day 1, 5, 10 and 15 of adulthood, cultured at 20 °C (*n* = 5–6 biological replicates). Statistical analysis: two-stage step-up method of Benjamini, Krieger and Yekutieli multiple *t*-test; FDR (*q* value) < 0.01 was considered significant.

Supplementary Table 5 Shotgun label-free proteomics of *C. elegans* exposed at different temperatures during adulthood. Proteomics of day 5 wild-type worms at 15 °C, 20 °C and 25 °C (*n* = 3 biological replicates; two-sided *t*-test; FDR < 0.05 was considered significant).

Supplementary Table 6 Shotgun label-free proteomics in *C. elegans* treated with tyrosine. Proteomics analysis of day 3 wild-type worms treated with 500 µM tyrosine or vehicle control (HCl) at 15 °C or 20 °C (*n* = 4–5 biological replicates per condition). Limma-based differential protein abundance analysis using two-sided empirical Bayes-moderated *t*-tests.

Supplementary Table 7 Statistical analysis and replicate data of lifespan experiments. Median lifespan, mean ± s.e.m. lifespan, statistical analyses and replicate data of all the lifespan experiments.

Supplementary Table 8 Sequences of RNAi/shRNA constructs. Sequences of RNAi constructs used for knockdown experiments in *C. elegans*, shRNA constructs used for human cell experiments, oligonucleotides used for AID strain generation and sequencing, and primers used for qPCR experiments.

Peer Review File

## Source data

Source Data Fig. 1

Source Data Fig. 3

Source Data Fig. 5

Source Data Extended Data Fig. 2

Source Data Extended Data Fig. 5

Source Data Extended Data Fig. 7

Source Data Extended Data Fig. 8

Source Data Extended Data Fig. 10

Source Data Extended Data Fig. 11

Source Data Extended Data Fig. 12

## Data Availability

All data supporting the findings of this study are available within the Article and its Supplementary Information. Proteomics data have been deposited in the ProteomeXchange Consortium through the PRIDE partner repository under dataset identifiers PXD059883 (*C. elegans* at different temperatures) and PXD079702 (tyrosine supplementation in *C. elegans*). Metabolomics data have been deposited in the NIH Common Fund’s National Metabolomics Data Repository (NMDR), the Metabolomics Workbench (https://www.metabolomicsworkbench.org)^75^, under study IDs ST004426 (cold-induced metabolites in day 5 adult worms, experiment 1), ST004433 (cold-induced metabolites in day 5 adult worms, experiment 2), ST004434 (cold-induced metabolites in day 1, 10 and 15 adult worms) and ST004437 (metabolomics analysis in wild-type and long-lived *daf-2* mutant *C. elegans* at different ages). These data can be accessed directly via the project (https://doi.org/10.21228/M84530). The whole-genome expression profiling of *C. elegans* adults was obtained from the NCBI GEO database under accession number GSE62297 (ref. ^12^). The *C. elegans* annotation database (org.Ce.eg.db, version 3.22.0) was obtained from Bioconductor (https://bioconductor.org/packages/release/data/annotation/html/org.Ce.eg.db.html). Source data are provided with this paper.

## References

[1] Conti, B. & de Cabo, R. Promoting health and survival through lowered body temperature. *Nat. Aging* **5**, 740–749 10.1038/s43587-025-00850-0 (2025). PMC13171288 40205073

[2] Lee, H. J. et al. Cold temperature extends longevity and prevents disease-related protein aggregation through PA28γ-induced proteasomes. *Nat. Aging* **3**, 546–566 10.1038/s43587-023-00383-4 (2023). PMC10191861 37118550

[3] Zhao, Z. J. et al. Body temperature is a more important modulator of lifespan than metabolic rate in two small mammals. *Nat. Metab.* **4**, 320–326 10.1038/s42255-022-00545-5 (2022). 35288719

[4] Conti, B. et al. Transgenic mice with a reduced core body temperature have an increased life span. *Science* **314**, 825–828 10.1126/science.1132191 (2006). 17082459

[5] Hosono, R., Mitsui, Y., Sato, Y., Aizawa, S. & Miwa, J. Life span of the wild and mutant nematode *Caenorhabditis elegans*. Effects of sex, sterilization, and temperature. *Exp. Gerontol.* **17**, 163–172 (1982). 10.1016/0531-5565(82)90052-3 6213421

[6] Lamb, M. J. Temperature and lifespan in *Drosophila*. *Nature* **220**, 808–809 (1968). 10.1038/220808a0 5698761

[7] Liu, R. K. & Walford, R. L. Increased growth and life-span with lowered ambient temperature in annual fish *Cynolebias adloffi*. *Nature* **212**, 1277–1278 10.1038/2121277a0 (1966).

[8] Valenzano, D. R., Terzibasi, E., Cattaneo, A., Domenici, L. & Cellerino, A. Temperature affects longevity and age-related locomotor and cognitive decay in the short-lived fish *Nothobranchius furzeri*. *Aging Cell* **5**, 275–278 10.1111/j.1474-9726.2006.00212.x (2006). 16842500

[9] Lee, D. et al. MDT-15/MED15 permits longevity at low temperature via enhancing lipidostasis and proteostasis. *PLoS Biol.* **17**, e3000415 10.1371/journal.pbio.3000415 (2019). PMC6692015 31408455

[10] Lee, H. J. et al. Prostaglandin signals from adult germline stem cells delay somatic ageing of *Caenorhabditis elegans*. *Nat. Metab.* **1**, 790–810 10.1038/s42255-019-0097-9 (2019). PMC6726479 31485561

[11] Xiao, R. et al. A genetic program promotes* C. elegans* longevity at cold temperatures via a thermosensitive TRP channel. *Cell* **152**, 806–817 10.1016/j.cell.2013.01.020 (2013). PMC3594097 23415228

[12] Zhang, B. et al. Environmental temperature differentially modulates *C. elegans* longevity through a thermosensitive TRP channel. *Cell Rep.* **11**, 1414–1424 10.1016/j.celrep.2015.04.066 (2015). PMC4758836 26027928

[13] Protsiv, M., Ley, C., Lankester, J., Hastie, T. & Parsonnet, J. Decreasing human body temperature in the United States since the industrial revolution. *eLife* **9**, e49555 10.7554/eLife.49555 (2020). PMC6946399 31908267

[14] Waalen, J. & Buxbaum, J. N. Is older colder or colder older? The association of age with body temperature in 18,630 individuals. *J. Gerontol. A* **66**, 487–492 10.1093/gerona/glr001 (2011). PMC3107024 21324956

[15] Loeb, J. & Northrop, J. H. Is there a temperature coefficient for the duration of life? *Proc. Natl Acad. Sci. USA* **2**, 456–457 (1916). 10.1073/pnas.2.8.456 PMC1091065 16586628

[16] Fanet, H., Capuron, L., Castanon, N., Calon, F. & Vancassel, S. Tetrahydrobioterin (BH4) pathway: from metabolism to neuropsychiatry. *Curr. Neuropharmacol.* **19**, 591–609 10.2174/1570159X18666200729103529 (2021). PMC8573752 32744952

[17] Werner, E. R., Blau, N. & Thony, B. Tetrahydrobiopterin: biochemistry and pathophysiology. *Biochem. J.* **438**, 397–414 10.1042/BJ20110293 (2011). 21867484

[18] Loer, C. M. et al. Cuticle integrity and biogenic amine synthesis in *Caenorhabditis elegans* require the cofactor tetrahydrobiopterin (BH4). *Genetics* **200**, 237–253 10.1534/genetics.114.174110 (2015). PMC4423366 25808955

[19] Eichwald, T. et al. Tetrahydrobiopterin: beyond its traditional role as a cofactor. *Antioxidants* **12**, 1037 10.3390/antiox12051037 (2023). PMC10215290 37237903

[20] Ohashi, A. et al. Membrane transport of sepiapterin and dihydrobiopterin by equilibrative nucleoside transporters: a plausible gateway for the salvage pathway of tetrahydrobiopterin biosynthesis. *Mol. Genet. Metab.* **102**, 18–28 10.1016/j.ymgme.2010.09.005 (2011). 20956085

[21] Egge, N. et al. Age-onset phosphorylation of a minor actin variant promotes intestinal barrier dysfunction. *Dev. Cell* **51**, 587–601 10.1016/j.devcel.2019.11.001 (2019). PMC6897307 31794717

[22] Koyuncu, S. et al. Rewiring of the ubiquitinated proteome determines ageing in *C. elegans*. *Nature* **596**, 285–290 10.1038/s41586-021-03781-z (2021). PMC8357631 34321666

[23] Dillin, A. et al. Rates of behavior and aging specified by mitochondrial function during development. *Science* **298**, 2398–2401 10.1126/science.1077780 (2002). 12471266

[24] Durieux, J., Wolff, S. & Dillin, A. The cell-non-autonomous nature of electron transport chain-mediated longevity. *Cell* **144**, 79–91 10.1016/j.cell.2010.12.016 (2011). PMC3062502 21215371

[25] Moran, G. R. 4-Hydroxyphenylpyruvate dioxygenase. *Arch. Biochem. Biophys.* **433**, 117–128 10.1016/j.abb.2004.08.015 (2005). 15581571

[26] Hommen, F., Bilican, S. & Vilchez, D. Protein clearance strategies for disease intervention. *J. Neural Transm.* 10.1007/s00702-021-02431-y (2021). PMC8541819 34689261

[27] Brignull, H. R., Moore, F. E., Tang, S. J. & Morimoto, R. I. Polyglutamine proteins at the pathogenic threshold display neuron-specific aggregation in a pan-neuronal *Caenorhabditis elegans* model. *J. Neurosci.* **26**, 7597–7606 10.1523/JNEUROSCI.0990-06.2006 (2006). PMC6674286 16855087

[28] Koyuncu, S. et al. The aging factor EPS8 induces disease-related protein aggregation through RAC signaling hyperactivation. *Nat. Aging*10.1038/s43587-025-00943-w (2025). PMC12443605 40903652

[29] Kitamura, A. et al. Cytosolic chaperonin prevents polyglutamine toxicity with altering the aggregation state. *Nat. Cell Biol.* **8**, 1163–1170 10.1038/ncb1478 (2006). 16980958

[30] Noormohammadi, A. et al. Somatic increase of CCT8 mimics proteostasis of human pluripotent stem cells and extends *C. elegans* lifespan. *Nat. Commun.* **7**, 13649 10.1038/ncomms13649 (2016). PMC5133698 27892468

[31] Tam, S., Geller, R., Spiess, C. & Frydman, J. The chaperonin TRiC controls polyglutamine aggregation and toxicity through subunit-specific interactions. *Nat. Cell Biol.* **8**, 1155–1162 10.1038/ncb1477 (2006). PMC2829982 16980959

[32] Morimoto, R. I. The heat shock response: systems biology of proteotoxic stress in aging and disease. *Cold Spring Harb. Symp. Quant. Biol.* **76**, 91–99 10.1101/sqb.2012.76.010637 (2011). 22371371

[33] Balaji, V. et al. A dimer-monomer switch controls CHIP-dependent substrate ubiquitylation and processing. *Mol. Cell* **82**, 3239–3254 10.1016/j.molcel.2022.08.003 (2022). 36027913

[34] Llamas, E. et al. In planta expression of human polyQ-expanded huntingtin fragment reveals mechanisms to prevent disease-related protein aggregation. *Nat. Aging*10.1038/s43587-023-00502-1 (2023). PMC10645592 37783816

[35] Alirzayeva, H. et al. ALS-FUS mutations cause abnormal PARylation and histone H1.2 interaction, leading to pathological changes. *Cell Rep.* **43**, 114626 10.1016/j.celrep.2024.114626 (2024). 39167487

[36] Crabtree, M. J., Hale, A. B. & Channon, K. M. Dihydrofolate reductase protects endothelial nitric oxide synthase from uncoupling in tetrahydrobiopterin deficiency. *Free Radic. Biol. Med.* **50**, 1639–1646 10.1016/j.freeradbiomed.2011.03.010 (2011). PMC3121954 21402147

[37] Crabtree, M. J., Smith, C. L., Lam, G., Goligorsky, M. S. & Gross, S. S. Ratio of 5,6,7,8-tetrahydrobiopterin to 7,8-dihydrobiopterin in endothelial cells determines glucose-elicited changes in NO vs. superoxide production by eNOS. *Am. J. Physiol. Heart Circ. Physiol.* **294**, H1530–H1540 10.1152/ajpheart.00823.2007 (2008). PMC2722919 18192221

[38] Janaszak-Jasiecka, A., Ploska, A., Wieronska, J. M., Dobrucki, L. W. & Kalinowski, L. Endothelial dysfunction due to eNOS uncoupling: molecular mechanisms as potential therapeutic targets. *Cell. Mol. Biol. Lett.* **28**, 21 10.1186/s11658-023-00423-2 (2023). PMC9996905 36890458

[39] Brenner, S. The genetics of *Caenorhabditis elegans*. *Genetics* **77**, 71–94 (1974). 10.1093/genetics/77.1.71 PMC1213120 4366476

[40] Husken, K. et al. Maintenance of the intestinal tube in *Caenorhabditis elegans*: the role of the intermediate filament protein IFC-2. *Differentiation* **76**, 881–896 10.1111/j.1432-0436.2008.00264.x (2008). 18452552

[41] Kumsta, C. et al. The autophagy receptor p62/SQST-1 promotes proteostasis and longevity in *C. elegans* by inducing autophagy. *Nat. Commun.* **10**, 5648 10.1038/s41467-019-13540-4 (2019). PMC6906454 31827090

[42] Yigit, E. et al. Analysis of the *C. elegans* Argonaute family reveals that distinct Argonautes act sequentially during RNAi. *Cell* **127**, 747–757 10.1016/j.cell.2006.09.033 (2006). 17110334

[43] Watts, J. S. et al. New strains for tissue-specific RNAi studies in *Caenorhabditis elegans*. *G3* **10**, 4167–4176 10.1534/g3.120.401749 (2020). PMC7642939 32943454

[44] Qadota, H. et al. Establishment of a tissue-specific RNAi system in *C. elegans*. *Gene* **400**, 166–173 10.1016/j.gene.2007.06.020 (2007). PMC3086655 17681718

[45] Yang, Y. et al. Autophagy protein ATG-16.2 and its WD40 domain mediate the beneficial effects of inhibiting early-acting autophagy genes in *C. elegans* neurons. *Nat. Aging* **4**, 198–212 10.1038/s43587-023-00548-1 (2024). PMC11022750 38177330

[46] Espelt, M. V., Estevez, A. Y., Yin, X. & Strange, K. Oscillatory Ca^2+^ signaling in the isolated *Caenorhabditis elegans* intestine: role of the inositol-1,4,5-trisphosphate receptor and phospholipases C β and γ. *J. Gen. Physiol.* **126**, 379–392 10.1085/jgp.200509355 (2005). PMC2266627 16186564

[47] Noormohammadi, A. et al. Mechanisms of protein homeostasis (proteostasis) maintain stem cell identity in mammalian pluripotent stem cells. *Cell. Mol. Life Sci.* **75**, 275–290 10.1007/s00018-017-2602-1 (2018). PMC11105389 28748323

[48] Ghanta, K. S. & Mello, C. C. Melting dsDNA donor molecules greatly improves precision genome editing in *Caenorhabditis elegans*. *Genetics* **216**, 643–650 10.1534/genetics.120.303564 (2020). PMC7648581 32963112

[49] Amrit, F. R., Ratnappan, R., Keith, S. A. & Ghazi, A. The *C. elegans* lifespan assay toolkit. *Methods* **68**, 465–475 10.1016/j.ymeth.2014.04.002 (2014). 24727064

[50] Fernandez-Abascal, J. et al. A glial ClC Cl^−^ channel mediates nose touch responses in *C. elegans*. *Neuron* **110**, 470–485 10.1016/j.neuron.2021.11.010 (2022). PMC8813913 34861150

[51] Koopman, M., Gungordu, L., Seinstra, R. I. & Nollen, E. A. A. Neuronal overexpression of hTDP-43 in *Caenorhabditis elegans* impairs different neuronally controlled behaviors and decreases fecundity. *Micropubl. Biol.* 10.17912/micropub.biology.000769 (2023). PMC10157382 37151215

[52] Segref, A. et al. Thermosensation in *Caenorhabditis elegans* is linked to ubiquitin-dependent protein turnover via insulin and calcineurin signalling. *Nat. Commun.* **13**, 5874 10.1038/s41467-022-33467-7 (2022). PMC9534930 36198694

[53] Porta-de-la-Riva, M., Fontrodona, L., Villanueva, A. & Ceron, J. Basic *Caenorhabditis elegans* methods: synchronization and observation. *J. Vis. Exp.* **64**, e4019 10.3791/4019 (2012). PMC3607348 22710399

[54] Dieterle, F., Ross, A., Schlotterbeck, G. & Senn, H. Probabilistic quotient normalization as robust method to account for dilution of complex biological mixtures. Application in H-1 NMR metabonomics. *Anal. Chem.* **78**, 4281–4290 10.1021/ac051632c (2006). 16808434

[55] Schmidt, C. et al. Integrated metabolomics data analysis to generate mechanistic hypotheses with MetaProViz. *Mol. Syst. Biol.* 10.1038/s44320-026-00231-8 (2026). PMC13639010 42608578

[56] Pollard, K. S., Dudoit, S. & van der Laan, M. J. Multiple testing procedures: the multtest package and applications to genomics. In *Bioinformatics and Computational Biology Solutions Using R and Bioconductor. Statistics for Biology and Health* (eds Gentleman, R. et al.) 249–271 (Springer, 2005).

[57] Searle, B. C. et al. Generating high quality libraries for DIA MS with empirically corrected peptide predictions. *Nat. Commun.* **11**, 1548 10.1038/s41467-020-15346-1 (2020). PMC7096433 32214105

[58] Chambers, M. C. et al. A cross-platform toolkit for mass spectrometry and proteomics. *Nat. Biotechnol.* **30**, 918–920 10.1038/nbt.2377 (2012). PMC3471674 23051804

[59] Demichev, V., Messner, C. B., Vernardis, S. I., Lilley, K. S. & Ralser, M. DIA-NN: neural networks and interference correction enable deep proteome coverage in high throughput. *Nat. Methods* **17**, 41–44 10.1038/s41592-019-0638-x (2020). PMC6949130 31768060

[60] Tyanova, S. et al. The Perseus computational platform for comprehensive analysis of (prote)omics data. *Nat. Methods* **13**, 731–740 10.1038/nmeth.3901 (2016). 27348712

[61] Hoogewijs, D., Houthoofd, K., Matthijssens, F., Vandesompele, J. & Vanfleteren, J. R. Selection and validation of a set of reliable reference genes for quantitative sod gene expression analysis in *C. elegans*. *BMC Mol. Biol.* **9**, 9 10.1186/1471-2199-9-9 (2008). PMC2254638 18211699

[62] Livak, K. J. & Schmittgen, T. D. Analysis of relative gene expression data using real-time quantitative PCR and the method. *Methods* **25**, 402–408 10.1006/meth.2001.1262 (2001). 11846609

[63] Dillin, A., Crawford, D. K. & Kenyon, C. Timing requirements for insulin/IGF-1 signaling in *C. elegans*. *Science* **298**, 830–834 10.1126/science.1074240 (2002). 12399591

[64] Artan, M., Schoen, H. & de Bono, M. Proximity labeling of DAF-16 FOXO highlights aging regulatory proteins. *Nat. Commun.* **16**, 11355 10.1038/s41467-025-66409-0 (2025). PMC12727705 41381452

[65] Han, S. K. et al. OASIS 2: online application for survival analysis 2 with features for the analysis of maximal lifespan and healthspan in aging research. *Oncotarget* **7**, 56147–56152 10.18632/oncotarget.11269 (2016). PMC5302902 27528229

[66] Calculli, G. et al. Systemic regulation of mitochondria by germline proteostasis prevents protein aggregation in the soma of *C. elegans*. *Sci. Adv.* **7**, 26 10.1126/sciadv.abg3012 (2021). PMC8232903 34172445

[67] Ward, S. Chemotaxis by the nematode *Caenorhabditis elegans*: identification of attractants and analysis of the response by use of mutants. *Proc. Natl Acad. Sci. USA* **70**, 817–821 10.1073/pnas.70.3.817 (1973). PMC433366 4351805

[68] Chalfie, M., Hart, A. C., Rankin, C. H. & Goodman, M. B. Assaying mechanosensation. *Wormbook*10.1895/wormbook.1.172.1 (2014). PMC4448936 25093996

[69] Hart, A. C., Kass, J., Shapiro, J. E. & Kaplan, J. M. Distinct signaling pathways mediate touch and osmosensory responses in a polymodal sensory neuron. *J. Neurosci.* **19**, 1952–1958 10.1523/JNEUROSCI.19-06-01952.1999 (1999). PMC6782580 10066248

[70] Lenzi, J. et al. ALS mutant FUS proteins are recruited into stress granules in induced pluripotent stem cell-derived motoneurons. *Dis. Model. Mech.* **8**, 755–766 10.1242/dmm.020099 (2015). PMC4486861 26035390

[71] Koyuncu, S. et al. The ubiquitin ligase UBR5 suppresses proteostasis collapse in pluripotent stem cells from Huntington’s disease patients. *Nat. Commun.* **9**, 2886 10.1038/s41467-018-05320-3 (2018). PMC6056416 30038412

[72] Hill, S. J. et al. Two familial ALS proteins function in prevention/repair of transcription-associated DNA damage. *Proc. Natl Acad. Sci. USA* **113**, E7701–E7709 10.1073/pnas.1611673113 (2016). PMC5137757 27849576

[73] Dormann, D. et al. ALS-associated fused in sarcoma (FUS) mutations disrupt transportin-mediated nuclear import. *EMBO J.* **29**, 2841–2857 10.1038/emboj.2010.143 (2010). PMC2924641 20606625

[74] Roczniak-Ferguson, A. & Ferguson, S. M. Pleiotropic requirements for human TDP-43 in the regulation of cell and organelle homeostasis. *Life Sci. Alliance* **2**, e201900358 10.26508/lsa.201900358 (2019). PMC6749094 31527135

[75] Sud, M. et al. Metabolomics Workbench: an international repository for metabolomics data and metadata, metabolite standards, protocols, tutorials and training, and analysis tools. *Nucleic Acids Res.* **44**, D463–D470 10.1093/nar/gkv1042 (2016). PMC4702780 26467476

