# Supplementary material for: Cold activates a BH2 metabolic network promoting longevity and proteostasis
Source: Nature. 2026 Oct 21;659(8140):480–9. doi: 10.1038/s41586-026-11152-9 (PMC13754623; doi:10.1038/s41586-026-11152-9)
Supplement: Supplementary file 1 [file 41586_2026_11152_MOESM11_ESM.pdf]

nature portfolio

Peer Review File

Cold activates a BH2 metabolic network promoting longevity and proteostasis

Corresponding Author: Professor David Vilchez

Any redactions in this file are there to maintain patient confidentiality, the confidentiality of unpublished data, or to remove third-party material.

This file contains all reviewer reports in order by version, followed by all author rebuttals in order by version.

Version 0:

Reviewer comments:

Referee #1

(Remarks to the Author)
In this manuscript entitled "Cold temperature activates a BH2-driven intertissue metabolic network that promotes longevity and proteostasis," The authors investigate how moderate cold exposure extends lifespan and preserves proteostasis in Caenorhabditis elegans. Using metabolomics, genetics, and tissue-specific manipulations, the authors identify dihydrobiopterin (BH2) as a cold-induced metabolite synthesized in muscle via CAT-4/GCH1. BH2 is transported to distal tissues and converted to BH4, activating PAH-1–dependent tyrosine synthesis to promote longevity. Muscle-specific overexpression of cat-4 extends lifespan and suppresses protein aggregation in neurons and intestine, even at standard temperatures. Importantly, BH2 or tyrosine supplementation reduces aggregation of disease-associated proteins in human cells. Overall, the study proposes a novel metabolite-driven, cell non-autonomous pathway linking temperature to systemic aging regulation. Given that cold-induced longevity has been studied extensively, the authors should better clarify what fundamentally differentiates their findings from prior work and how this study advances the field at a broader conceptual level. Following are my concerns that the authors should address to improve this paper.
Major comments
1. The authors primarily describe BH2 as a stable reservoir and transport intermediate for BH4, but it is crucial to explicitly address in the Introduction and Discussion that in mammalian systems, elevated BH2 levels are often associated with pathological conditions (i.e. eNOS uncoupling). Since BH2 acts as a competitive inhibitor of BH4-dependent enzymes due to its structural similarity, the authors should investigate whether excessive BH2 supplementation leads to toxicity or a decline in lifespan in C. elegans. A central concern is whether the observed longevity is a genuine metabolic benefit or a hormetic response to low level metabolic stress, similar to the case of mitochondrial uncouplers like DNP, which extend lifespan in C. elegans but remain clinically limited due to severe systemic toxicity. Furthermore, regarding the mammalian cell studies in Figure 7, the authors must experimentally demonstrate that the observed proteostasis benefits actually outweigh or overcome these known toxicities. Without such evidence, the translational significance of the findings is substantially weakened, as the pathological consequences typically associated with BH2 accumulation in higher organisms cannot be ignored.
2. The authors rely heavily on adult-specific RNAi to support their claims regarding the cold-induced longevity pathway, but the lack of validation using genetic mutants remains a significant concern. While RNAi provides temporal control, it often results in incomplete knockdown or off-target effects, and the residual enzymatic activity of CAT-4 or DHFR-1 could be sufficient to maintain metabolic flux, potentially masking the full physiological impact. For instance, the authors show that knockdown of recycling pathway enzymes, such as pcbd-1 or qdpr-1, does not affect lifespan at 15°C. However, this negative result cannot be considered conclusive without confirming the phenotype in null mutants to rule out insufficient RNAi efficiency. Consequently, the claim that the salvage pathway, but not the recycling pathway, accounts for the observed lifespan-increasing effects is not sufficiently supported. Additionally, the observation that cat-4(e3015) mutants are short-lived at both 15°C and 20°C contrasts with the cold-specific effect seen in adult RNAi. To rigorously define the necessity of these biopterin metabolic components, the authors should validate their key findings using available mutant strains and consider utilizing conditional protein degradation systems, such as the auxin-inducible degron (AID), to definitively separate developmental requirements from adult-specific longevity functions.
3. It is unclear throughout the narrative of the manuscript why cat-4 expression increases at lower temperatures. Are there any specific transcription factor that contributes to the expression of cat-4? This point at least should be discussed to explain

the mechanism of increased cat-4 expression at moderately lower temperatures. It would also be helpful to clarify whether this induction is predominantly observed in muscles or occurs more broadly across tissues in response to cold temperature.
4. In Figure 3, the authors showed that RNAi knockdown of ent, which encodes BH2 transporter, decreased the lifespan of animals at 15°C. To strengthen the statement that ENT contributes to BH2 transport at cold temperature, the authors should measure the lifespan of ent mutant animals with muscle-specific knockdown of cat-4 and neuron-specific knockdown of dhfr-1. Alternatively, examining whether knockdown of ent masks lifespan-increasing effect of cat-4 overexpression will reinforce the cell non-autonomous role of BH2.
5. Although the authors demonstrate that tyrosine is required and sufficient for longevity, the downstream molecular pathways remain unclear. The authors should consider examining whether cold-induced transcriptomic datasets (e.g., GEO: GSE62297) reveal enrichment of tyrosine metabolism–related or established longevity-associated gene signatures. This would clarify whether tyrosine acts through a defined regulatory program rather than general metabolic support and would strengthen the mechanistic depth of the study.
6. In the human cell experiments (Fig. 7), aggregation is reduced without changes in total protein levels. The authors should clarify whether proteasome or autophagy pathways are altered by BH2 or tyrosine treatment to exclude indirect proteostasis effects.

Minor comments

1. In the Abstract and Introduction, BH2 is described as inactive yet central; the authors should more precisely distinguish between BH2 as a transport reservoir and BH4 as the enzymatically active cofactor.
2. The metabolomics criteria in Fig. 1 are stringent. However, the authors should provide a clearer rationale for prioritizing BH2 over the other 63 cold-induced metabolites and include a more detailed comparison of effect sizes and reproducibility across independent datasets.
3. The authors showed that aging and cold temperature altered the levels of BH4 using ELISA. Measuring BH4 or BH2 levels in cat-4-overexpressing animals at various temperatures would strengthen the data using cat-4 overexpression.
4. In Fig. 1, the cutoff criteria are described in the Results; these thresholds should also be clearly stated in the figure legend for transparency.
5. In Fig. 1a, the labeling/annotation for BH2 is difficult to interpret because it is not visually clear which specific feature or dot corresponds to BH2; adding a clearer label, arrow placement, or an inset/legend mapping would substantially improve figure readability for non-specialist readers.
6. The narrative of this paper underscores the enzymes CAT-4, DHFR-1, and PCBD-1, visualized in the pathway in figure 1c. Although the functions of these enzymes are introduced as rate-limiting steps in the main text, such emphasis is not present in the figure. I therefore suggest that the authors stress the central enzymes in the figure panel.
7. Figure citation strings appear to be repeated verbatim in close proximity in several places (e.g., "Fig. 1a,b and Supplementary Fig. 1a–c" repeated twice), and the authors may consider reducing redundancy by citing the full set once and then using a shorter reference thereafter, or by retaining supplementary citations only where the supplementary panels add non-overlapping evidence that is explicitly discussed in that sentence.
8. In figure 3f and g, scale bars are missing.
9. Regarding Figure 4, I recommend that the authors explain why they focused on the aggregation of IFB-2 in detail to improve readers' understanding.
10. In Supplementary Figure 4b, the authors seem to have compared wild-type strain and him-8(e1489); cat-4(e3015). Are there any rationales using double mutants rather than cat-4(e3015)? Furthermore, mutant animals used in lifespan assays in Supplementary Figure 4b and Supplementary Figure 8 does not seem to be backcrossed to WT. The authors should clarify these points.

Referee #2

(Remarks to the Author)

The manuscript by Lee et al explores how moderate cold temperatures activate a metabolite-driven intertissue signaling network that promotes longevity and proteostasis, focusing on the role of dihydrobiopterin (BH2) and its bioactive form tetrahydrobiopterin (BH4).

Moderate cold temperatures (15°C) extend lifespan in Caenorhabditis elegans by upregulating the enzyme GCH1/CAT-4 in muscle cells, leading to increased BH2 production. BH2 is transported to distal tissues (intestine, neurons, germline), where it is converted into BH4, a cofactor for enzymes like phenylalanine hydroxylase (PAH-1), which enhances tyrosine synthesis and contributes to longevity.

Interestingly, BH2 synthesis occurs in muscle cells, but its benefits are distributed systemically. BH2 is transported to other tissues via nucleoside transporters (e.g., ENT1-ENT7 paralogs) and converted into BH4 by DHFR-1 in neurons, intestine, and germline. Muscle-specific overexpression of CAT-4 extends lifespan and suppresses protein aggregation at both cold and standard temperatures, demonstrating the central role of muscle in generating systemic pro-longevity signals.

Elevated BH2 levels prevent age-related protein aggregation and neuronal dysfunction. Muscle-derived BH2 suppresses pathological protein aggregation in distal tissues, including neurons and the intestine, preserving tissue integrity and function during aging. The authors go on to show that BH2 supplementation in human cultured cells can also protect from PolyQ repeat proteins as well as disease variants toward ALS.

When I first read the title and abstract of this manuscript, several key questions came to mind. First, does this work in tpa1 (-) animals? Can they identify the transporters to formally show that BH2 mad. Can they identify the biosynthetic enzymes to make BH4 in distal tissues? Is production of BH2 sufficient for all of the lifespan and proteostasis effects? What does BH4 affect downstream to elicit these positive effects? Finally, what is the tradeoff?

In short order, almost every single one of these questions has been addressed and highlighted below are some remaining suggestions:

1) While the authors show that BH4 works on PAH-1 to elicit the effects in distal tissues, this does not fully explain the

increased proteostasis capacity. Do the authors have any data or suggestions on how PAH-1 can affect proteostasis? Is this HSF1 dependent? What is the program that is increased by BH4 that is dependent upon PAH-1?
2) The experiment to show that PAH-1 is downstream of BH4 is compelling, but the negative data of the other 3 BH4 binding proteins needs to be done with mutants and not RNAi to ensure that their involvement is not being missed.
3) The authors show that the cold induced lifespan that requires BH2 is not daf-2 dependent, but it seems that other longevity pathways that are directly related to metabolism could be overlapping, such as the ETC derived pathway or even calorie restriction. Have these been tried?
4) Might have missed this, but does overexpression of cat-2 extend lifespan in TRPA(-) animal?
In summary, the research identifies BH2/BH4 signaling as a central mechanism through which moderate cold temperatures promote longevity and systemic proteostasis, with potential applications for combating age-related diseases in humans. The findings as the stand are well done and easily interpretable. The impact of this work could have potential, unknown, positive benefits on diseases of proteostasis,

Version 1:

Reviewer comments:

Referee #1

(Remarks to the Author)
The authors addressed my previous concerns successfully.

Referee #2

(Remarks to the Author)
Thank you for addressing all of my concerns.

Open Access This Peer Review File is licensed under a Creative Commons Attribution 4.0 International License, which permits use, sharing, adaptation, distribution and reproduction in any medium or format, as long as you give appropriate credit to the original author(s) and the source, provide a link to the Creative Commons license, and indicate if changes were made.
In cases where reviewers are anonymous, credit should be given to 'Anonymous Referee' and the source.
The images or other third party material in this Peer Review File are included in the article’s Creative Commons license, unless indicated otherwise in a credit line to the material. If material is not included in the article’s Creative Commons license and your intended use is not permitted by statutory regulation or exceeds the permitted use, you will need to obtain permission directly from the copyright holder.
To view a copy of this license, visit https://creativecommons.org/licenses/by/4.0/

Referees' comments:

Referee #1 (Remarks to the Author):

In this manuscript entitled "Cold temperature activates a BH2-driven intertissue metabolic network that promotes longevity and proteostasis," The authors investigate how moderate cold exposure extends lifespan and preserves proteostasis in Caenorhabditis elegans. Using metabolomics, genetics, and tissue-specific manipulations, the authors identify dihydrobiopterin (BH2) as a cold-induced metabolite synthesized in muscle via CAT-4/GCH1. BH2 is transported to distal tissues and converted to BH4, activating PAH-1–dependent tyrosine synthesis to promote longevity. Muscle-specific overexpression of cat-4 extends lifespan and suppresses protein aggregation in neurons and intestine, even at standard temperatures. Importantly, BH2 or tyrosine supplementation reduces aggregation of disease-associated proteins in human cells. Overall, the study proposes a novel metabolite-driven, cell non-autonomous pathway linking temperature to systemic aging regulation. Given that cold-induced longevity has been studied extensively, the authors should better clarify what fundamentally differentiates their findings from prior work and how this study advances the field at a broader conceptual level. Following are my concerns that the authors should address to improve this paper.

We thank Reviewer #1 for the careful and thoughtful evaluation of our work and for the constructive comments and suggestions, which have significantly improved the manuscript. We have addressed all of the Reviewer's points through additional analyses, new experiments, and substantial revisions to the text. In addition, we have better clarified the novelty and conceptual advance of our findings relative to previous studies of cold-induced longevity. We believe that these revisions have strengthened both the mechanistic depth and overall clarity of the manuscript.

Major comments

1. The authors primarily describe BH2 as a stable reservoir and transport intermediate for BH4, but it is crucial to explicitly address in the Introduction and Discussion that in mammalian systems, elevated BH2 levels are often associated with pathological conditions (i.e. eNOS uncoupling). Since BH2 acts as a competitive inhibitor of BH4-dependent enzymes due to its structural similarity, the authors should investigate whether excessive BH2 supplementation leads to toxicity or a decline in lifespan in C. elegans. A central concern is whether the observed longevity is a genuine metabolic benefit or a hormetic response to low level metabolic stress, similar to the case of mitochondrial uncouplers like DNP, which extend lifespan in C. elegans but remain clinically limited due to severe systemic toxicity. Furthermore, regarding the mammalian cell studies in Figure 7, the authors must experimentally demonstrate that the observed proteostasis benefits actually outweigh or overcome these known toxicities. Without such evidence, the translational significance of the findings is

substantially weakened, as the pathological consequences typically associated with BH2 accumulation in higher organisms cannot be ignored.

We thank the Reviewer for raising these important points. We agree that the relationship between BH2 and endothelial nitric oxide synthase (eNOS) uncoupling should be explicitly discussed, and we have now addressed this point in the main text and placed our findings in the context of the vascular literature. Importantly, studies of eNOS uncoupling do not conclude that BH2 supplementation is toxic (e.g., PMIDs: 18192221, 21402147 and reviewed in PMID: 36890458). Rather, they demonstrate that eNOS uncoupling in endothelial cells arises under conditions of suboptimal BH4 availability and a consequent reduction in the BH4/BH2 ratio, typically driven by oxidative stress, which promotes oxidation of BH4 to BH2 (PMID: 18192221), and/or by impaired synthesis of BH4 from BH2 via the DHFR-dependent salvage pathway (PMID: 21402147). Under these conditions, BH2 can compete with BH4 for binding to eNOS, resulting in eNOS uncoupling and a shift in its enzymatic activity from nitric oxide production toward superoxide generation. Thus, the pathological phenotype is primarily associated with disrupted BH4 homeostasis rather than elevated BH2 per se or BH2 supplementation.

Consistent with this framework, previous work from the same group has shown that in endothelial cells with intact DHFR-dependent salvage activity, BH2 supplementation efficiently increases intracellular BH4 levels and alleviates eNOS uncoupling under oxidative stress, even more effectively than direct BH4 supplementation (PMID: 18192221). Likewise, mouse studies demonstrate that detrimental effects associated with BH2 accumulation become prominent primarily when DHFR activity is inhibited (PMID: 21402147), highlighting that the capacity to generate BH4 from BH2 through the salvage pathway is a critical determinant of the physiological outcome.

Our findings are fully consistent with this model. Throughout our study, the protective effects of BH2 require an intact DHFR-dependent salvage pathway. In *C. elegans*, knockdown of *dhfr-1* reduces cold-induced longevity, demonstrating that conversion of BH2 into bioactive BH4 is essential for the observed beneficial effects. Accordingly, we propose that BH2 functions as a stable transport intermediate and reservoir whose beneficial effects depend on regulated conversion into BH4. We have revised the main text to explicitly distinguish well-characterized endothelial eNOS uncoupling under conditions of oxidative stress and impaired DHFR-dependent activity from the beneficial effects of the BH2/BH4 pathway described here.

In addition, we have now assessed whether BH2 supplementation induces superoxide production in our human cell models. In contrast to paraquat-induced oxidative stress, neither standard exogenous BH2 supplementation (10 μM BH2) nor a 20-fold higher concentration increased the percentage of cells with detectable superoxide levels (**Extended Data Fig. 12f**). Moreover, even high doses of BH2 reduced pathological protein aggregation (**Extended Data Fig. 12g**), further

supporting that BH2 supplementation can confer proteostasis benefits without inducing detectable superoxide production indicative of disrupted BH4 balance.

This Reviewer's comment prompted us to consider a potential limitation of BH2 treatment that we felt was important to address experimentally. Given that oxidative stress promotes BH4 oxidation to BH2, we hypothesized that such conditions might limit the efficacy of BH2 supplementation. Indeed, paraquat-induced oxidative stress abolished the protective effect of BH2 on protein aggregation (**Extended Data Fig. 12h**). These findings are consistent with a requirement for efficient BH2-to-BH4 conversion to mediate proteostasis benefits, which becomes compromised when oxidative stress induces excessive oxidation of BH4. In contrast, tyrosine supplementation still reduced pathological protein aggregation under oxidative stress (**Extended Data Fig. 12i**), suggesting that this downstream effector of BH2/BH4 metabolism can bypass conditions in which BH2 cannot be efficiently converted into BH4.

Accordingly, we have now revised the main text to explicitly reflect these considerations. For instance, the Results section now says: "*In contrast to C. elegans, mammalian cells express an additional BH4-dependent enzyme, nitric oxide synthase (NOS)[23,25]. Under conditions that lower intracellular BH4 availability, such as oxidative stress—which promotes excessive oxidation of BH4 to BH2—or impaired DHFR activity, BH2 can compete with BH4 for binding to endothelial NOS (eNOS) in the vascular system[47-50]. This shifts eNOS activity from nitric oxide production toward superoxide generation, a process known as eNOS uncoupling[50]. Importantly, eNOS uncoupling is primarily linked to suboptimal BH4 availability and a consequent reduction in the BH4/BH2 ratio, rather than to elevated BH2 levels per se or BH2 supplementation. In fact, when cells retain an intact DHFR-dependent salvage pathway, BH2 supplementation increases intracellular BH4 levels and alleviates eNOS uncoupling under oxidative stress[47,48]. In contrast to paraquat-induced oxidative stress, neither 10 µM BH2 nor a 20-fold higher concentration increased the percentage of cells with detectable superoxide levels (**Extended Data Fig. 12f**). Moreover, even high doses of BH2 reduced pathological protein aggregation (**Extended Data Fig. 12g**), supporting that BH2 supplementation can confer proteostasis benefits without inducing detectable superoxide production indicative of disrupted BH4 balance*". The Discussion section now says: "*An important consideration for the translational potential of BH2 is its interaction with NOS, a BH4-dependent enzyme present in mammalian but not C. elegans cells. In the vascular system, oxidative stress induces excessive oxidation of BH4 to BH2, lowering the BH4/BH2 ratio below optimal levels. Under these conditions, BH2 can compete with BH4 for binding to eNOS, thereby promoting eNOS uncoupling[48]. Impaired DHFR-dependent conversion of BH2 to BH4 can produce a similar effect[47]. In contrast, when the DHFR-dependent salvage pathway remains functional, BH2 supplementation increases intracellular BH4 and attenuates oxidative stress-induced eNOS uncoupling[48]. These observations are consistent with a model in which the biological consequences of BH2 are determined by its conversion into BH4 through the DHFR-dependent salvage pathway. In our*

cellular models, BH2 supplementation did not increase superoxide production at high concentrations, while still protecting against pathological protein aggregation. Because the BH4/BH2 balance is likely to be highly tissue- and disease-dependent, future studies will be required to define the therapeutic window of BH2 supplementation, particularly with respect to its effects on BH4-dependent NOS activity in vivo under conditions of altered redox state or impaired DHFR activity. Finally, downstream metabolic outputs of the BH2/BH4 pathway, such as tyrosine, may provide an alternative strategy to harness its beneficial effects while bypassing potential limitations associated with BH2 supplementation".

We also thank the Reviewer for raising the important possibility that BH2 supplementation could promote longevity through a hormetic response to low-level metabolic stress rather than through specific metabolic mechanisms. As suggested, we tested whether increasing BH2 concentrations reduces lifespan in *C. elegans*. First, it is important to note that the concentrations used in our study (150–500 µM) are already relatively high compared with those commonly used in mammalian cell culture systems, where BH2 is typically applied in the low micromolar range (e.g., 10 µM; PMID: 18192221). Our dosing strategy is consistent with previous *C. elegans* studies, including prior work using 200 µM biopterin supplementation (PMID: 25808955).

We therefore tested an even higher dose of BH2 (1 mM), representing a two-fold increase over our highest standard concentration (500 µM). Importantly, we did not observe any reduction in lifespan compared with vehicle (DMSO)-treated controls (**Figure R1**), indicating that BH2 does not shorten lifespan even at substantially elevated concentrations. Likewise, we did not observe overt signs of sickness under these conditions. We note that 1 mM represents a relatively high exposure level for this compound in biological systems, and higher concentrations were not pursued due to both solubility constraints and the need to maintain low vehicle DMSO concentrations in lifespan assays.

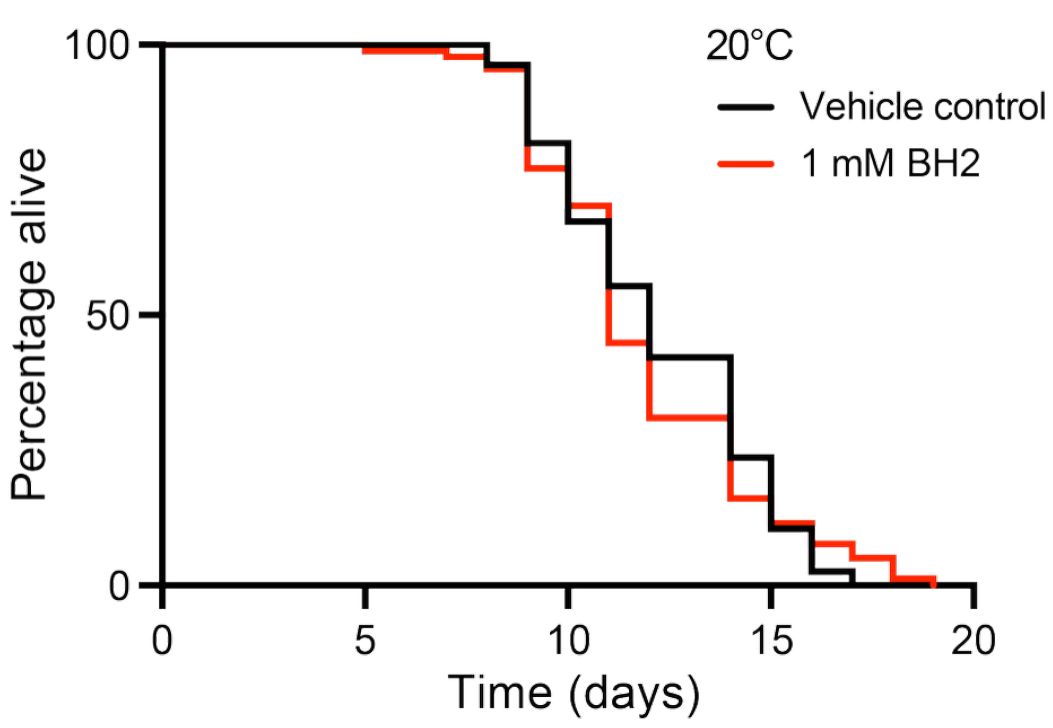

Figure R1. Exogenous supplementation with 1 mM BH2 does not shorten lifespan at 20 °C (Vehicle control (DMSO) mean ± s.e.m.: 12.22 ± 0.29 days, 1 mM BH2: 11.87 ± 0.30, *P* = 0.7830). Comparisons were made by two-sided log-rank test, *n* = 96 worms per condition. Representative of two independent experiments.

Importantly, both 150 µM and 500 µM BH2 supplementation delayed age-related decline in motility, a key indicator of healthspan (**Extended Data Fig. 8c**). Although BH2-treated worms did not display overt signs of sickness, we have now assessed whether its beneficial effects could reflect a hormetic stress response, such

as that induced by mild mitochondrial dysfunction. However, BH2 supplementation did not reduce brood size and did not induce the mitochondrial unfolded protein response (**Extended Data Fig. 8d,e**), two commonly used indicators of stress.

Together, these observations argue against BH2 supplementation causing overt toxicity or inducing a hormetic stress response, and instead support the conclusion that BH2 promotes longevity through a regulated metabolic pathway rather than nonspecific toxicity or stress adaptation. In support of this model, our study provides multiple lines of evidence linking BH2 to a defined metabolic cascade: cold-induced BH2 synthesis is driven by increased CAT-4 expression in muscle, and longevity requires downstream conversion of BH2 to BH4 via DHFR-1, utilization of BH4 by PAH-1, and increased tyrosine production. In addition, tyrosine supplementation rescues the reduction in cold-induced longevity caused by *cat-4* knockdown (**Fig. 4j**), further supporting a functional role for regulated tyrosine metabolism in mediating the beneficial effects of elevated BH2. Collectively, these results support the conclusion that BH2 promotes longevity through a regulated metabolic pathway rather than through nonspecific toxicity or hormetic stress.

2. The authors rely heavily on adult-specific RNAi to support their claims regarding the cold-induced longevity pathway, but the lack of validation using genetic mutants remains a significant concern. While RNAi provides temporal control, it often results in incomplete knockdown or off-target effects, and the residual enzymatic activity of CAT-4 or DHFR-1 could be sufficient to maintain metabolic flux, potentially masking the full physiological impact. For instance, the authors show that knockdown of recycling pathway enzymes, such as pcbd-1 or qdpr-1, does not affect lifespan at 15°C. However, this negative result cannot be considered conclusive without confirming the phenotype in null mutants to rule out insufficient RNAi efficiency. Consequently, the claim that the salvage pathway, but not the recycling pathway, accounts for the observed lifespan-increasing effects is not sufficiently supported. Additionally, the observation that cat-4(e3015) mutants are short-lived at both 15°C and 20°C contrasts with the cold-specific effect seen in adult RNAi. To rigorously define the necessity of these biopterin metabolic components, the authors should validate their key findings using available mutant strains and consider utilizing conditional protein degradation systems, such as the auxin-inducible degron (AID), to definitively separate developmental requirements from adult-specific longevity functions.

We thank the Reviewer for this thoughtful comment. We have now addressed these concerns through clarification of our experimental rationale and additional experiments. Unfortunately, loss-of-function *cat-4* mutants do not provide a suitable model to assess the role of elevated BH2 synthesis during adulthood in cold-induced longevity. BH2 is the major intermediate required for BH4 synthesis in *C. elegans*, and a previous study demonstrated that *cat-4* mutants exhibit near-complete depletion of BH4 (PMID: 25808955), consistent with the key role of CAT-4 in BH2 production. As

expected from the requirement of BH4 as an essential cofactor for multiple enzymes, *cat-4* mutant worms display severe neurotransmitter deficiency, hypersensitivity to stressors, and increased fragility relative to wild-type animals (PMID: 25808955). Consistent with this, we observed that *cat-4* mutant worms appeared severely compromised and were difficult to propagate to obtain synchronized populations for lifespan analyses.

In our initial submission, we focused on *cat-4(e3015)* mutant worms and found that they are short-lived under both 15 °C and 20 °C conditions (now **Extended Data Fig. 3c**). We have now extended our analyses to two additional loss-of-function alleles, *cat-4(ok342)* and *cat-4(tm773)*, which were likewise short-lived at standard temperature (**Extended Data Fig. 3d,e**). Together, these results indicate that constitutive loss of CAT-4 activity causes broad physiological impairment, precluding the use of these mutants to specifically assess cold-induced longevity mechanisms during adulthood.

In contrast, adult-specific RNAi enabled us to perform selective physiological modulation of CAT-4 activity during adulthood (**Fig. 1h**), when cold exposure is sufficient to induce the full longevity phenotype (PMID: 26027928). Importantly, we show that cold exposure only during adulthood increases both CAT-4 expression and BH2 levels. Although *cat-4* RNAi after development might not fully abolish enzyme activity, this treatment was sufficient to reduce BH4 levels to within the physiological range observed in control worms at standard temperature (**Fig. 1h**), allowing us to test whether elevated BH4 synthesis during adulthood specifically contributes to cold-induced longevity without the severe viability defects observed in *cat-4* mutants, which are nearly depleted of BH4. Consistent with this interpretation, adult-specific *cat-4* knockdown selectively shortened lifespan at 15 °C without affecting lifespan at 20 °C or 25 °C (**Fig. 1i**). In contrast, *cat-4* knockdown initiated during early development shortened lifespan at both 15 °C and 20 °C, although the effect remains more pronounced at cold temperature (**Extended Data Fig. 3f**). These findings support the conclusion that CAT-4 activity is essential during development, whereas its upregulation during adulthood specifically contributes to cold-induced longevity.

We acknowledge that RNAi may not achieve complete knockdown and that off-target effects cannot be fully excluded. However, our conclusions are supported by multiple complementary approaches. For instance, muscle-specific *cat-4* overexpression extends lifespan (**Fig. 3b,c**), BH2 supplementation exerts beneficial effects (**Extended Data Fig. 8a,c**), and tyrosine supplementation rescues the shortened lifespan induced by *cat-4* RNAi at cold temperature (**Fig. 4j**). We have now reorganized the figures and revised the text to more clearly explain both the limitations of *cat-4* mutants and the advantages of adult-specific RNAi approaches for assessing the role of CAT-4 in cold-induced longevity.

In addition, as suggested by the Reviewer, we have now generated auxin-inducible degron (AID) strains to enable conditional depletion of CAT-4 and further dissect its adult-specific effect on longevity independently of RNAi efficiency. We first

examined strains in which AID-mediated CAT-4 depletion occurs ubiquitously in somatic tissues through expression of TIR1 under the *eft-3* promoter. We confirmed that auxin treatment efficiently reduces CAT-4 protein levels (please see **Extended Data Fig. 3g**). CAT-4 degradation from early development dramatically reduced lifespan at 20 °C (**Extended Data Fig. 3h**). In contrast, degradation of CAT-4 after development did not affect lifespan at 20 °C, but specifically shortened longevity at 15 °C (**Extended Data Fig. 3i**). These findings are consistent with our *cat-4* RNAi experiments and further support the conclusion that elevated CAT-4 activity during adulthood contributes to cold-induced longevity.

To further investigate the tissue-specific requirement for CAT-4, we generated worms in which AID-mediated CAT-4 depletion was restricted to muscle by expressing TIR1 under the *unc-54* promoter. We confirmed that auxin treatment selectively depleted CAT-4 in muscle, whereas ubiquitous somatic depletion reduced CAT-4 levels in both muscle and intestine (**Extended Data Fig. 6c,d**). Remarkably, muscle-specific depletion of CAT-4 after development was sufficient to fully phenocopy the lifespan-shortening effects of ubiquitous somatic CAT-4 depletion at 15 °C (**Extended Data Fig. 6e**). This effect was specific to cold temperature, as muscle-specific CAT-4 degradation did not affect lifespan at 20 °C (**Extended Data Fig. 6f**). Together, the AID experiments provide further evidence that muscle is the critical tissue in which elevated CAT-4 activity promotes cold-induced longevity.

Similar to CAT-4, DHFR-1 is essential for BH4 synthesis in *C. elegans*, which lacks sepiapterin reductase and therefore depends on the salvage pathway to generate BH4 from BH2. We found that adult-specific *dhfr-1* knockdown shortens lifespan at cold temperature while having no effect at standard temperature (**Fig. 1j**), supporting a role for the salvage pathway in cold-induced longevity. Importantly, neuronal-specific knockdown of *dhfr-1* suppressed the lifespan extension induced by muscle-specific *cat-4* overexpression without affecting control animals at standard temperature (**Fig. 3g and Extended Data Fig. 8m**), further supporting a functional link between elevated BH2 synthesis and its conversion into BH4 in promoting longevity.

To further validate the role of inter-tissue BH2 transport in longevity, we have now performed additional experiments using *ent-1* mutant worms combined with tissue-specific knockdown of *cat-4* or *dhfr-1*. We found that *ent-1* mutant worms are short-lived at cold temperature, and neither muscle-specific knockdown of *cat-4* nor neuronal-specific knockdown of *dhfr-1* further reduced their lifespan (**Extended Data Fig. 6g,h**). Moreover, neuronal-specific knockdown of *ent-1* attenuated the lifespan extension conferred by muscle-specific *cat-4* overexpression at standard temperature (**Fig. 3g**), further supporting a role for inter-tissue BH2 transport in mediating the longevity effects of elevated muscle CAT-4 activity.

We completely agree with the Reviewer that negative results from RNAi experiments (e.g., recycling pathway components) cannot be considered conclusive without validation using genetic mutants. We therefore performed additional experiments using available mutant strains for components of the BH4 recycling

pathway and BH4-dependent enzymes. Regarding the BH4 recycling pathway, a previous study demonstrated that this pathway is not required to maintain steady-state BH4 levels but only becomes rate-limiting under conditions of diminished BH4 synthesis, such as in reduction-of-function *cat-4* mutant worms (PMID: 25808955). We have now clarified this point in the main text. In addition, we performed lifespan analyses using *pcbd-1* and *qdpr-1* mutant strains. Consistent with our RNAi results, *pcbd-1* and *qdpr-1* mutants were not short-lived at 15 °C relative to wild-type animals (**Extended Data Fig. 5c,d**), supporting the conclusion that the recycling pathway is not required for cold-induced longevity under physiological conditions.

Besides *pah-1*, we have now performed experiments using loss-of-function mutants for the other BH4-dependent enzymes. In contrast to *pah-1* mutants, *cat-2*, *tph-1*, and *agmo-1* mutants did not exhibit reduced lifespan at cold temperature (**Extended Data Fig. 9a**), further supporting a specific role for PAH-1–dependent BH4 utilization in cold-induced longevity.

3. It is unclear throughout the narrative of the manuscript why cat-4 expression increases at lower temperatures. Are there any specific transcription factor that contributes to the expression of cat-4? This point at least should be discussed to explain the mechanism of increased cat-4 expression at moderately lower temperatures. It would also be helpful to clarify whether this induction is predominantly observed in muscles or occurs more broadly across tissues in response to cold temperature.

We thank the Reviewer for this important suggestion regarding the regulation of *cat-4* expression. To address this point, we have now assessed whether cold temperature promotes *cat-4* expression at the transcriptional level and examined candidate transcriptional regulators involved in cold-induced longevity. First, we performed qRT–PCR analysis and found that exposure to 15 °C significantly increased *cat-4* mRNA levels compared to 20 °C, indicating that CAT-4 induction occurs, at least in part, through transcriptional regulation (**Extended Data Fig. 3a**). Second, we tested two established regulators of cold-induced longevity in *C. elegans* (PMID: 31408455, 23415228). We found that knockdown of *mdt-15*, a subunit of the evolutionary conserved Mediator complex and a key transcriptional regulator of metabolic responses, significantly reduced *cat-4* expression at 15 °C (**Extended Data Fig. 3b**). In contrast, knockdown of *daf-16* did not significantly decrease *cat-4* expression (**Extended Data Fig. 3b**). Together, these findings indicate that cold temperature increases *cat-4* expression at the transcriptional level and identify MDT-15 as a contributor to this regulation, suggesting that elevated CAT-4 levels are part of a regulated transcriptional program activated at low temperature.

We apologize for not making more clear in the initial submission whether CAT-4 induction occurs in tissues other than muscle. We observed that CAT-4 is highly expressed in the anterior intestine at standard temperature, and that cold exposure

further increases its expression in this region. In contrast, CAT-4 expression in muscle was barely detectable at 20 °C but was also robustly induced by cold temperature. We have now reorganized the text and figures to present these findings more clearly and to explicitly highlight cold-induced CAT-4 expression in both tissues. Importantly, tissue-specific knockdown experiments allowed us to determine which tissue is primarily responsible for the elevated BH2 synthesis that contributes to cold-induced longevity. Specifically, knockdown of *cat-4* in muscle shortened lifespan at cold temperature, whereas knockdown in other tissues, including the intestine, had no effect. The text now says: "*To determine the tissues in which CAT-4 is expressed, we generated a fluorescent reporter that enables independent translation of CAT-4 and GFP from a single bicistronic pre-mRNA transcribed under the endogenous* cat-4 *promoter. Knockdown of cat-4 reduced GFP signal, validating the reporter (**Extended Data Fig. 6a**). At standard temperature, CAT-4 was mostly expressed in the anterior intestine, and cold exposure further increased CAT-4 levels in this region (**Extended Data Fig. 6a,b**). In contrast to the intestine, CAT-4 was barely detectable in muscle at 20 °C (**Fig. 2a**). However, cold temperature triggered robust expression of cat-4 in the muscle, an effect abolished by cat-4 RNAi treatment (**Fig. 2a,b**). To assess whether elevated BH2 synthesis in these tissues promotes organismal longevity at cold temperature, we performed tissue-specific knockdown experiments. Notably, knockdown of cat-4 in the muscle alone reduced cold-induced longevity, whereas knockdown in the intestine had no effect on lifespan (**Fig. 2c,d**). Likewise, knockdown of cat-4 in neurons, epidermis, or germline did not shorten lifespan at 15 °C (**Fig. 2e-g**)*".

In addition, we have now further strengthened this conclusion using AID strains, as we observed that muscle-specific degradation of CAT-4 after development is sufficient to fully phenocopy the lifespan-shortening effects of ubiquitous somatic CAT-4 depletion at 15 °C (**Extended Data Fig. 6e**). Together, these results identify muscle as a critical tissue in which elevated BH2 synthesis promotes cold-induced longevity.

4. In Figure 3, the authors showed that RNAi knockdown of ent, which encodes BH2 transporter, decreased the lifespan of animals at 15°C. To strengthen the statement that ENT contributes to BH2 transport at cold temperature, the authors should measure the lifespan of ent mutant animals with muscle-specific knockdown of cat-4 and neuron-specific knockdown of dhfr-1. Alternatively, examining whether knockdown of ent masks lifespan-increasing effect of cat-4 overexpression will reinforce the cell non-autonomous role of BH2.

We have performed both experiments suggested by the Reviewer. First, we obtained the *ent-1(ok743)* mutant strain (RB891) from the CGC, outcrossed it four times to the N2 wild-type background, and then crossed it into neuron-specific and muscle-specific RNAi strains. We found that *ent-1* mutant worms are short-lived at cold temperature, and neither muscle-specific knockdown of *cat-4* nor neuron-specific

knockdown of *dhfr-1* further reduced their lifespan (**Extended Data Fig. 6g,h**). These data strengthen our conclusion that ENT contributes to BH2 transport at cold temperature.

In addition, experiments using our CAT-4 overexpression model further support a cell non-autonomous role for BH2. We found that neuronal-specific downregulation of either BH2 uptake (via *ent-1* RNAi) or its conversion to BH4 through the salvage pathway (via *dhfr-1* RNAi) significantly reduces the lifespan extension conferred by muscle-specific *cat-4* overexpression at standard temperature (**Fig. 3g**). In contrast, neuronal-specific knockdown of *ent-1* or *dhfr-1* had no effect on lifespan in control animals at this temperature (**Extended Data Fig. 8m**). Collectively, these results support a model in which elevated BH2 production in muscle promotes longevity in a cell non-autonomous manner and requires both BH2 uptake and its conversion into bioactive BH4 in distal tissues, such as neurons.

5. Although the authors demonstrate that tyrosine is required and sufficient for longevity, the downstream molecular pathways remain unclear. The authors should consider examining whether cold-induced transcriptomic datasets (e.g., GEO: GSE62297) reveal enrichment of tyrosine metabolism–related or established longevity-associated gene signatures. This would clarify whether tyrosine acts through a defined regulatory program rather than general metabolic support and would strengthen the mechanistic depth of the study.

The Reviewer is absolutely right that the downstream mechanisms by which tyrosine promotes longevity were not addressed in our initial submission. We are very grateful for this insightful suggestion and for pointing us to the published transcriptomic dataset (GEO: GSE62297; PMID: 26027928). We have now performed Over-Representation Analysis (ORA) of differentially abundant transcripts in this dataset, which compares day-3 adult worms at 15 °C and 25 °C. This analysis identified significant enrichment of biological processes related to neurogenesis, neuron development, ribosome biogenesis, and muscle structure development (**Figure R2**). While these results provide valuable insight into the transcriptional remodeling induced by cold temperature, they did not reveal a clear tyrosine-related gene signature that could readily explain the longevity effects observed in our study.

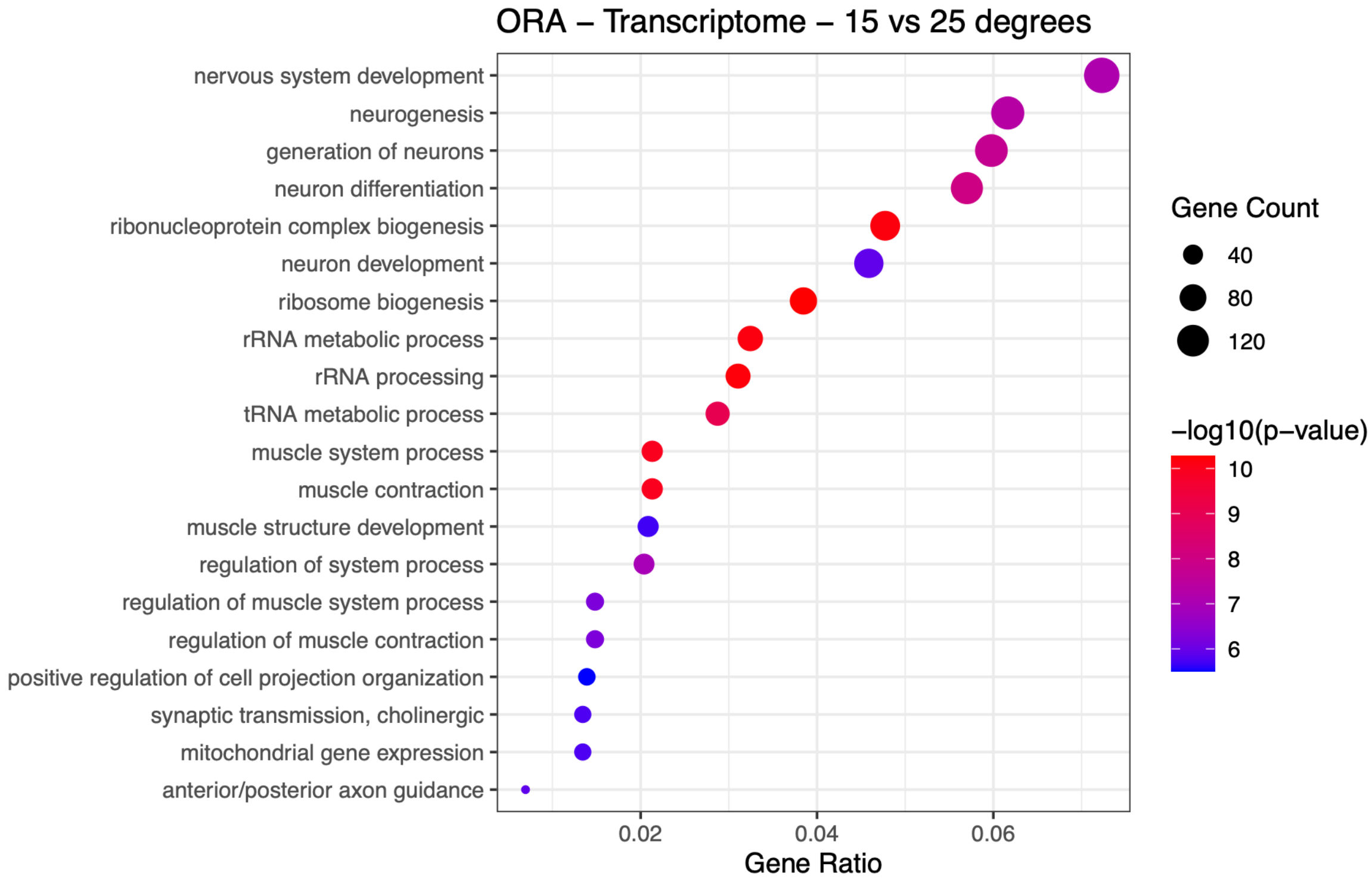

Figure R2. Over-representation analysis (ORA) of differentially abundant transcripts from a previously published microarray dataset comparing day-3 adult *C. elegans* at 15 °C and 25 °C (PMID: 26027928). The most significantly enriched biological processes are shown.

To gain further mechanistic insight, we analyzed our quantitative proteomics datasets (**Supplementary Table 5**). Specifically, we performed ORA using proteins differentially abundant in day-5 adult worms at 15 °C relative to either 20 °C or 25 °C. Notably, among the biological processes most consistently upregulated at cold temperature were small-molecule metabolic processes and amino acid metabolic processes (**Extended Data Fig. 10a,b**). Within these pathways, we identified increased abundance of 4-hydroxyphenylpyruvate dioxygenase (HPD-1) (**Extended Data Fig. 10c**), a key enzyme in the canonical tyrosine catabolic pathway that ultimately generates acetoacetate and fumarate, thereby linking tyrosine metabolism to central carbon metabolism (PMID: 15581571). Importantly, re-analysis of the transcriptomic dataset suggested by the Reviewer (GEO: GSE62297) also revealed increased *hpd-1* expression at cold temperature (**Extended Data Fig. 10c**). Prompted by these findings, we performed knockdown experiments and found that loss of *hpd-1* significantly reduced cold-induced longevity (**Extended Data Fig. 10d**), supporting a functional contribution of tyrosine catabolism to lifespan extension at low temperature. Although *hpd-1* knockdown also caused a slight reduction in lifespan at 20 °C and 25 °C, these effects did not reach statistical significance (**Extended Data Fig. 10d**).

In addition, we have now performed quantitative proteomics following tyrosine supplementation at both 15 °C and 20 °C (**Supplementary Table 6**). These analyses revealed 54 commonly upregulated proteins and 42 commonly downregulated proteins across both temperatures (**Extended Data Fig. 10e,f**). ORA of differentially

abundant proteins identified significant enrichment for proteins involved in lipid metabolic processes and enzyme regulator activity (**Extended Data Fig. 10g,h**), indicating that elevated tyrosine levels induce changes in regulated pathways.

Together, these data provide mechanistic insight into the downstream effects of increased tyrosine levels. While additional studies will be required to fully define the programs involved, our results support a model in which tyrosine promotes longevity through specific regulatory and metabolic pathways, including HPD-1-dependent tyrosine catabolism.

6. In the human cell experiments (Fig. 7), aggregation is reduced without changes in total protein levels. The authors should clarify whether proteasome or autophagy pathways are altered by BH2 or tyrosine treatment to exclude indirect proteostasis effects.

We thank the Reviewer for this important suggestion. In the revised manuscript, we have now directly addressed whether BH2 and tyrosine alter proteasome or autophagy pathways in human cells. Specifically, we performed new experiments in HEK293 cells and found that both BH2 and tyrosine treatment lead to a statistically significant, albeit modest, increase in trypsin-like proteasome activity (**Extended Data Fig. 12a,b**). In contrast, neither treatment increased LC3 lipidation, a marker of autophagosomes (**Extended Data Fig. 12c,d**).

Because BH2 treatment reduced aggregation of disease-associated proteins without significantly altering their total levels (**Fig. 6a–c**), we hypothesized that additional protein quality control mechanisms contribute to these protective effects. Consistent with our findings in *C. elegans* (**Extended Data Fig. 11b**), knockdown of the TRiC/CCT chaperonin subunit CCT2 abolished the ability of BH2 to suppress pathological protein aggregation, identifying TRiC/CCT-dependent folding capacity as a key contributor to BH2-dependent proteostasis enhancement in human cells (**Extended Data Fig. 12e**).

Together with the new *C. elegans* experiments added in response to Reviewer #2 (please see **Extended Data Fig. 11a-g**), in which CAT-4 activity similarly engages both proteasome and TRiC/CCT chaperonin pathways, these findings support a conserved process whereby BH2 enhances proteostasis capacity through distinct mechanisms, with an important contribution from TRiC/CCT-mediated folding capacity in preventing pathological protein aggregation.

Minor comments

1. In the Abstract and Introduction, BH2 is described as inactive yet central; the authors should more precisely distinguish between BH2 as a transport reservoir and BH4 as the enzymatically active cofactor.

We have revised the Abstract and Introduction to more clearly distinguish BH2 as a transportable intermediate and reservoir for BH4, and BH4 as the enzymatically active cofactor.

The Abstract now says: "*Cold temperature upregulates the biopterin-synthesis enzyme GCH1/CAT-4, increasing production of dihydrobiopterin (BH2), a transportable reservoir for the bioactive cofactor tetrahydrobiopterin (BH4)". (…) "Together, these findings identify BH2 as a cold-induced metabolite whose synthesis, intertissue transport, and conversion into bioactive BH4 preserve organism-wide proteostasis and extend lifespan*".

The Introduction now says: "*Here, we identify dihydrobiopterin (BH2) as a cold-induced metabolite whose synthesis, intertissue transport and conversion into the bioactive enzymatic cofactor BH4 constitute a multi-tissue regulatory system that promotes longevity and maintains proteostasis across tissues. Through metabolomic, genetic and physiological analyses, we find that elevated BH2 synthesis in muscle and its conversion to BH4 in distal tissues promotes tyrosine synthesis, thereby mediating key aspects of cold-induced longevity*".

We have also rewritten the results section to make these points more clear. For instance, the text now says: "*Although BH2 is an inactive metabolite, it functions as a key intermediate in the synthesis and intertissue transport of its reduced, bioactive form, BH4*[23,24]*. Because BH4 is chemically unstable and readily oxidized, the more stable BH2 form serves as a major organismal reservoir of this bioactive metabolite*[23,24]".

2. The metabolomics criteria in Fig. 1 are stringent. However, the authors should provide a clearer rationale for prioritizing BH2 over the other 63 cold-induced metabolites and include a more detailed comparison of effect sizes and reproducibility across independent datasets.

We apologize for not clearly explaining in the original submission the rationale for prioritizing BH2. Our decision was based on several observations. First, among the 45 reproducible cold-induced metabolites, five are associated with biopterin metabolism. Second, BH2 was among the most strongly upregulated metabolites at cold temperature (**Fig. 1a,b**). Third, and most importantly, quantitative proteomics identified CAT-4, the first and rate-limiting enzyme in BH2 synthesis, as one of the most upregulated proteins under cold conditions (**Fig. 1f,g and Supplementary Data 5**). Together, these findings indicated that BH2 synthesis is actively regulated and highlighted BH2 as a relevant candidate for further investigation. We have now clarified this rationale in the manuscript. The revised text now reads: "*Notably, we identified several metabolites associated with tetrahydrobiopterin (BH4) synthesis and its function as an enzymatic cofactor, including GTP, dihydrobiopterin (BH2), tryptophan, phenylalanine, and tyrosine (**Fig. 1a-c**). Because BH2 was among the most strongly upregulated metabolites at cold temperature (**Fig. 1a,b**), we focused*

*subsequent analyses on this metabolite". (…) "We next asked whether BH2 reflects a regulated pathway rather than a passive metabolic consequence of reduced temperature. Using quantitative proteomics, we identified GTP cyclohydrolase I (GCH1 in humans, CAT-4 in C. elegans), the first and rate-limiting enzyme in BH2 synthesis[22-24], as one of the most strongly upregulated proteins at cold temperature compared to standard and warm temperatures (**Fig. 1f,g and Supplementary Table 5**)". (…) "These findings indicate that cold-induced CAT-4 expression is part of a regulated transcriptional program, prompting us to examine whether elevated BH2 synthesis contributes to longevity*".

We have also included a more detailed assessment of effect sizes and reproducibility across independent experiments as requested by the Reviewer. For instance, we have now performed Pearson correlation analyses between the two independent metabolomics experiments and added corresponding scatter plots (**Extended Data Fig. 1a**). These analyses revealed strong positive correlations (r = 0.90, $P = 1.9 \times 10^{-37}$ for 15 °C versus 20 °C; r = 0.87, $P = 1.4 \times 10^{-50}$ for 15 °C vs 25 °C), indicating high concordance between datasets despite being generated independently by different researchers and at different times.

In the initial experiment, 64 metabolites met the cutoff criteria (FDR-adjusted *P*-value < 0.01 for both 15 °C vs 20 °C and 15 °C vs 25 °C comparisons). Of these, 45 were reproducibly increased in the second experiment using the same criteria. We have clarified this in the main text and added new visualizations, including a Venn diagram to illustrate the overlap (**Extended Data Fig. 1b**) and a dot-plot comparing log2 fold changes and FDR-adjusted *P*-values across datasets (**Extended Data Fig. 1e**), demonstrating consistent effect sizes and reproducibility. The text now says: "*To identify metabolites induced by cold temperature, we applied the following cutoff criteria: (A) metabolite levels were higher at 15 °C (cold) than at 20 °C (standard); (B) after correction for multiple testing, the false discovery rate (FDR)-adjusted P-value was less than 0.01; and (C) the cold-induced changes relative to standard temperature were also significant when comparing 15 °C with 25 °C (warm). Based on these criteria, we identified 64 metabolites whose levels significantly increased at cold temperature (**Fig. 1a,b**). To further validate these findings, we performed an independent metabolomics experiment (**Supplementary Table 2**). Pearson correlation analysis of $\log_2$ fold changes between the two experiments revealed a strong positive correlation, indicating concordance in temperature-dependent metabolite changes (**Extended Data Fig. 1a**). Applying the same cutoff criteria as above, 45 metabolites were consistently increased at cold temperature across both experiments, defining a core set of cold-induced metabolites (**Extended Data Fig. 1b-d**). Further analysis confirmed that these shared cold-induced metabolites exhibit comparable effect sizes across independent experiments (**Extended Data Fig. 1e**), supporting the robustness of the observed changes*".

3. The authors showed that aging and cold temperature altered the levels of BH4 using

ELISA. Measuring BH4 or BH2 levels in cat-4-overexpressing animals at various temperatures would strengthen the data using cat-4 overexpression.

We thank the Reviewer for suggesting this important experiment to strengthen our conclusions. We have now measured BH4 levels in worms overexpressing *cat-4* specifically in the muscle at 15 °C, 20 °C, and 25 °C. We observed that muscle-specific *cat-4* overexpression increases organismal levels of bioactive BH4 at all three temperatures (please see **Extended Data Fig. 8f–h**).

4. In Fig. 1, the cutoff criteria are described in the Results; these thresholds should also be clearly stated in the figure legend for transparency.

We have now clearly stated the cutoff criteria in the figure legend. Specifically, the legend now says: *"In **a-b**, Volcano plots show the $\log_2$ fold change plotted against the negative decadic logarithm of the false discovery rate (FDR)-adjusted P-value (q value), calculated using the two-stage step-up method of Benjamini, Krieger, and Yekutieli multiple t-test (**Supplementary Table 1**). Red dots indicate the 45 cold-induced metabolites that met all of the following cutoff criteria: (1) increased at 15 °C relative to 20 °C (FDR < 0.01); (2) increased at 15 °C relative to 25 °C (FDR < 0.01); and (3) reproducibly increased in an independent experiment using the same criteria (**Supplementary Table 2**)*".

5. In Fig. 1a, the labeling/annotation for BH2 is difficult to interpret because it is not visually clear which specific feature or dot corresponds to BH2; adding a clearer label, arrow placement, or an inset/legend mapping would substantially improve figure readability for non-specialist readers.

We thank the Reviewer for this helpful suggestion. We have improved the annotation of BH2 in **Fig. 1a,b** and **Extended Data Fig. 1c,d** by adding arrows to clearly indicate the corresponding dots.

6. The narrative of this paper underscores the enzymes CAT-4, DHFR-1, and PCBD-1, visualized in the pathway in figure 1c. Although the functions of these enzymes are introduced as rate-limiting steps in the main text, such emphasis is not present in the figure. I therefore suggest that the authors stress the central enzymes in the figure panel.

We have now revised **Fig. 1c** to emphasize the key enzymes in the pathway in the context of cold-induced longevity, thereby better aligning with the narrative and improving readability. Specifically, CAT-4 and DHFR-1, which are rate-limiting for BH2 synthesis and its conversion to BH4, respectively, are now highlighted, as they are required for cold-induced longevity. In addition, we have emphasized PAH-1, the BH4-dependent enzyme identified in our study as functionally relevant for cold-induced longevity.

Although PCBD-1 and QDPR-1 function in BH4 recycling, our data indicate that this pathway is not required for cold-induced longevity. We have therefore not highlighted these enzymes in the figure to avoid overstating their role in the context of our results. The figure legend has been updated to make clear that the highlighted enzymes are those functionally implicated in cold-induced longevity.

7. Figure citation strings appear to be repeated verbatim in close proximity in several places (e.g., "Fig. 1a,b and Supplementary Fig. 1a–c" repeated twice), and the authors may consider reducing redundancy by citing the full set once and then using a shorter reference thereafter, or by retaining supplementary citations only where the supplementary panels add non-overlapping evidence that is explicitly discussed in that sentence.

We have now reduced redundancy in figure citations by citing the full set once and subsequently referring only to the main figures, unless supplementary figures or tables provide additional non-overlapping evidence that is explicitly discussed.

8. In figure 3f and g, scale bars are missing.

We thank the Reviewer for identifying this oversight. We apologize that the scale bars were not clearly visible in the initial submission. We have now increased their visibility (these figures are now presented as **Fig. 2a and 2b**).

9. Regarding Figure 4, I recommend that the authors explain why they focused on the aggregation of IFB-2 in detail to improve readers' understanding.

We have now clarified the rationale for focusing on IFB-2 aggregation as a readout to assess cell non-autonomous effects of BH2 synthesis in muscle. The text now says: "*To further examine the cell non-autonomous effects of BH2, we focused on the intestine, a tissue that progressively loses structural integrity during aging*[27,28]*. To this end, we assessed the intermediate filament IFB-2 because this protein is expressed exclusively in the intestine and undergoes age-related changes that can be readily monitored*[28,29]*. In young animals, IFB-2 is confined to the apical cytoplasm of intestinal cells. With age, IFB-2 levels increase and the protein becomes mislocalized throughout the cytoplasm, where it forms aggregates that contribute to intestinal decline*[28]*. We found that cold temperature prevents IFB-2 mislocalization and aggregation during aging (**Extended Data Fig. 7a**). However, loss of cat-4 abolished these protective effects in the intestine (**Extended Data Fig. 7a**). Notably, muscle-specific knockdown of cat-4 was sufficient to block the cold-induced suppression of intestinal IFB-2 aggregation (**Extended Data Fig. 7b**). Collectively, these findings indicate that cold-induced BH2 synthesis in muscle exerts cell non-autonomous effects, such as preserving intestinal integrity during aging". (…) "Consistent with a systemic protective effect, muscle-specific cat-4 overexpression prevented age-*

*related accumulation and aggregation of the intestine-specific protein IFB-2 at standard temperature (**Fig. 3d and Extended Data Fig. 8l**). As IFB-2 aggregation contributes to loss of intestinal integrity and subsequent bacterial colonization during aging[28], we examined whether CAT-4 prevents this deleterious process. Indeed, muscle-specific cat-4 overexpression reduced bacterial invasion of the intestine in aged worms at standard temperature (**Fig. 3e,f**)*".

10. In Supplementary Figure 4b, the authors seem to have compared wild-type strain and him-8(e1489); cat-4(e3015). Are there any rationales using double mutants rather than cat-4(e3015)? Furthermore, mutant animals used in lifespan assays in Supplementary Figure 4b and Supplementary Figure 8 does not seem to be backcrossed to WT. The authors should clarify these points.

We apologize for not clearly describing these details in the original submission. Indeed, we used the double mutant *him-8(e1489)*; *cat-4(e3015)* strain in our initial submission (now **Extended Data Fig. 3c**). The *him-8* mutation is commonly used as a genetic tool to increase male frequency and facilitate crossing. All lifespan experiments were performed using hermaphrodites. However, we cannot exclude the possibility that the *him-8* background may influence lifespan. To address this concern, we have now performed lifespan analyses using two independent strains carrying mutations only in *cat-4* (i.e., loss-of-function alleles *cat-4(ok342)* and *cat-4(tm773)*). Similar to the *him-8(e1489); cat-4(e3015)* double mutants, both *cat-4(ok342)* and *cat-4(tm773)* mutant worms were short-lived at standard temperature (**Extended Data Fig. 3d,e**), supporting that loss of *cat-4* reduces lifespan independently of the *him-8* mutation.

All mutant strains used in this study have been backcrossed multiple times to the N2 wild-type background. This information has now been added to the Methods section, including the number of outcrosses for each strain.

Referee #2 (Remarks to the Author):

The manuscript by Lee et al explores how moderate cold temperatures activate a metabolite-driven intertissue signaling network that promotes longevity and proteostasis, focusing on the role of dihydrobiopterin (BH2) and its bioactive form tetrahydrobiopterin (BH4).

Moderate cold temperatures (15°C) extend lifespan in Caenorhabditis elegans by upregulating the enzyme GCH1/CAT-4 in muscle cells, leading to increased BH2 production. BH2 is transported to distal tissues (intestine, neurons, germline), where it is converted into BH4, a cofactor for enzymes like phenylalanine hydroxylase (PAH-1), which enhances tyrosine synthesis and contributes to longevity. Interestingly, BH2 synthesis occurs in muscle cells, but its benefits are distributed systemically. BH2 is transported to other tissues via nucleoside transporters (e.g.,

ENT1-ENT7 paralogs) and converted into BH4 by DHFR-1 in neurons, intestine, and germline. Muscle-specific overexpression of CAT-4 extends lifespan and suppresses protein aggregation at both cold and standard temperatures, demonstrating the central role of muscle in generating systemic pro-longevity signals.

Elevated BH2 levels prevent age-related protein aggregation and neuronal dysfunction. Muscle-derived BH2 suppresses pathological protein aggregation in distal tissues, including neurons and the intestine, preserving tissue integrity and function during aging. The authors go on to show that BH2 supplementation in human cultured cells can also protect from PolyQ repeat proteins as well as disease variants toward ALS.

When I first read the title and abstract of this manuscript, several key questions came to mind. First, does this work in tpa1 (-) animals? Can they identify the transporters to formally show that BH2 mad. Can they identify the biosynthetic enzymes to make BH4 in distal tissues? Is production of BH2 sufficient for all of the lifespan and proteostasis effects? What does BH4 affect downstream to elicit these positive effects? Finally, what is the tradeoff?

We thank Reviewer #2 for the careful evaluation of our work and for the constructive comments and suggestions. We have now addressed all of the Reviewer's comments. We believe that the additional experiments and analyses have significantly strengthened the conclusions of the manuscript and improved both its mechanistic depth and overall clarity.

In short order, almost every single one of these questions has been addressed and highlighted below are some remaining suggestions:

1) While the authors show that BH4 works on PAH-1 to elicit the effects in distal tissues, this does not fully explain the increased proteostasis capacity. Do the authors have any data or suggestions on how PAH-1 can affect proteostasis? Is this HSF1 dependent? What is the program that is increased by BH4 that is dependent upon PAH-1?

We thank the Reviewer for this important comment and completely agree that the mechanism by which elevated CAT-4 activity and subsequent BH2/BH4/tyrosine synthesis enhances proteostasis was insufficiently addressed in the original submission. To directly address this question, we have now performed multiple experiments to identify downstream proteostasis mechanisms.

We found that both the proteasome activator PSME-3 and the TRiC/CCT chaperonin complex contribute to the enhanced proteostasis capacity conferred by muscle-specific *cat-4* overexpression. Specifically, knockdown of *psme-3* or the TRiC/CCT subunit *cct-2* attenuated the ability of muscle-specific *cat-4* overexpression to suppress neuronal polyQ67 aggregation (**Extended Data Fig. 11a,b**). In contrast, knockdown of *fkb-6*, a cold-induced HSP90 co-chaperone, had no effect (**Extended**

Data Fig. 11c). We also tested *hsf-1* and found that loss of this transcription factor partially attenuated the protective effects of muscle-specific *cat-4* overexpression (**Extended Data Fig. 11d**), although to a lesser extent than *cct-2* or *psme-3* knockdown.

To determine whether PAH-1 contributes to activation of these proteostasis pathways, we examined the effects of *pah-1* loss on cold-induced proteostasis. Notably, loss of *pah-1* attenuated the cold-induced upregulation of *cct-2* and *psme-3* expression, as well as the increase in trypsin-like proteasome activity (**Extended Data Fig. 11e-g**). Collectively, these results indicate that elevated CAT-4 activity and the subsequent BH4-dependent synthesis of tyrosine by PAH-1 enhance proteostasis through several downstream mechanisms, including increased proteasome activity and TRiC/CCT chaperonin function.

The text now says: "*We next asked how elevated CAT-4 activity enhances proteostasis capacity. Cold temperature induces expression of the proteasome activator PA28γ/PSME-3, thereby increasing trypsin-like proteasome activity[10]. We found that knockdown of psme-3 attenuates the capacity of muscle-specific cat-4 overexpression to suppress polyQ67 aggregation in neurons at standard temperature (**Extended Data Fig. 11a**). Although loss of psme-3 increased polyQ aggregation in cat-4-overexpressing worms, it did not alter total polyQ levels (**Extended Data Fig. 11a**), suggesting a broader defect in proteostasis rather than impaired degradation of polyQ proteins per se. Thus, CAT-4 activity appears to influence proteostasis through additional mechanisms. In addition to proteasome activity, cold temperature induces expression of different subunits of the TRiC/CCT complex[11], a chaperonin that prevents polyQ-expanded aggregation[36-38]. Knockdown of the TRiC/CCT subunit cct-2 completely abolished the protective effects of muscle-specific cat-4 overexpression on neuronal polyQ67 aggregation at standard temperature (**Extended Data Fig. 11b**). In contrast, knockdown of fkb-6, a cold-induced co-chaperone of HSP90[11], had no effect on polyQ67 aggregation (**Extended Data Fig. 11c**). We also tested hsf-1, a key transcription factor that protects against protein misfolding and aggregation[39]. Loss of hsf-1 partially attenuated the protective effects of muscle-specific cat-4 overexpression (**Extended Data Fig. 11d**), although less strongly than cct-2 or psme-3 knockdown. To determine whether PAH-1 activity contributes to activation of these mechanisms, we examined the effects of pah-1 loss on cold-induced proteostasis. Notably, loss of pah-1 attenuated the cold-induced upregulation of cct-2 and psme-3 expression, as well as the increase in trypsin-like proteasome activity (**Extended Data Fig. 11e-g**), indicating that PAH-1 is required for activation of key proteostasis pathways at cold temperature. Together, these results suggest that elevated CAT-4 activity and subsequent BH4-dependent tyrosine synthesis enhance proteostasis through several downstream mechanisms, including increased proteasome activity and TRiC/CCT chaperonin function*".

2) The experiment to show that PAH-1 is downstream of BH4 is compelling, but the negative data of the other 3 BH4 binding proteins needs to be done with mutants and not RNAi to ensure that their involvement is not being missed.

We thank the Reviewer for this important comment. We have now performed experiments using loss-of-function mutants for the other BH4-dependent enzymes (i.e. *cat-2*, *tph-1*, and *agmo-1*) (please see **Extended Data Fig. 9a**). In contrast to *pah-1* mutants, these mutants did not exhibit reduced lifespan at cold temperature. The text now says: "*To determine how BH4 utilization as an enzymatic cofactor contributes to cold-induced longevity, we performed a targeted RNAi screen against the four BH4-dependent enzymes expressed in C. elegans: phenylalanine hydroxylase (PAH-1), tryptophan hydroxylase (TPH-1), tyrosine hydroxylase (CAT-2), and alkylglycerol monooxygenase (AGMO-1)*[25]*. Among them, knockdown of pah-1 after development shortened lifespan, whereas knockdown of the other BH4-dependent enzymes did not affect cold-induced longevity (**Fig. 4a**). Consistently, loss-of-function mutants of tph-1, cat-2, and agmo-1 mutants were not short-lived, whereas pah-1 mutants exhibited reduced longevity at cold temperature (**Extended Data Fig. 9a,b**)*".

3) The authors show that the cold induced lifespan that requires BH2 is not daf-2 dependent, but it seems that other longevity pathways that are directly related to metabolism could be overlapping, such as the ETC derived pathway or even calorie restriction. Have these been tried?

We thank the Reviewer for this important suggestion. To address this point, we examined one model of reduced mitochondrial electron transport chain activity (*isp-1* mutant worms) and two models of reduced food intake (*eat-2* and *phm-2* mutant worms). Importantly, knockdown of *cat-4* after development shortened lifespan not only at 15 °C but also at standard temperature in these models (**Extended Data Fig. 4c–h**). Thus, these experiments led to the relevant finding that CAT-4 activity contributes to lifespan extension in other longevity paradigms beyond cold temperature. The manuscript has been revised accordingly. Specifically, the text now says: **"***Prompted by these findings, we next asked whether CAT-4 activity also contributes to lifespan extension in other longevity paradigms. We found that cat-4 knockdown after development in mutant worms with reduced insulin signaling did not affect their extended lifespan at standard temperature, but only shortened lifespan at 15 °C (**Extended Data Fig. 4a,b**). In contrast, loss of cat-4 shortened lifespan in long-lived worms with reduced mitochondrial electron transport chain activity or reduced food intake at both cold and standard temperatures (**Extended Data Fig. 4c-h**). These results suggest that CAT-4 activity contributes to other longevity paradigms beyond cold temperature*".

4) Might have missed this, but does overexpression of cat-2 extend lifespan in TRPA(-) animal?

We thank the Reviewer for suggesting this interesting experiment. We have now generated loss-of-function *trpa-1* mutant worms overexpressing *cat-4* specifically in the muscle. Notably, muscle-specific *cat-4* overexpression extended lifespan in *trpa-1* mutants at 15 °C, 20 °C, and 25 °C (please see **Extended Data Fig. 8i-k**). The text now says: "*Remarkably, overexpression of cat-4 in the muscle alone was sufficient to increase organismal levels of bioactive BH4 and extend lifespan not only at 15 °C, but also at 20 °C and 25 °C (**Fig. 3b,c and Extended Data Fig. 8f-h**). Moreover, muscle-specific cat-4 overexpression also extended lifespan in loss-of-function trpa-1 mutant worms across different temperatures (**Extended Data Fig. 8i-k**). These findings indicate that increasing CAT-4 activity in muscle promotes longevity independently of both temperature and the cold-sensitive channel TRPA-1*".

In summary, the research identifies BH2/BH4 signaling as a central mechanism through which moderate cold temperatures promote longevity and systemic proteostasis, with potential applications for combating age-related diseases in humans. The findings as the stand are well done and easily interpretable. The impact of this work could have potential, unknown, positive benefits on diseases of proteostasis,

Referees' comments:

Referee #1 (Remarks to the Author):

The authors addressed my previous concerns successfully.

Referee #2 (Remarks to the Author):

Thank you for addressing all of my concerns.

We thank the Reviewers for the positive assessment of our revised manuscript and for the constructive feedback provided during the initial review, which helped us improve the manuscript.
