# Supplementary material for: Cold activates a BH2 metabolic network promoting longevity and proteostasis
Source: Nature. 2026 Oct 21;659(8140):480–9. doi: 10.1038/s41586-026-11152-9 (PMC13754623; doi:10.1038/s41586-026-11152-9)

nature portfolio

https://doi.org/10.1038/s41586-026-11152-9

Supplementary information

Cold activates a BH2 metabolic network promoting longevity and proteostasis

In the format provided by the authors and unedited

Fig. 3c

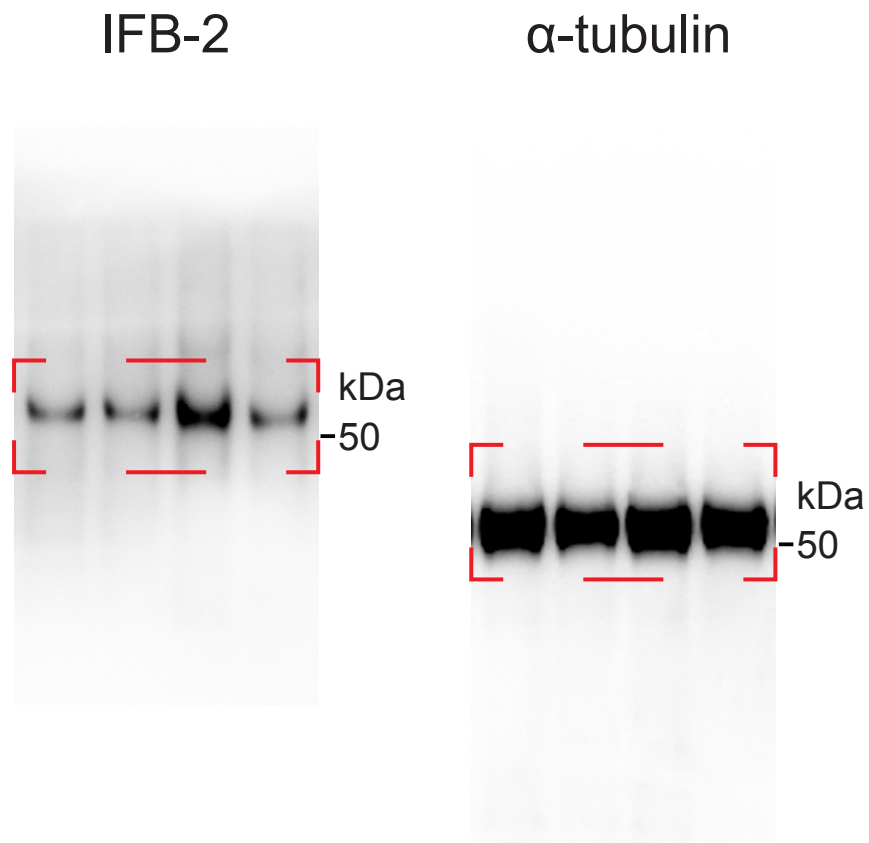

Fig. 4b

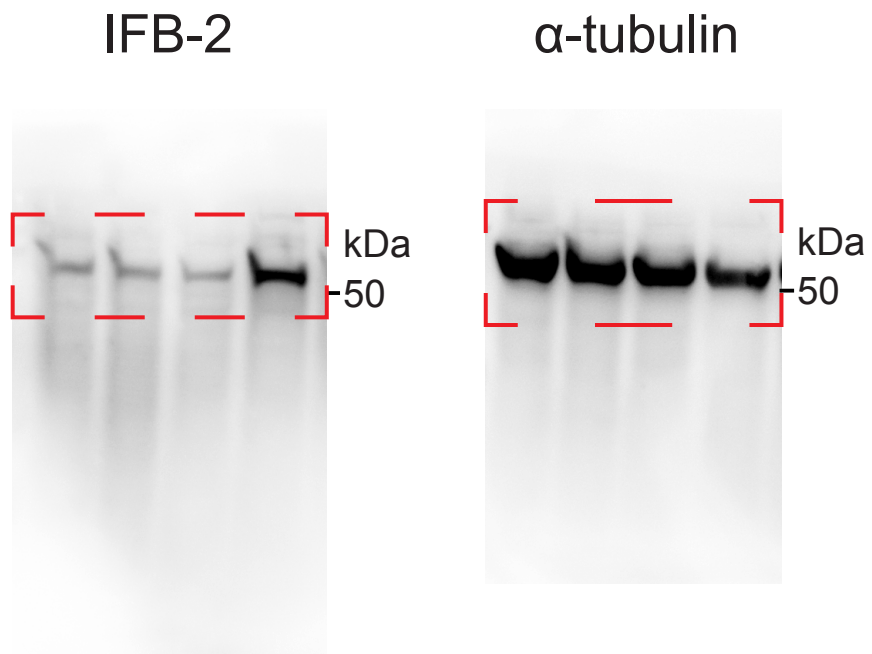

Fig. 4c

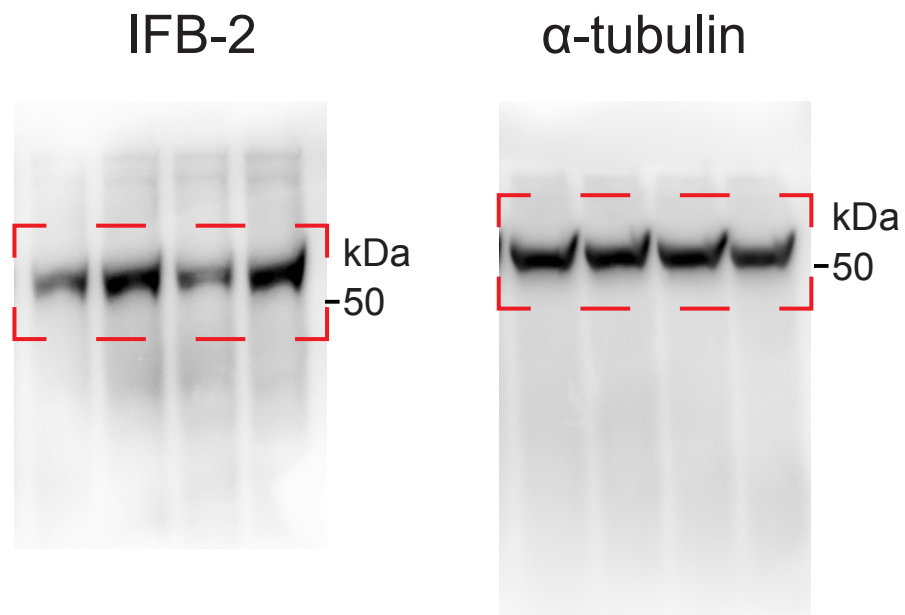

Fig. 5a

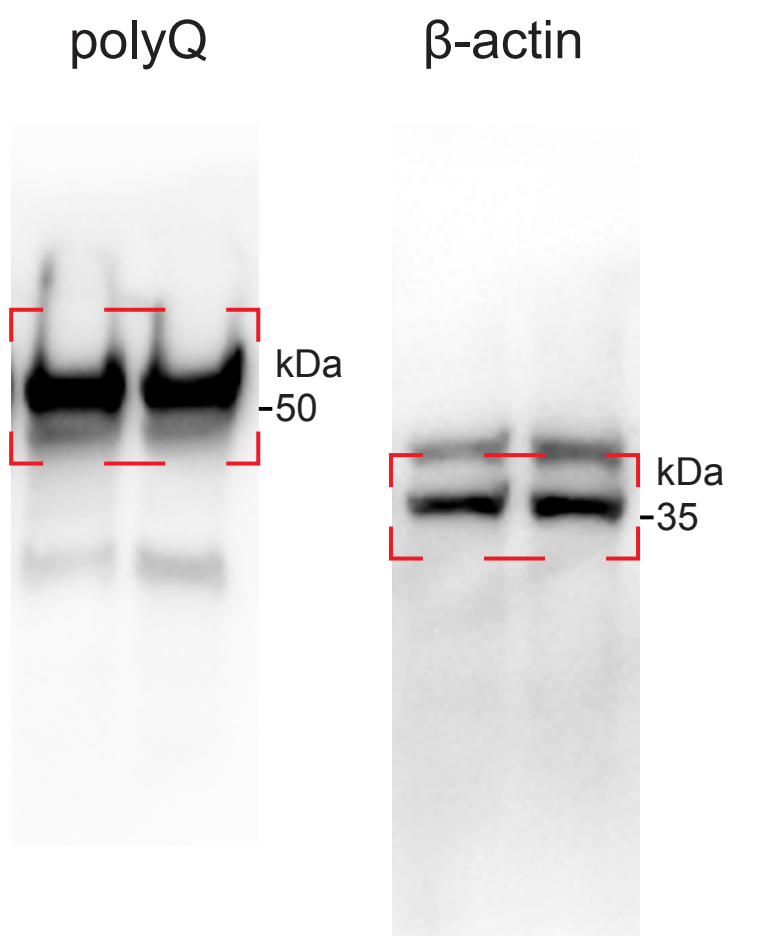

Fig. 5b

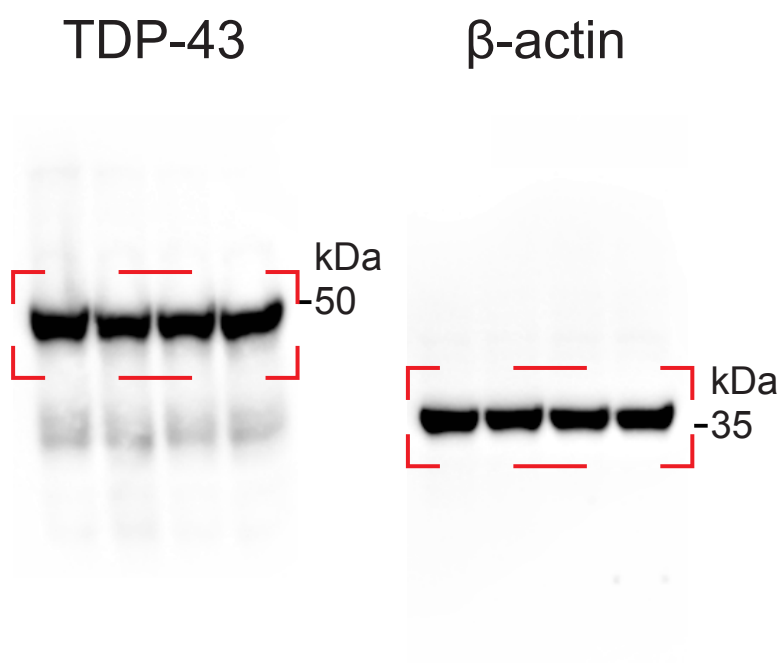

Fig. 5c

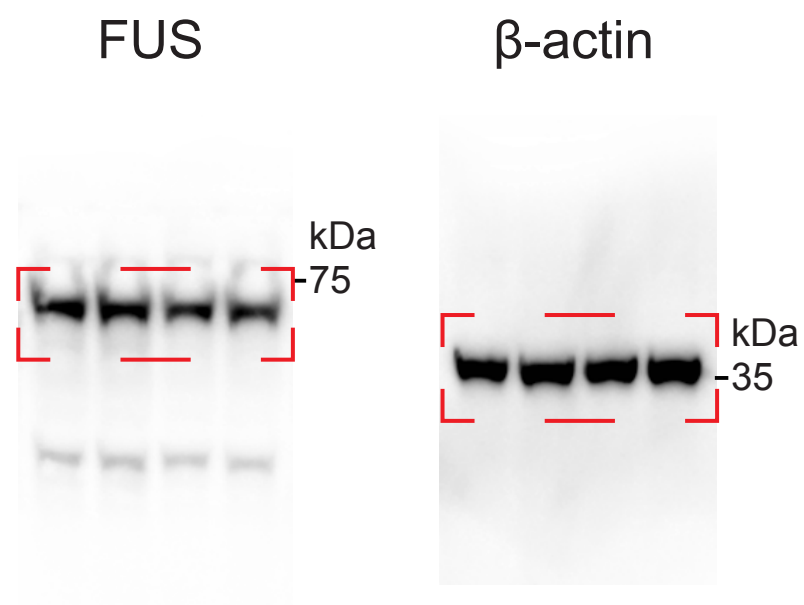

Fig. 5d

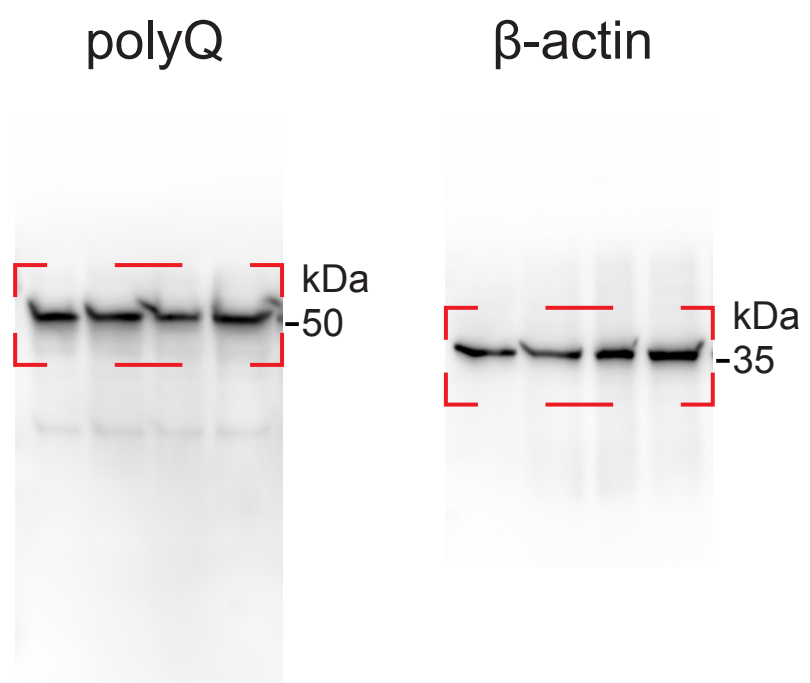

Fig. 5g

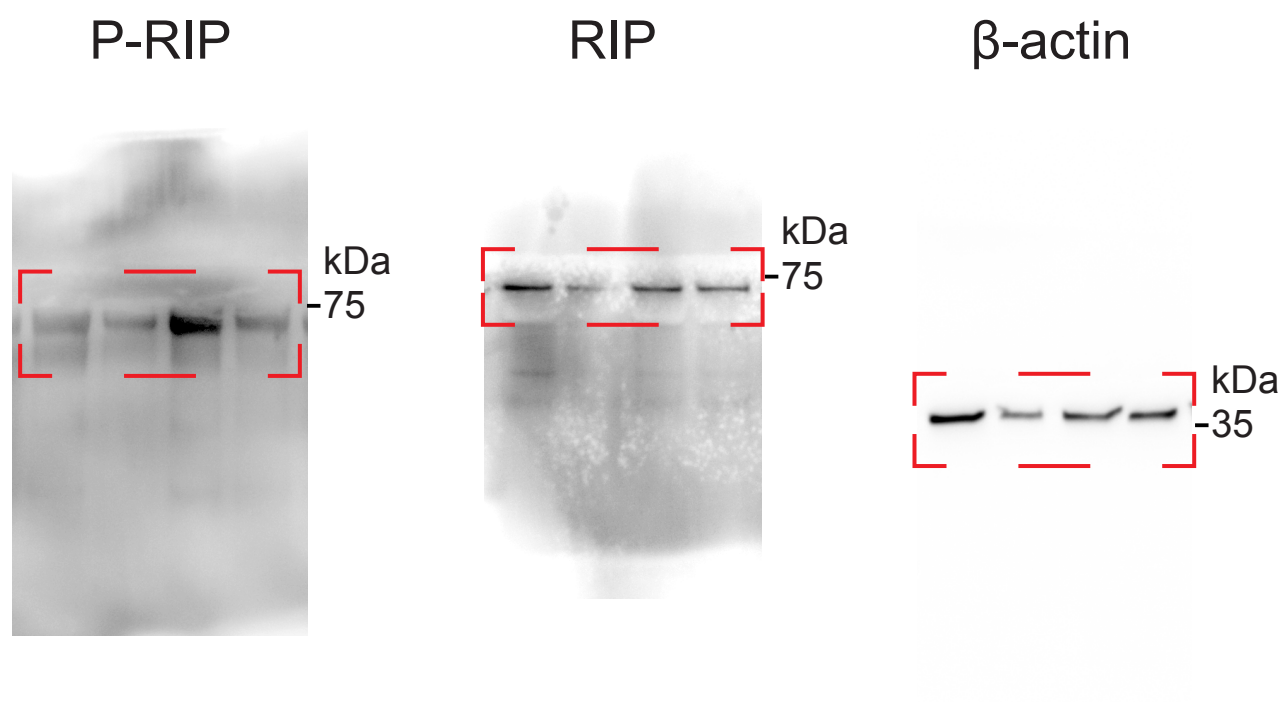

Extended Data Fig. 2g

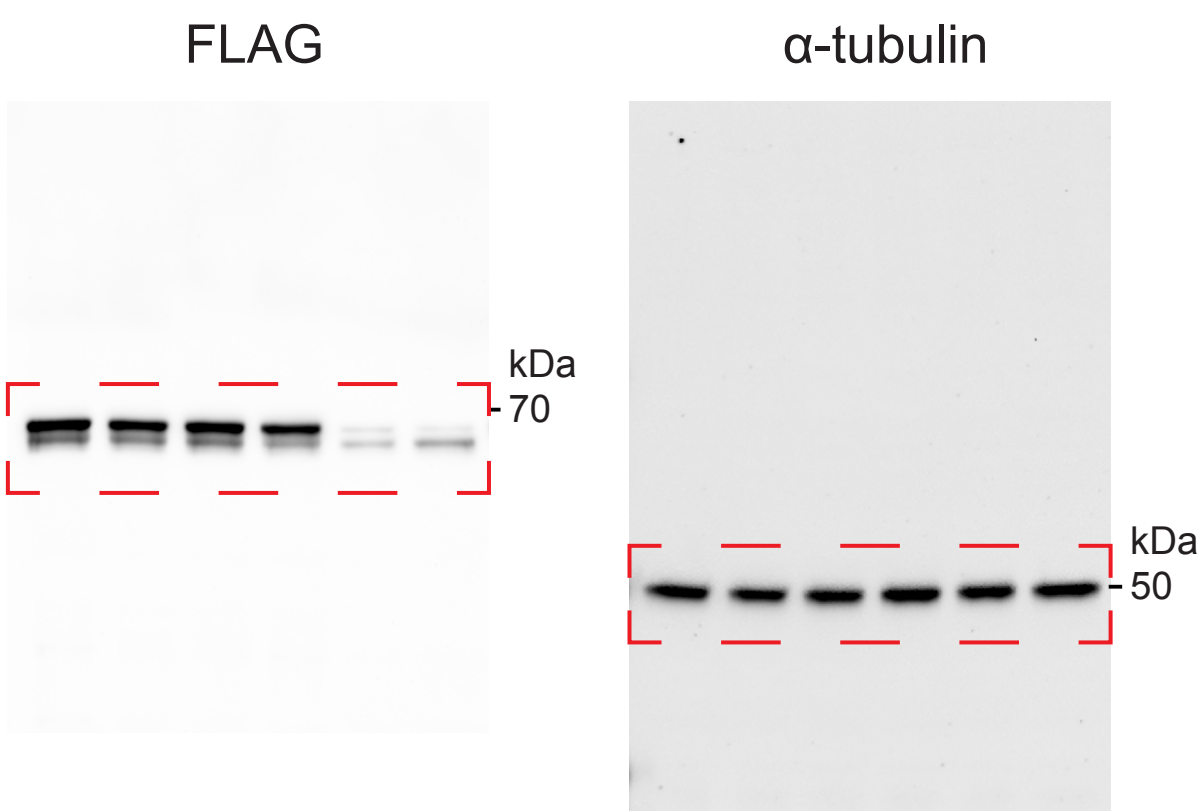

Extended Data Fig. 6b

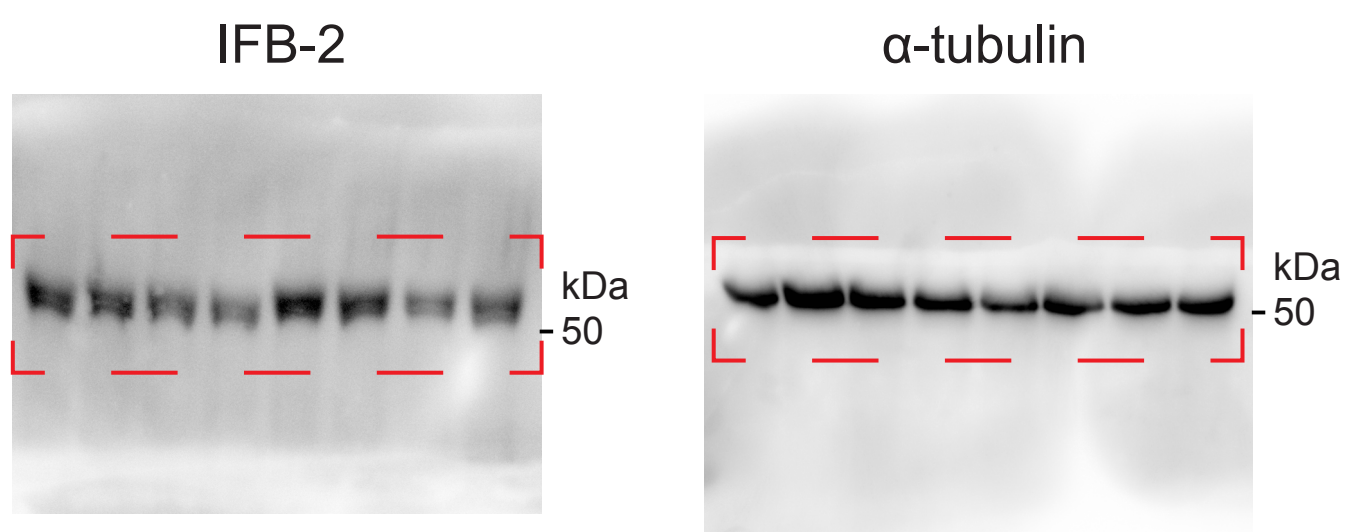

Extended Data Fig. 10c

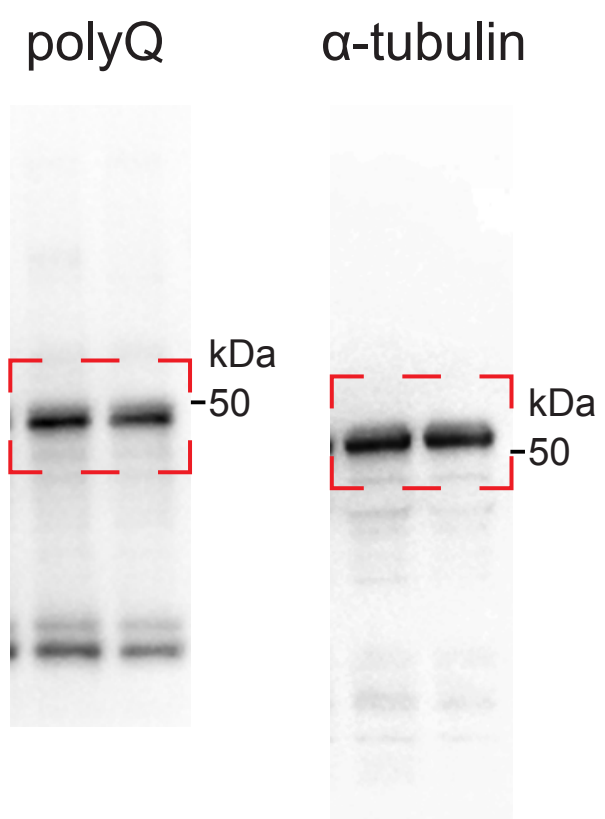

Extended Data Fig. 10f

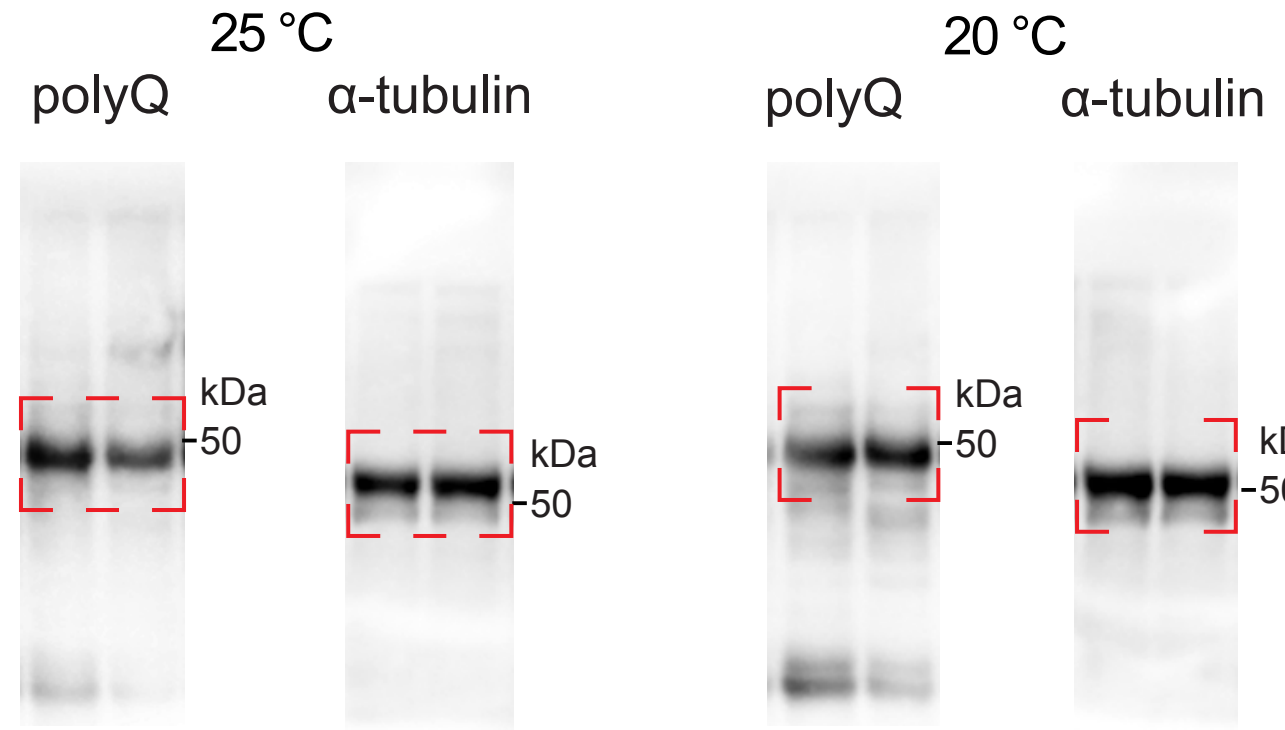

Extended Data Fig. 11a

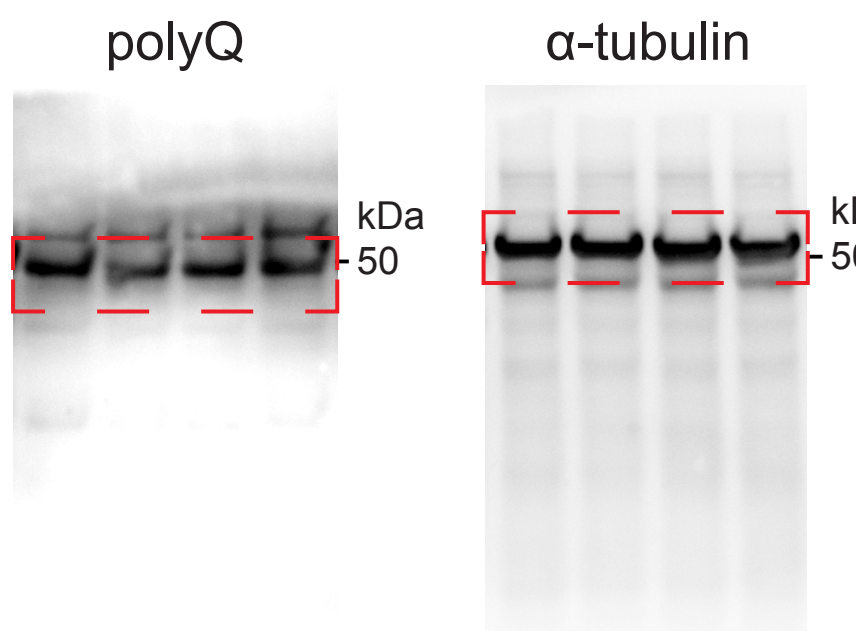

Extended Data Fig. 11b

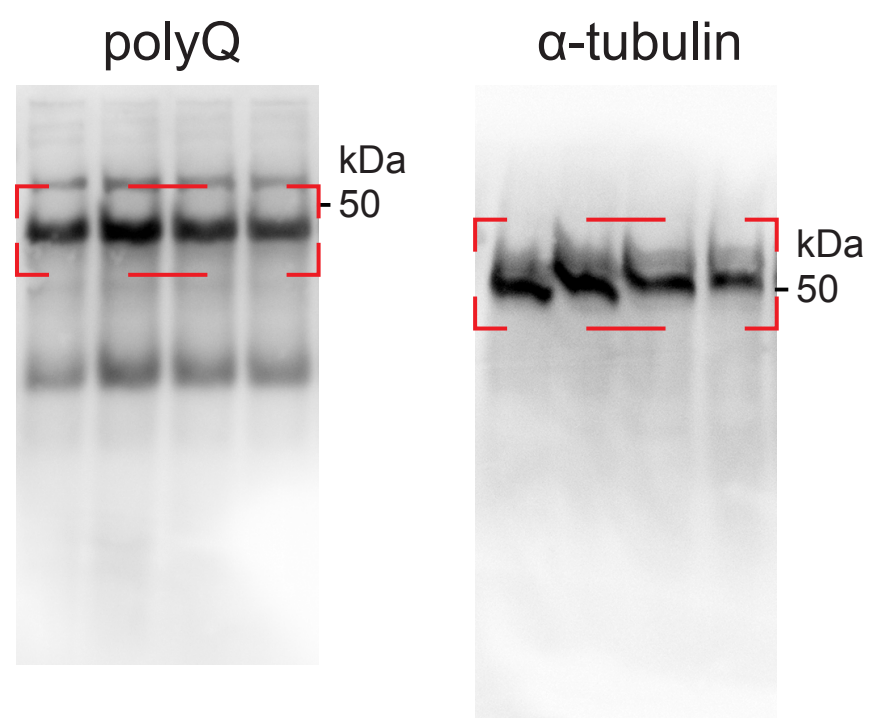

Extended Data Fig. 11c

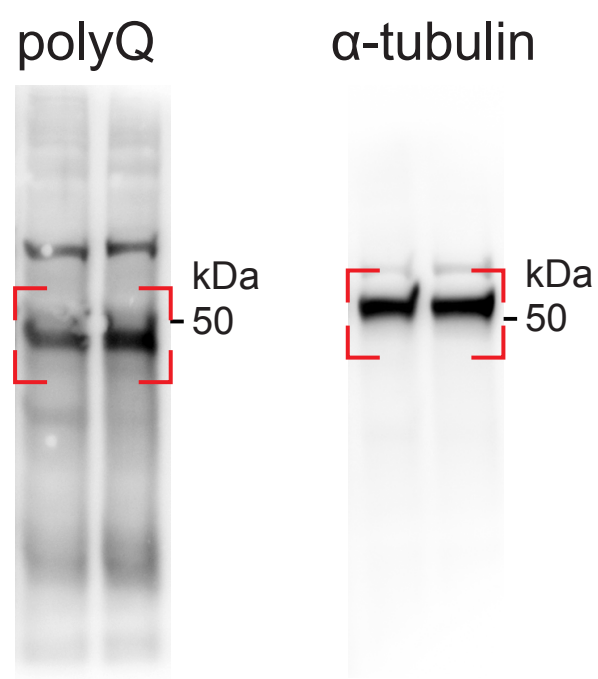

Extended Data Fig. 11d

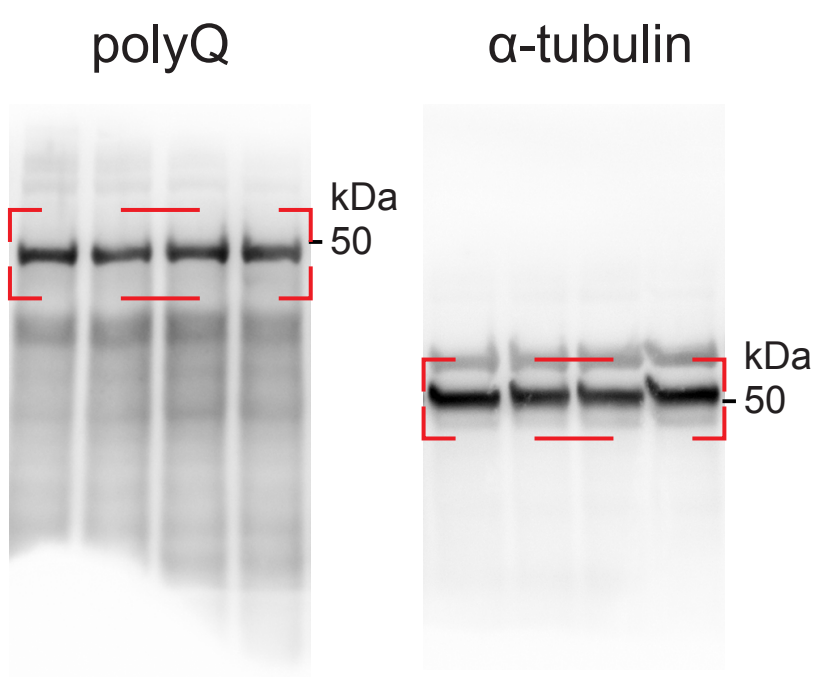

Extended Data Fig. 12c

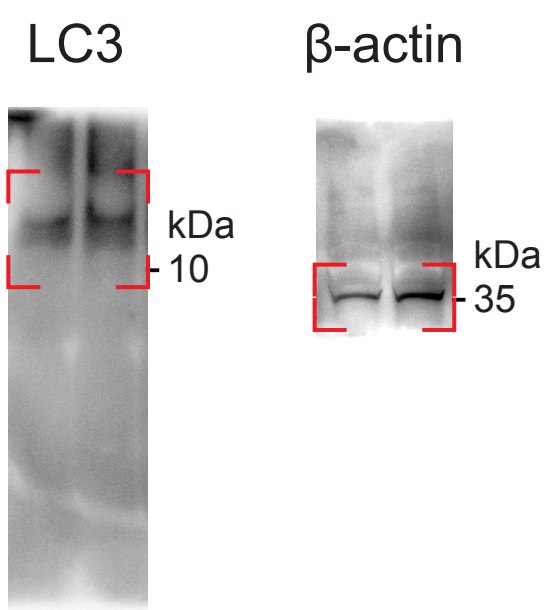

Extended Data Fig. 12d

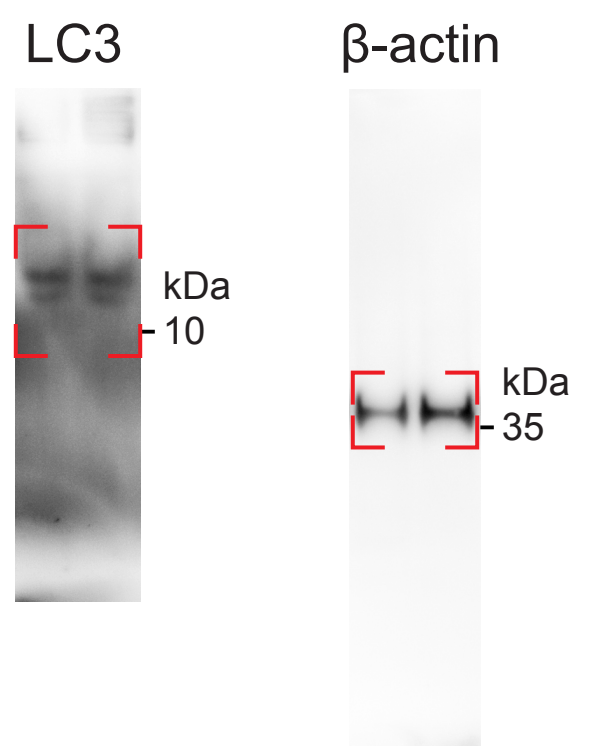

Extended Data Fig. 12e

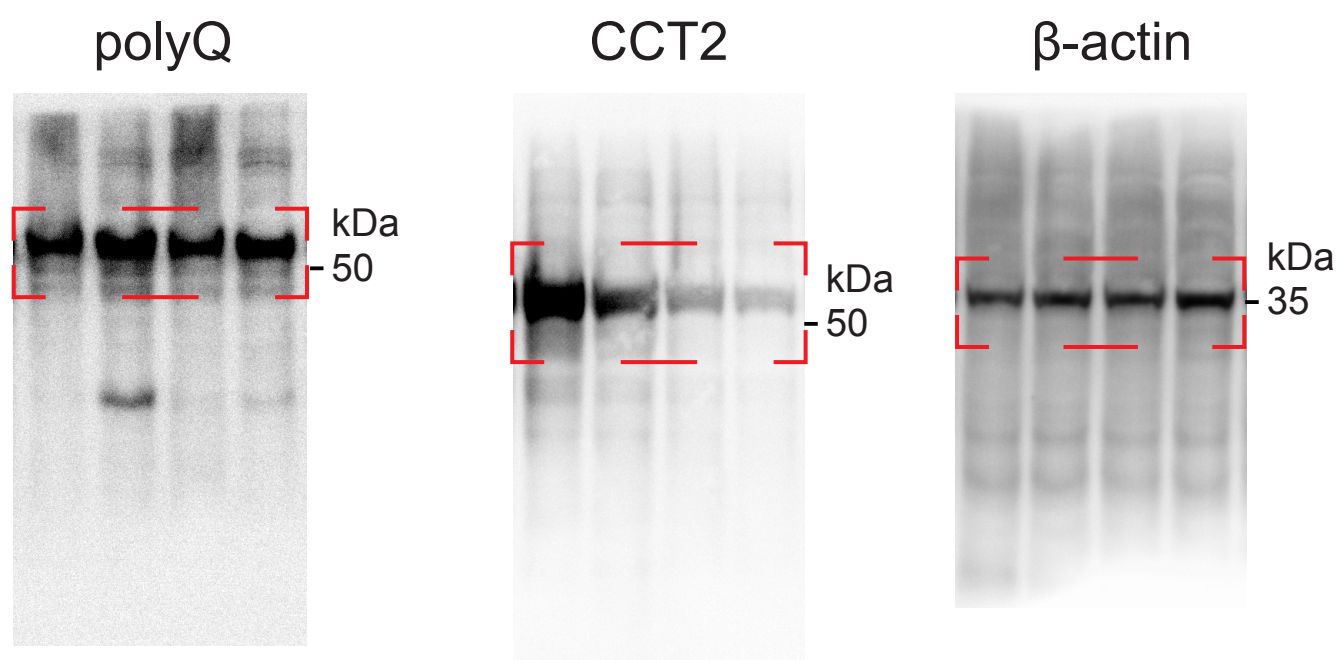

Extended Data Fig. 12g

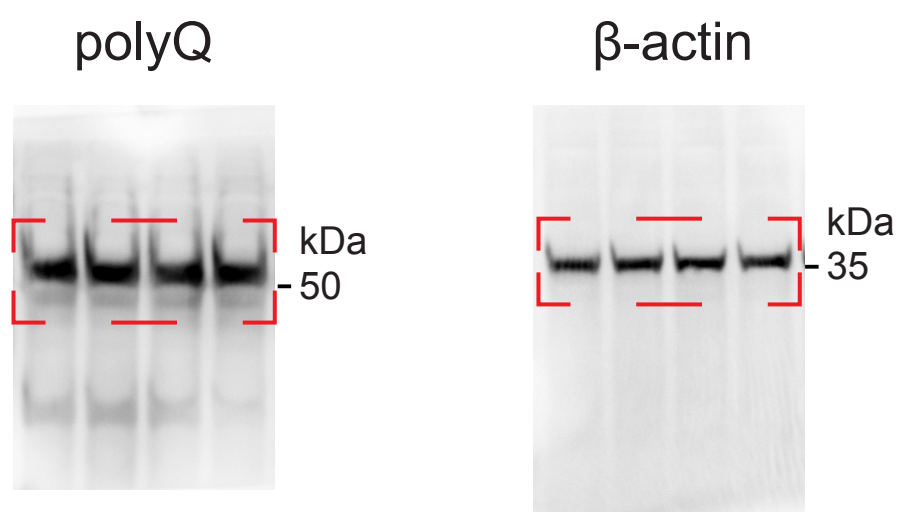

Extended Data Fig. 12h

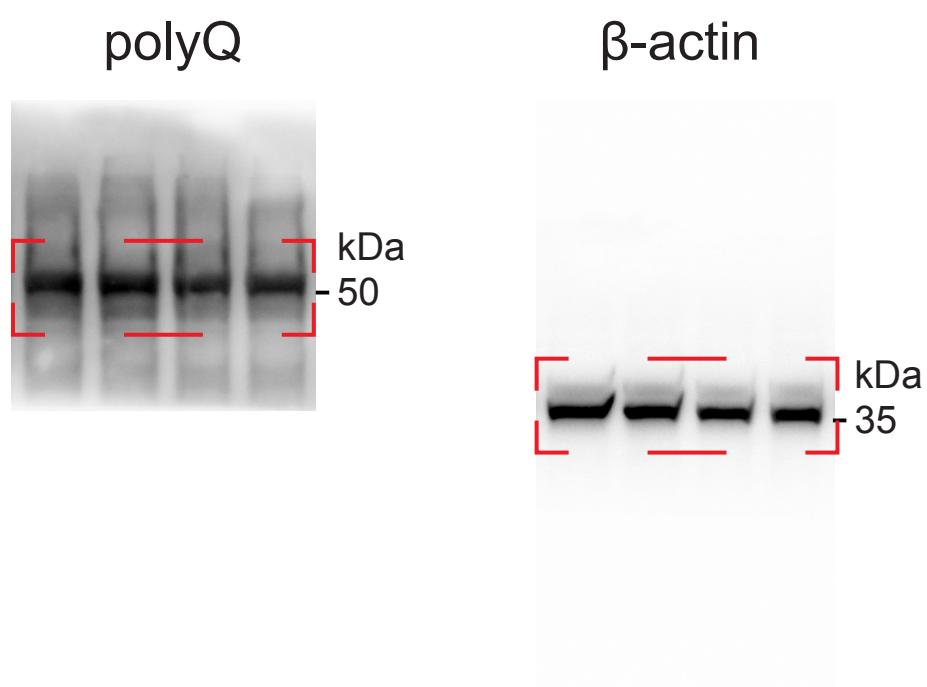

Extended Data Fig. 12i

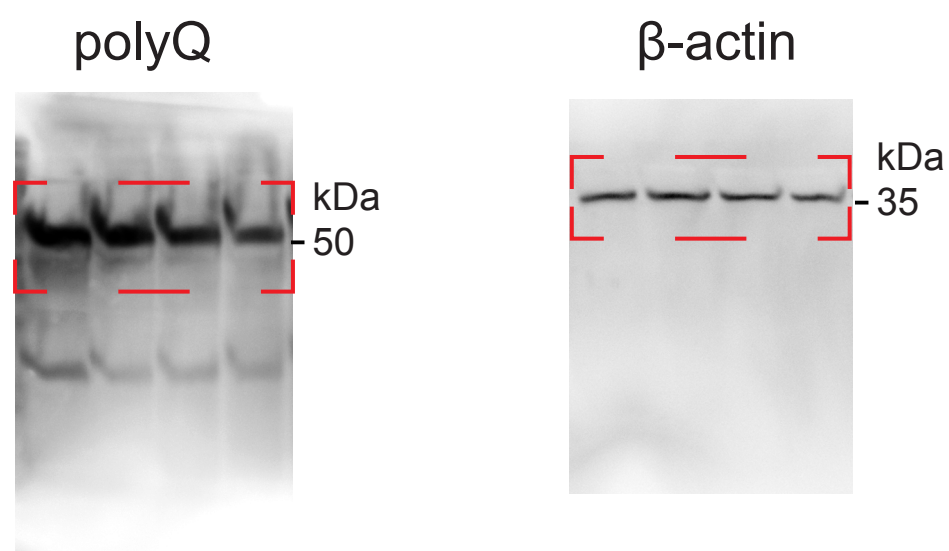

Supplement: Supplementary file 2 [file 41586_2026_11152_MOESM1_ESM.pdf]
