# Supplementary material for: Cold activates a BH2 metabolic network promoting longevity and proteostasis
Source: Nature. 2026 Oct 21;659(8140):480–9. doi: 10.1038/s41586-026-11152-9 (PMC13754623; doi:10.1038/s41586-026-11152-9)
Supplement: Supplementary file 3 [file 41586_2026_11152_MOESM2_ESM.pdf]

nature portfolio

Corresponding author(s): David Vilchez & Hyun Ju Lee

Last updated by author(s): Sep 4, 2026

Reporting Summary

Nature Portfolio wishes to improve the reproducibility of the work that we publish. This form provides structure for consistency and transparency in reporting. For further information on Nature Portfolio policies, see our Editorial Policies and the Editorial Policy Checklist.

Statistics

For all statistical analyses, confirm that the following items are present in the figure legend, table legend, main text, or Methods section.

n/a	Confirmed	
☐	☒	The exact sample size (*n*) for each experimental group/condition, given as a discrete number and unit of measurement
☐	☒	A statement on whether measurements were taken from distinct samples or whether the same sample was measured repeatedly
☐	☒	The statistical test(s) used AND whether they are one- or two-sided<br>*Only common tests should be described solely by name; describe more complex techniques in the Methods section.*
☒	☐	A description of all covariates tested
☐	☒	A description of any assumptions or corrections, such as tests of normality and adjustment for multiple comparisons
☐	☒	A full description of the statistical parameters including central tendency (e.g. means) or other basic estimates (e.g. regression coefficient) AND variation (e.g. standard deviation) or associated estimates of uncertainty (e.g. confidence intervals)
☐	☒	For null hypothesis testing, the test statistic (e.g. *F*, *t*, *r*) with confidence intervals, effect sizes, degrees of freedom and *P* value noted<br>*Give P values as exact values whenever suitable.*
☒	☐	For Bayesian analysis, information on the choice of priors and Markov chain Monte Carlo settings
☒	☐	For hierarchical and complex designs, identification of the appropriate level for tests and full reporting of outcomes
☐	☒	Estimates of effect sizes (e.g. Cohen's *d*, Pearson's *r*), indicating how they were calculated

Our web collection on statistics for biologists contains articles on many of the points above.

Software and code

Policy information about availability of computer code

Data collection	No software was used for data collection.
Data analysis	We used GraphPad Prism (version 10.6.0) for statistical analysis of all the data. For lifespan experiments, we used GraphPad Prism (version 10.6.0) and OASIS (version 2: Han et al. OASIS 2: online application for survival analysis 2 with features for the analysis of maximal lifespan and healthspan in aging research. Oncotarget 7:56147-56152 (2016)) to determine median and mean lifespan, respectively.<br>Densitometry of filter trap and western blotting assays was quantified using ImageJ software (version 1.51s). For quantification of GFP fluorescence signal in cat--4 reporter C. elegans strain, the anterior region of the intestine was outlined and quantified using ImageJ (version 1.54p). For quantification of bacterial colonization (mCherry signal) , worms were outlined and quantified using ImageJ software (version 1.51s).<br>For temperature-comparison proteomics datasets, samples were analyzed using DIA-NN 1.8.1 (Demichev, V., Messner, C. B., Vernardis, S. I., Lilley, K. S. & Ralser, M. DIA-NN: neural networks and interference correction enable deep proteome coverage in high throughput. Nat Methods 17, 41-44 (2020)) and the C. elegans proteomic database from WormBase (PRJNA13758.WBPS15, downloaded on 27/07/2021). LFQ values were calculated using the DIA-NN R-package (code available in: https://github.com/vdemichev/Diann-repackage). Afterwards, analysis of results was performed in Perseus (version 1.6.15) by filtering for data completeness in at least one replicate group and missing values imputed using sigma downshift approach with standard settings. Afterwards, FDR-controlled t-tests were performed between the experimental groups and WormBase IDs mapped to UniProt IDs. For tyrosine-treatment proteomics datasets, a directDIA approach using Spectronaut 20.5 (Biognosys) was used with the GPF runs added as supporting runs and searched against the WormBase C. elegans proteomic database (PRJNA13758.WBPS19, downloaded 04/01/2025). The Spectronaut output was imported into R (version 4.5.2) for downstream analysis. Protein groups were expanded to individual UniProt identifiers, mapped to gene symbols using the C. elegans annotation database (org.Ce.eg.db, version 3.22.0), and protein abundances were summarized at the gene level using median values. Proteins with > 50% missing values across samples were excluded. Protein intensities were log2-transformed following addition of a pseudocount of 1. Differential protein

abundance analysis was conducted using the limma package (version 3.66.0) with empirical Bayes moderation.
Over-representation analysis (ORA) was performed using the enrichGO function from the clusterProfiler package in R (version 4.5.2) to identify Gene Ontology (GO) terms enriched among differentially abundant proteins. Analyses were conducted using the org.Ce.eg.db annotation database (version 3.22.0). GO Biological Process (BP) and Molecular Function (MF) ontologies were analysed using a one-sided hypergeometric test. Multiple-testing correction was performed using the Benjamini–Hochberg method, with significance thresholds of $P < 0.05$ and $q < 0.2$. For visualization, enrichment results were converted to gene ratios (GeneRatio), representing the proportion of input genes associated with each GO term. Dot plots were generated using ggplot2 (version 4.0.3), with the x axis representing the gene ratio, the y axis indicating GO terms, point size corresponding to the number of genes associated with each term (Count), and point color representing statistical significance (−log10 P value).
For metabolomics experiments, metabolite identification was performed using Compound Discoverer software (version 3.2, Thermo Fisher Scientific), with criteria including a precursor ion m/z within 5�ppm of the theoretical mass, fragment ions within 5 ppm of an internal spectral library, a minimum match score of 70, and retention times within 5% of purified standards analyzed under identical conditions. Peak area integration and chromatogram review were carried out using TraceFinder software (version 5.0, Thermo Fisher Scientific). To correct any potential variations introduced from sample handling to instrument analysis, we normalized the detected metabolites in each sample using the probabilistic quotient normalization (PQN) method. The data were processed using the R package MetaProViz54 (https://github.com/saezlab/MetaProViz), with the only modification being that PQN normalization was performed using 100% of the features from multtest package (version 2.56.0), instead of total ion count (TIC) normalization. Downstream statistical analyses were performed using GraphPad Prism (version 10.6.0). Data were analyzed with two-way unpaired t-tests, followed by the two-stage step-up method of Benjamini, Krieger, and Yekutieli to correct for multiple comparisons. For correlation analysis of metabolite changes between independent datasets (experiments 1 and 2), metabolites were filtered to retain Metabolites, log2 fold change, and FDR-adjusted P-value (q value). The datasets were merged using an inner join on metabolite names. Significance was defined as an FDR-adjusted P-value (q value) < 0.01. Pearson correlation was calculated using only metabolites that were significant in both datasets. Scatter plots showing log2 fold changes from experiments 1 and 2 were generated using the ggplot2 package (version 4.0.3) in R (v4.5.2). Reference lines at zero (x and y axes) and a diagonal identity line (slope=1) were included to aid interpretation. The Pearson correlation coefficient (r) and corresponding P value were annotated on the plot. Extreme outliers (for example, carnitine-C16) were excluded prior to visualization. For Venn diagram analysis, significantly upregulated metabolites were identified independently in each dataset using thresholds of fold change >1 and FDR (q value)<0.01. Venn diagrams were generated using the ggvenn package (version 0.1.19) in R (version 4.5.2). Dot plots comparing the 45 commonly and significantly upregulated metabolites (fold change >1 and q value <0.01) across experiments 1 and 2 were generated using ggplot2 (version 4.0.3) in R (version 4.5.2).

For manuscripts utilizing custom algorithms or software that are central to the research but not yet described in published literature, software must be made available to editors and reviewers. We strongly encourage code deposition in a community repository (e.g. GitHub). See the Nature Portfolio guidelines for submitting code & software for further information.

Data

Policy information about availability of data

All manuscripts must include a data availability statement. This statement should provide the following information, where applicable:

- Accession codes, unique identifiers, or web links for publicly available datasets
- A description of any restrictions on data availability
- For clinical datasets or third party data, please ensure that the statement adheres to our policy

All data supporting the findings of this study are available within the paper and its Supplementary Information files. Proteomics data have been deposited in the ProteomeXchange Consortium via the PRIDE partner repository under dataset identifiers PXD059883 (C. elegans at different temperatures) and PXD079702 (tyrosine supplementation in C. elegans). Metabolomics data have been deposited in the NIH Common Fund's National Metabolomics Data Repository (NMDR), the Metabolomics Workbench (https://www.metabolomicsworkbench.org)82, under Study IDs ST004426 (cold-induced metabolites in day-5 adult worms, Experiment 1), ST004433 (cold-induced metabolites in day-5 adult worms, Experiment 2), ST004434 (cold-induced metabolites in day-1, 10 and 15 adult worms), and ST004437 (metabolomics analysis in wild-type and long-lived daf-2 mutant C. elegans at different ages). These data can be accessed directly via the project DOI: http://dx.doi.org/10.21228/M84530. The whole-genome expression profiling of C. elegans adults was obtained from the NCBI GEO database under accession number GSE6229714 (https://www.ncbi.nlm.nih.gov/geo/query/acc.cgi?acc=GSE62297). The C. elegans annotation database (org.Ce.eg.db, version 3.22.0) was obtained from Bioconductor (https://bioconductor.org/packages/release/data/annotation/html/org.Ce.eg.db.html). Source data are provided with this paper.

Research involving human participants, their data, or biological material

Policy information about studies with human participants or human data. See also policy information about sex, gender (identity/presentation), and sexual orientation and race, ethnicity and racism.

Reporting on sex and gender	N/A
Reporting on race, ethnicity, or other socially relevant groupings	N/A
Population characteristics	N/A
Recruitment	N/A
Ethics oversight	N/A

Note that full information on the approval of the study protocol must also be provided in the manuscript.

Field-specific reporting

Please select the one below that is the best fit for your research. If you are not sure, read the appropriate sections before making your selection.

☒ Life sciences ☐ Behavioural & social sciences ☐ Ecological, evolutionary & environmental sciences

For a reference copy of the document with all sections, see nature.com/documents/nr-reporting-summary-flat.pdf

Life sciences study design

All studies must disclose on these points even when the disclosure is negative.

Sample size

Exact sample sizes are indicated in the corresponding figure legends.
No statistical methods were used to predetermine sample size. However, our sample sizes were selected based on standards established in the field and are similar to, or greater than, those reported to be sufficient in previous publications using the same procedures (i.e. lifespan, nose-touch, chemotaxis, motility, percentage of activated caspase-3 in human neurons, filter trap assays, western blotting, label-free proteomics):

- Llamas et al, Aging Cell 20: e13446
- Lee HL et al, Nature Metabolism 1: 790-810 (2019)
- Lee HL et al, Nature Aging 3:546-566 (2023)
- Koyuncu S et al, Nature Communications 9: 2886 (2018)
- Amrit FR et al, Methods 68: 465–475 (2014)
- Fatima A et al, Communications Biology 3: 262
- Koyuncu S et al, Nature 596:285-290 (2021)
- Alirzayeva H et al, Cell Reports 43: 114626 (2024)
- Koyuncu S et al, Nature Aging 5:1750–1770 (2025)
- Segref A et al, Nature Communications 13: 5874 (2022))
- Koopman M et al, MicroPublication Biology: 10.17912/micropub.biology.000769 (2023)
- Fernandez-Abascal J, Neuron 110:470–485 (2022)
- Vilchez D et al, Nature 489:263-8 (2012)
- Vilchez D et al, Nature 489:304-8 (2012)

Data exclusions

No data were excluded from the analyses.

Replication

At least three independent experiments were conducted for each assay to verify the reproducibility of the findings. If only two independent experiments were performed, this is indicated in the figure legend. All replication attempts yielded similar results. Lifespan assays were conducted at least twice, with at least 96 animals per condition. Exact sample sizes and the number of independent experiments are specified in the corresponding figure legends.

Randomization

For C. elegans experiments, worm populations were synchronized either by allowing young hermaphrodites to lay eggs for 6h or by using the bleaching method (Porta-de-la-Riva M, Fontrodona L, Villanueva A, Cerón JC. Basic Caenorhabditis elegans methods: synchronization and observation. J Vis Exp. 2012 Jun 10:(64). doi: 10.3791/4019.). For synchronization by egg laying, young hermaphrodites were randomly selected from maintenance plates. For synchronization by bleaching, random agar chunks containing mixed-stage animals were transferred from maintenance plates, and larvae were allowed to develop until a sufficient number of young hermaphrodites were available for bleaching. After the 6-h egg-laying period or bleaching, the resulting larvae were raised to adulthood. For lifespan assays, adult worms were randomly picked from the synchronized population and allocated to the different experimental conditions. For all other experiments, adult worms were randomly distributed from single synchronized populations into the different experimental groups. Thus, allocation of worms to experimental conditions was randomized in all the experiments. Samples from the different experimental conditions were collected and lysed in a randomized order to minimize potential processing-order effects.

For human cell line experiments, cells at similar confluence were split, and equal numbers of cells were transferred to new plates. Plates were randomly assigned to the different treatment conditions, and samples were collected and lysed in a randomized order to minimize potential processing-order effects.

Blinding

The samples and experimental conditions were not processed in a blinded manner by the researchers involved in this study. However, cells and worms were randomly allocated from single pulls to different treatment conditions, and key experiments were independently repeated by at least two of the researchers involved in this study to ensure reproducibility. For experiments with direct phenotypic outputs—such as lifespan assays, nose-touch assays, chemotaxis assays, and activated caspase-3 levels in human neurons—blinding was not applied during data collection or analysis. This was due to the pronounced and well-characterized phenotypes associated with the different temperatures and disease models used in this study. Nonetheless, worms were randomly assigned to treatment groups, and experimental conditions were assessed in a randomized order. These assays and their analyses were also independently repeated by different researchers to confirm robustness and reproducibility. For proteomics and metabolomics experiments, the mass spectrometry was performed by the CECAD Proteomics Facility and Metabolomics Facility in a blinded manner. Filter trap and western blot assays were not performed under blinded conditions, as these rely on objective instrument-based measurements and/or provide indirect quantitative outputs. Data analysis for these assays was unblinded, as the investigators who performed the analysis also loaded the samples during the experiment, and the corresponding outputs from the measurement equipment were released in sequence.

Reporting for specific materials, systems and methods

We require information from authors about some types of materials, experimental systems and methods used in many studies. Here, indicate whether each material, system or method listed is relevant to your study. If you are not sure if a list item applies to your research, read the appropriate section before selecting a response.

Materials & experimental systems

n/a	Involved in the study
☐	☒ Antibodies
☐	☒ Eukaryotic cell lines
☒	☐ Palaeontology and archaeology
☐	☒ Animals and other organisms
☒	☐ Clinical data
☒	☐ Dual use research of concern
☒	☐ Plants

Methods

n/a	Involved in the study
☒	☐ ChIP-seq
☒	☐ Flow cytometry
☒	☐ MRI-based neuroimaging

Antibodies

Antibodies used

We used the following antibodies in this study:

*For western blot:
anti-α-tubulin (Sigma, T6199, 1:5,000. RRID: AB_477583). Monoclonal, clone number: DM1A
anti-β-actin (Abcam, ab8226, 1:1,000. RRID: AB_306371). Monoclonal, clone number: mAbcam 8226
anti-GFP (AMSBIO, 210-PS-1GFP, 1:5,000. RRID: AB_10013682). Polyclonal.
anti-mCherry (Abcam, ab167453, 1:5,000). Polyclonal
anti-FUS (Abcam, ab154141, 1:1000. RRID: AB_2885092). Monoclonal, clone number: CL0190.
anti-TDP43 (Abcam, ab225710, 1:1000). Polyclonal.
anti-IFB-2 (Developmental Studies Hybridoma Bank, MH33, 1:1,000. RRID: AB_528311)
anti-Phospho-RIP (Ser166) (Cell Signaling, #65746, 1:1,000). Monoclonal, clone number: D1L3S
anti-RIP (Cell Signaling, #3493, 1:1,000). Monoclonal, clone number: D94C12
anti-LC3B (Sigma, L7543, 1:1,000. RRID: AB_796155)
anti-CCT2 (Abcam, ab92746, 1:1,000)
anti-FLAG M2-HRP mouse (Sigma-Aldrich, A8592, 1:10,000, RRID:AB_439702). Monoclonal, clone number: M2
anti-α-tubulin mouse (Sigma-Aldrich, T6074, 1:5,000, RRID:AB_477582). Monclonal, clone number: B-5-1-2
IRDye 800CW secondary antibody (Li-Cor, 926-322101:10,000)

*Filter trap assay:
anti-GFP (AMSBIO, 210-PS-1GFP, 1:5,000. RRID: AB_10013682). Polyclonal.
anti-mCherry (Abcam, ab167453, 1:5,000). Polyclonal
anti-FUS (Abcam, ab154141, 1:1000. RRID: AB_2885092). Monoclonal, clone number: CL0190.
anti-TDP43 (Abcam, ab225710, 1:1000). Polyclonal.
anti-IFB-2 (Developmental Studies Hybridoma Bank, MH33, 1:1,000. RRID: AB_528311)

*Immunocytochemistry:
anti-Cleaved Caspase 3 (Cell Signaling, #9661S, 1:300. RRID: AB_2341188). Polyclonal.
anti-MAP2 (2a+2b) (Sigma-Aldrich, #M1406, 1:300. RRID: AB_477171). Monoclonal, clone number: AP-20
Alexa Fluor 488 Goat anti-Mouse IgG (H+L) (ThermoFisher, A-11029, 1:500. RRID: AB_2534088). Polyclonal.
Alexa Fluor 568F(ab')2 Fragment of Goat Anti-Rabbit IgG (H+L) (ThermoFisher, A-21069, 1:500. RRID: AB_141416). Polyclonal.

Validation

Validation of antibodies were done by the stated manufacturer's, this study, or previous publications and supported by the publications indicated in the manufacturer's website, the Resource Identification Portal (RRID) and other publications using C. elegans and human cells.
* anti-α-tubulin (Sigma, T6199, 1:5,000. RRID: AB_477583). The antibody was validated as a loading control for western blot analysis in C. elegans in our previous publications: PMID: 32451438; PMID: 27892468; PMID: 34172445; PMID: 34321666; PMID: 37118550; PMID: 40903652.
*anti-β-actin (Abcam, ab8226, 1:1,000. RRID: AB_306371) was used according to the manufacturer's instructions and our previous publications: PMID: 27892468; PMID: 30038412; PMID: 32451438; PMID: 37118550; PMID: 40903652.
* anti-GFP (AMSBIO, 210-PS-1GFP, 1:5,000. RRID: AB_10013682). This antibody has been validated for filter trap and western blot in C. elegans and human cells in our previous publications: PMID: 27892468; PMID: 30038412; PMID: 34172445; PMID: 34321666; PMID: 37118550; PMID: 40903652.
* anti-mCherry (Abcam, ab167453, 1:5,000). This antibody has been validated for filter trap and western blot in C. elegans and human cells in our previous publications: PMID: 37783816.
* anti-FUS (Abcam, ab154141, 1:1000. RRID:AB_2885092) was used according to the manufacturer's instructions and our previous publications for filter trap and western blot experiments: PMID: 30038412; PMID: 34172445; PMID: 37118550; PMID: 40903652.

* anti-TDP43 (Abcam, ab225710, 1:1000) was used according to the manufacturer's instructions and our previous publications for filter trap and western blot experiments where it was previously validated: PMID: 34172445; PMID: 37118550; PMID: 40903652.
* anti-Phospho-RIP (Ser166) (Cell Signaling, #65746, 1:1,000). The antibody was used according to the manufacturer's instructions, validated in previous studies (PMID: 39505876, PMID: 39526730, PMID: 40903652).
* anti-IFB-2 (Developmental Studies Hybridoma Bank, MH33, 1:1,000. RRID: AB_528311). The antibody was used according to the manufacturer's instructions for C. elegans (References: PMID:31414984, PMID:31414984) and our previous publication (western blot, filter trap): Koyuncu S et al, Nature 596:285-290 (2021), PMID: 34321666.
* anti-FLAG M2-HRP mouse (Sigma-Aldrich, A8592, 1:10,000, RRID:AB_439702). This antibody has been used in multiple studies: PMID:41503209, PMID:40849907, etc
* anti-α-tubulin mouse (Sigma-Aldrich, T6074, 1:5,000, RRID:AB_477582). This antibody has been used in multiple studies: PMID:40037356, PMID:41509924, etc
* anti-Phospho-RIP (Ser166) (Cell Signaling, #65746, 1:1,000). The antibody was used according to the manufacturer's instructions, validated in previous studies (PMID: 39505876, PMID: 39526730).
* anti-RIP (Cell Signaling, #3493, 1:1,000). The antibody was used according to the manufacturer's instructions, validated in previous studies (PMID: 39681571, PMID: 39753884,;PMID: 40903652).
* anti-Cleaved Caspase 3 (Cell Signaling, #9661S, 1:300. RRID:AB_2341188) was used according to the manufacturer's instructions and validated in multiple studies and our previous publication (e.g. PMID:16736467, PMID:17099894, PMID:17299760, PMID:17990272, PMID:19830812, PMID:20235094, PMID:20593360, PMID:20653033, PMID:20653035, PMID: 37118550, PMID: 40903652).
* anti-MAP2 (2a+2b) (Sigma-Aldrich, #M1406, 1:300. RRID:AB_477171) was used according to the manufacturer's instructions and validated in multiple studies and our previous publication (e.g. PMID:19058188, PMID:19950118, PMID:26509469, PMID: 37118550 etc).
* anti-LC3B (Sigma, L7543, 1:1,000. RRID: AB_796155). Validated in previous publications (e.g. PMID: 30038412)
* anti-CCT2 (Abcam, ab92746, 1:1,000). Validated in previous publications (e.g. PMID: 27892468)
*Alexa Fluor 488 Goat anti-Mouse IgG (H+L) (ThermoFisher, A-11029, 1:500. RRID:AB_2534088) was used according to the manufacturer's instructions and validated in multiple studies (e.g. PMID:34995520, PMID:35194846, PMID:35219381).
*Alexa Fluor 568F(ab')2 Fragment of Goat Anti-Rabbit IgG (H+L) (ThermoFisher, A-21069, 1:500. RRID:AB_141416) was used according to the manufacturer's instructions and validated in multiple studies (e.g. PMID:35111373, PMID:31526765, PMID:29103933).

Eukaryotic cell lines

Policy information about cell lines and Sex and Gender in Research

Cell line source(s)

In this study, we used the human HEK293 cell line (HEK293T/17) obtained from the American Type Culture Collection (ATCC). Catalog number: CRL-11268.
ALS-iPSCs (FUSP525L/P525L) and their isogenic controls were kindly provided by I. Bozzoni and A. Rosa (Sapienza University of Rome). These iPSC lines were established and characterized for pluripotency in ref.: Lenzi J et al. ALS mutant FUS proteins are recruited into stress granules in induced pluripotent stem cell-derived motoneurons. Dis Model Mech 8: 755-766 (2015), (PMID: 26035390). ALS-iPSCs were raised from the control iPSCs by TALEN (transcription activator-like effector nucleases)-directed mutagenesis and are homozygote for a FUS mutation (P525L) linked with severe ALS (Lenzi J et al. Dis Model Mech 8: 755-766 (2015)).

Authentication

The HEK293T/17 cell line was commercially obtained from ATTC and has not been authenticated in our laboratory. The identity of both iPSC lines was authenticated in our laboratory by short tandem repeat (STR) profiling using the microsatellite markers D17S1303, D16S539, vWA, THO1, CSF1PO and TPOX in a previous publication (PMID: 30038412). We confirmed that the STR profile of the ALS-iPSCs matches with the profile of their isogenic control iPSCs (PMID: 30038412). The iPSC lines used in the present study were thawed directly from frozen stocks of these authenticated lines.

Mycoplasma contamination

All the cell lines used in this study were tested for mycoplasma contamination at least once every 3 weeks. No mycoplasma contamination was detected.

Commonly misidentified lines
(See ICLAC register)

None of the cell lines used in this paper are listed in the database of commonly misidentified cell lines maintained by ICLAC (version 14, released 15th February 2025)

Animals and other research organisms

Policy information about studies involving animals; ARRIVE guidelines recommended for reporting animal research, and Sex and Gender in Research

Laboratory animals

In this study, we used different Caenorhabditis elegans strains. For all the experiments, we used hermaphrodites worms.

Lifespan analysis was started from day 1 of adulthood. For all the other experiments on C. elegans, the specific age is indicated in the corresponding figures and/or figure legends.

The C. elegans strains used in this study were:

Wild-type (N2)
TQ233 (trpa-1(ok999)IV)
AM141 (rmIs133[unc-54p::Q40::yellow fluorescent protein (YFP)])
AM23 (rmIs298[F25B3.3p::Q19::CFP])
AM716 (rmIs284[F25B3.3p::Q67::YFP])

MAH602 (sqIs61[vha-6p::Q44::YFP + rol-6(su1006)])
CF1041 (daf-2(e1370)III)
CF512 (fer-15(b26)II;fem-1(hc17)IV)
LC141 (him-8(e1489)IV;cat-4(e3015)V)
LC35 (cat-4(ok342)V)
LC81 (cat-4(tm773)V)
LC143 (pcbd-1(tm5924)I)
LC87 (qdpr-1(tm2337)III)
LC74 (pah-1(ok687)II)
MT15620 (cat-2(n4547)II)
MT14984 (tph-1(n4622)II)
LC144 (agmo-1(e3016))
MQ887 (isp-1(qm150)IV)
DA1116 (eat-2(ad1116)II)
DA597 (phm-2(ad597)I)
BJ49 (kcIs6[ifb-2p::ifb-2a::CFP]IV)
SJ4100 (zcIs13[hsp-6p::GFP])V, outcrossed 3x to N2)
DCL569 (mkcSi13[sun-1p::rde-1::sun-1 3'UTR + unc-119(+)]II; rde-1(mkc36)V)
VP303 (rde-1(ne219)V; kbIs7[nhx-2p::rde-1 + rol-6(su1006)]
WM118 (rde-1(ne300)V; neIs9[myo-3p::HA::RDE-1 + rol-6(su1006)]
NR222 (rde-1(ne219)V;kzIs9[lin-26p::NLS::GFP + lin-26p::rde-1 + rol-6(su1006)])
MAH677 (neuronal-specific RNAi, sid-1(qt9);sqIs71[rgef-1p::GFP + rgef-1p::sid-1]
DVG387 (cat-4::SL2::GFP)
VDL20/PHX10108 (cat-4(syb10108))
DVG9 (N2, ocbEx9[myo3p::GFP])
DVG348 (N2, ocbEx280[sur-5p::cat-4, myo-3p::GFP])
DVG349 (N2, ocbEx281[gly-19p::cat-4, myo-3p::GFP])
DVG351 (N2, ocbEx283[rgef-1p::cat-4, myo-3p::GFP])
DVG352 (N2, ocbEx284[myo-3p::cat-4, myo-3p::GFP])
DVG373 (rmIs298[F25B3.3p::Q19::CFP]; ocbEx297[myo-3p::cat-4, myo-3p::mCherry])
DVG375 (rmIs284[F25B3.3p::Q67::YFP], ocbEx299[myo-3p::cat-4, myo-3p::mCherry])
DVG376 (rmIs298[F25B3.3p::Q19::CFP]; ocbEx300[myo-3p::mCherry])
DVG380 (rmIs284[F25B3.3p::Q67::YFP], ocbEx304[myo-3p::mCherry])
DVG377 (sid-1(qt9);sqIs71[rgef-1p::GFP + rgef-1p::sid-1], ocbEx301[myo-3p::cat-4, myo-3p::mCherry])
DVG378 (sid-1(qt9);sqIs71[rgef-1p::GFP + rgef-1p::sid-1], ocbEx302[myo3p::mCherry])
DVG389 (trpa-1(ok999)IV;ocbEx309[myo-3p::GFP])
DVG390 (trpa-1(ok999)IV;ocbEx310[myo-3p::cat-4, myo-3p::GFP])
PP4642 (cat-4(hh131[cat-4::3xFLAG::mIAA7::gfp]V)
PP4640 (wrdSi58[eft-3p::TIR1(F79G)::F2A::mTagBFP2::AID*::NLS::tbb-2 3'UTR]II)
PP4641 (reSi11[unc-54p::TIR1(F79G)::F2A::mTagBFP2::AID*::NLS::tbb-2 3'UTR]II)
PP4634 (cat-4(hh131[cat-4::3xFLAG::mIAA7::GFP]V;wrdSi58[eft-3p::TIR1(F79G)::F2A::mTagBFP2::AID*::NLS::tbb-2 3'UTR]II)
PP4635 (cat-4(hh131[cat-4::3xFLAG::mIAA7::GFP]V; reSi11[unc-54p::TIR1(F79G)::F2A::mTagBFP2::AID*::NLS::tbb-2 3'UTR]II)
DVG397 (ent-1(ok743) IV)
DVG399 (ent-1(ok743) IV; sid-1(qt9);sqIs71[rgef-1p::GFP + rgef-1p::sid-1])
DVG400 (ent-1(ok743) IV; rde-1(ne300)V; neIs9[myo-3p::HA::RDE-1 + rol-6(su1006)])

Wild animals
The study did not involve wild animals.

Reporting on sex
In this study,we used hermaphrodites worms for all Caenorhabditis elegans experiments.

Field-collected samples
No field collected samples were used in the study.

Ethics oversight
We used the invertebrate C. elegans as a model organism and no ethical approval was required. According to the “Zentrale Kommission für die Biologische Sicherheit” (ZKBS), the responsible entity inside the Bundesamt für Verbraucherschutz und Lebensmittelsicherheit to assess the risk of Genetically Modified Organisms (GMO), genetic work with C. elegans is classified as risk group 1 (biological safety level 1: S1). Accordingly, we carried out our work on C. elegans in a S1-laboratory. The use of GMO in Germany is regulated by the “Gentechnik-Gesetz”, and we followed the guidelines applying to S1 work with GMO (i.e., documentation of the project and of the, exact description of the creation and maintenance of the genetic modification or correct waste treatment).

Note that full information on the approval of the study protocol must also be provided in the manuscript.

Plants

Seed stocks	N/A
Novel plant genotypes	N/A
Authentication	N/A
